# Solving the High-Intensity Multimodal Training Prescription Puzzle: A Systematic Mapping Review

**DOI:** 10.1186/s40798-024-00747-z

**Published:** 2024-07-23

**Authors:** Tijana Sharp, Katie Slattery, Aaron J. Coutts, Mikah van Gogh, Lara Ralph, Lee Wallace

**Affiliations:** 1 Faculty of Health, School of Sport, Exercise and Rehabilitation, Human Performance Research Centre, University of Technology Sydney, Ultimo, Sydney, NSW 2007 Australia; 2 Australian College of Physical Education, 10 Parkview Dr, Sydney Olympic Park, Sydney, Australia

**Keywords:** High-intensity, Multimodal training, Exercise prescription, Training outcomes, Mapping review

## Abstract

**Background:**

High-Intensity Multimodal Training (HIMT) refers to all styles of high-intensity combined aerobic, resistance and/or bodyweight exercise. Previous heterogeneity in exercise prescription and reporting in HIMT reduces the understanding of which factors should be considered when prescribing HIMT (e.g., exercise volume, intensity, duration). Previous studies have demonstrated positive effects of HIMT on health and performance outcomes. However, methodological disparities limit comparisons between findings. The objective of this systematic mapping review was to examine which prescriptive considerations and health and performance outcomes have been reported on in HIMT. This review also examined the quantity and trends of research conducted on HIMT.

**Methods:**

A systematic literature search was conducted using Ovid Medline, SPORTDiscus and Cochrane Library databases and additional sources to identify studies up until February 2023. A total of 37,090 records were retrieved, of which 220 were included for review. 246 individual HIMT protocols were included for categorical analysis against the Consensus on Exercise Reporting Template (CERT) and Applied Research Model for the Sport Sciences (ARMSS).

**Results:**

A total of 85 unique terms were used to describe HIMT. Included studies most commonly prescribed HIMT using a consistent exercise selection and circuit format. Exercise intensity was inconsistently reported on and a large proportion of studies prescribed ‘high-intensity’ exercise at a level lower than the American College of Sports Medicine criteria for high-intensity (i.e., < 77% heart rate maximum). Participation location, supervision and participation format were the most commonly reported non-training variables. The most frequently reported outcomes were cardiovascular health, perceptual outcomes, body composition and biochemical outcomes. A large proportion of previous HIMT research was experimental in design.

**Conclusions:**

Previous HIMT research demonstrates a lack of standardisation in reporting. Future studies should seek to follow guidelines (i.e., CERT) to improve reporting rigour. Additionally, forthcoming research should attempt to actively involve practitioners in implementation studies to improve ecological validity among interventions. Finally, future outcome measures should be accessible in practice and reflect common training goals of participants.

**Registration:**

This review adhered to PRISMA-ScR guidelines. Preregistration: osf.io/yknq4.

**Supplementary Information:**

The online version contains supplementary material available at 10.1186/s40798-024-00747-z.

All supplementary materials including data extracted from included studies are available online (http://osf.io/yknq4).

## Key points

- HIMT research demonstrates a lack of standardisation in reporting against guidelines (i.e., CERT). For example, exercise intensity of HIMT was inconsistently reported on and the majority of studies did not prescribe ‘high-intensity’ exercise at a level at or above current recommendations.
- Future studies should seek to involve practitioners in implementation level research to reduce barriers to the application of findings in future research and practice.
- Future assessment of outcome measures in HIMT research should represent common training goals (e.g., body composition, muscular strength) and be accessible in practical settings.

**Supplementary Information:**

The online version contains supplementary material available at 10.1186/s40798-024-00747-z.

All supplementary materials including data extracted from included studies are available online (http://osf.io/yknq4).

## Background

High-intensity exercise that combines both aerobic and muscle strengthening training stimuli into a single time efficient exercise session has recently gained interest. Previous literature has described this method of training using various terms such as high-intensity functional training, bodyweight high-intensity interval training, circuit training, resistance circuit training and CrossFit^®^ [1]. Recently, the same author group introduced the term high-intensity multimodal training (HIMT) to more broadly capture all styles of combined aerobic, resistance and/ or bodyweight training performed at a high or vigorous intensity [2]. However, there remains difficulty in quantifying and understanding the training stimulus of HIMT due to the wide variety and possible combinations of prescriptive considerations (e.g., exercise selection, order, volume, intensity). Therefore, it remains unclear which factors are or should be commonly considered when prescribing acute and chronic HIMT programs (e.g., training and non-training variables).

Previous studies have demonstrated positive effects of HIMT on health and performance outcomes when examined in isolation or compared to a control group (e.g., sedentary, no intervention, habitual activity) [3–6]. The magnitude of these effects in HIMT remains unclear when compared to other methods of combined aerobic and resistance training (i.e., concurrent training). This may be due to a lack of standardisation in exercise prescription, outcome measures and reporting methods that limit the ability to synthesise and compare previous findings. Despite recent growth in the volume of HIMT research, no group has examined all available literature to synthesise the quantity of evidence available specific to the prescriptive considerations and health and performance outcomes. Therefore, this research aims to review the literature to map the quantity and type of evidence relating to the prescriptive considerations and health and performance outcomes of HIMT. This style of review adheres to standard systematic search procedures and then categorises each study based on variables of value to both researchers and practitioners (PRISMA-ScR). Systematic mapping reviews reveal gaps in the literature and highlight areas for future research. Mapping review findings are presented in a visually appealing format to facilitate reader comprehension of the volume and density of the research topic [7, 8]. Findings of this review will identify which prescriptive considerations and health and performance outcomes have been reported on when prescribing and monitoring HIMT. This information will guide future research and assist in developing prescriptive guidelines for HIMT.

## Methods

### Search Strategy

**Fig. 1:**
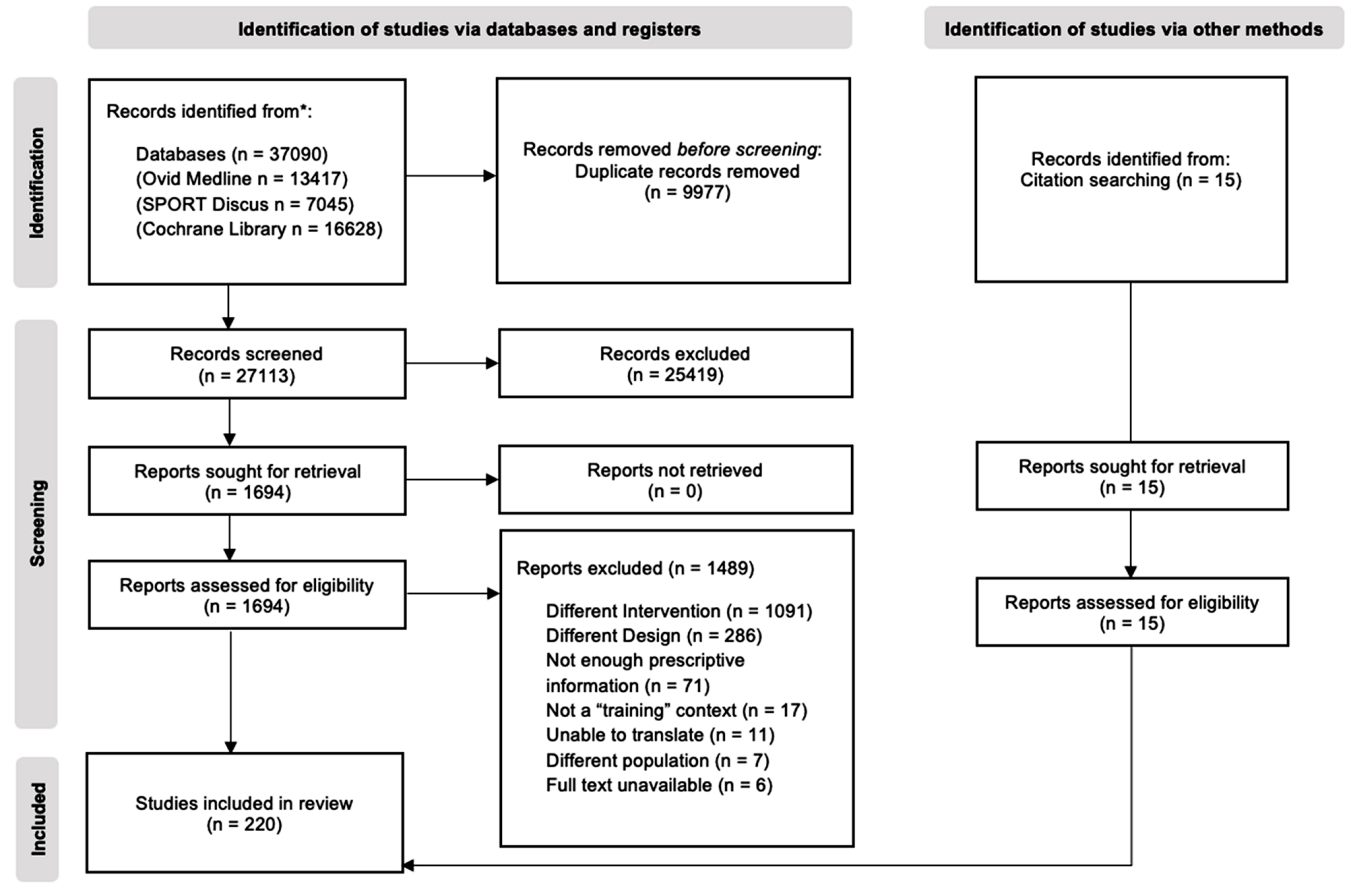
PRISMA Flow Diagram of study selection

A systematic mapping review of the literature examining HIMT in the context of exercise prescription for training was conducted using Ovid Medline, SPORTDiscus and Cochrane Library databases. Authors developed a search strategy to identify all appropriate articles using terms relevant to HIMT (Table S1). Variations of terms, operators and wildcards were used in scoping searches on each database to ensure literature saturation was achieved. Databases were searched from inception up to and including February 22, 2023. Additional, potentially relevant studies not identified in database systematic searches were detected from alternative sources (e.g., reference lists and grey literature). The search and screening processes is outlined in the Preferred Reporting Items for Systematic Reviews and Meta-Analyses extension for scoping reviews (PRISMA-ScR) diagram (Fig. 1) [9]. No medical subject headings (MeSH) were used in this search strategy due to the non-clinical nature of the review. A review protocol was registered with Open Science Framework 22 February 2023 (osf.io/yknq4).

Resultant records were uploaded to the Covidence (Veritas Health Information, Melbourne, VIC, Australia) online screening tool. Uploaded records were screened firstly by title and abstract against the eligibility criteria by two authors (TS and MvG). Conflicts were resolved by a third reviewer (LW). If it was unclear whether a study met the eligibility criteria it was included to be screened again in the following stage. The remaining full texts were screened against the eligibility criteria. Inter-rater agreement Cohen’s κ were 0.62 (88.4%) and 0.70 (92.7%) for the title/abstract and full-text screening phases, respectively. Reasons for exclusion were recorded. When full texts were irretrievable, authors screened the available information and were included if relevant. Therefore, articles without full-text availability were not excluded.

### Eligibility Criteria

This systematic mapping review included studies investigating the non-clinical population only. Studies examining participants with metabolic or chronic disease, musculoskeletal injuries or psychological disorders were excluded. Studies examining pregnant women were excluded. This review included all studies that reported on HIMT exercise prescription within a training context (in the methods section) (i.e., acute or chronic training studies). For the purposes of this review, HIMT was defined as high-intensity exercise that primarily emphasises whole-body movements and combines aerobic and muscular training (resistance or bodyweight) throughout a single exercise session [2]. Exercise protocols were excluded if methods failed to specify a high, vigorous, all out or maximal intensity of activity. Protocols that describe this level of intensity but did not monitor or provide evidence for meeting the American College of Sports Medicine (ACSM) guidelines for high-intensity activity were included. This is because both methods of exercise intensity prescription and/ or monitoring were examined in this review.

To be included studies were required to describe a minimum of the following three prescriptive variables:

- One factor related to training volume (e.g., work duration, sets, reps, rounds),
- One factor related to training intensity prescription and/ or monitoring (e.g., prescribed rating of perceived exertion [RPE], percentage heart rate maximum [%HR_max_], work rate),
- One description of the movements performed (e.g., exercise selection, HIMT format).

Studies that did not describe the prescription of HIMT for training purposes were excluded (i.e., studies that solely use HIMT session performance [e.g., reps completed, completion time] as a measurement outcome were excluded). Articles that were primarily nutritional interventions or testing studies (e.g., instrument validation studies) were also excluded. Studies that exposed participants to concurrent training (i.e., where aerobic and resistance exercise were distributed into two separate training blocks within a single session), aerobic training or resistance training only were excluded. The outcomes of this systematic mapping review were reported measures regarding health and performance (e.g., body composition, cardiovascular health, musculoskeletal health, biochemical outcomes, perceptual responses, neuro-cognitive outcomes and other physical performance outcomes).

This systematic mapping review included original research only (i.e., reviews, letters, opinion pieces, editorials, book chapters, poster or conference abstracts were excluded). The search did not restrict publication status or language. Studies written in a language other than English were translated in Google Translate (2023). For a detailed explanation of the eligibility criteria see Table S2. Given that this study was a mapping review, a rigorous quality assessment was not completed [7]. However, the level of research design of each included study was discussed in the context of the Applied Research Model for the Sport Sciences (ARMSS) [10].

### Data Extraction

In order to analyse and map the existing literature examining HIMT in the context of exercise prescription for training, data including study details (design, duration, country), population (sample size, age, training status), HIMT prescriptive considerations (training/ non-training variables) and training outcomes was extracted by a single author (TS). Categories relating to the prescriptive considerations of training were derived from the Consensus on Exercise Reporting Template (CERT) [11, 12]. The remaining CERT checklist items (e.g., adherence, fidelity) also formed categories for data extraction. Intervention details relating to HIMT only were extracted for each included study. In the event that included studies reported unique exercise prescription for progressive weeks, data for the first week of prescription was extracted and analysed. Data were entered into a bespoke online spreadsheet (enabling simultaneous review by other authors [Google Sheets]). Following extraction, a selection of randomly sampled studies was allocated to each author to cross-check data and ensure accuracy. Given mapping reviews do not require a complete synthesis of extracted data, a data summary has been provided as a supplementary material [7, 8] (Table S3).

### Data Analysis

Study categorisation based on common characteristics is a key feature of evidence mapping [7, 8]. This review identified patterns or trends within the literature using three domains of categorisation. These domains related to prescriptive considerations (i.e., training [e.g., exercise selection, order, volume, intensity] and non-training [e.g., psychosocial and environmental factors]), training outcomes (i.e., health and performance). The CERT was also used to create additional domain categories for extraction relating to the prescriptive considerations and reporting of included studies (Table S3) [11, 12]. Additionally, each study was categorised according to the level of research design according to the ARMSS [10]. Operational definitions were constructed to facilitate classification (Table 1).

**Table 1:** Operational definitions used in study categorisation

Term	Operational definition
Prescriptive considerations	A factor that has an impact on the session and/ or training outcome(s) (i.e., training and non-training variables).
Training variables	A factor that may directly impact or has a proximal relationship to the session and/ or training outcome(s) (e.g., exercise volume, intensity, duration).
Non-training variables	A factor that may be less closely associated to or has a distal relationship to the session and/ or training outcome(s) (e.g., training supervision, group environment, physical environment).
Exercise prescription	The prior planning of an exercise session or training program/ block. This includes the manipulation of key training variables such as exercise volume, intensity, work: rest ratios, exercise selection and order [13].
Exercise monitoring	Methods used to observe or measure the response to exercise during or after a session (e.g., heart rate, rating of perceived exertion) [13].
Training outcomes	Changes in health or performance related factors following participation in acute or chronic HIMT (i.e., does not include the immediate response to training e.g., heart rate, energy expenditure increases during exercise).

HIMT, *high-intensity multimodal training*

Two authors (TS and MvG) categorised studies. A third reviewer (LW) resolved any conflicts. All studies were categorised into all applicable domains. Relevant studies were categorised into more than one sub-domain. Training outcomes were categorised based on respective author or user definitions within individual studies.

## Results

### Study Selection

The initial database search generated 37,090 records and another 15 articles were identified from citation searching. Once duplicates were removed, 27,113 titles and abstracts were screened against the eligibility criteria. Of those, 25,419 were excluded. Following this, 1,694 titles were retrieved as full text and assessed for eligibility. Of those, 1,489 were excluded with reasons for exclusion displayed in Fig. 1. A complete list of excluded records including reasons is available online (Table S4, osf.io/yknq4). On completion of these procedures, 220 studies were included for analysis in this systematic mapping review.

### Study Characteristics

Of the 220 included studies, 79 studies acutely (i.e., < 4 weeks) examined HIMT, while 141 studies examined HIMT in a chronic setting (i.e., ≥ 4 weeks) respectively. Select included studies reported on multiple protocols, equating to a total of 246 unique HIMT interventions (153 = chronic, 93 = acute) (Table S3). A total of 10,415 (*n* = 5,551 females, *n* = 4,951 males) participants were involved in the included studies and 6,657 of these participants completed HIMT protocols. The age range of study participants was 9 to 72 years (Table S3).

### HIMT Terminology

Previous literature has described high-intensity combined aerobic and resistance exercise using various terms including high-intensity functional training (HIFT), bodyweight high-intensity interval training (HIIT), circuit training, extreme conditioning programs and CrossFit^®^ [1]. The most frequently reported terms included “CrossFit” (*n* = 41), “high-intensity interval training” (HIIT) (*n* = 22) and “high-intensity functional training” (HIFT) (*n* = 18). Heterogeneity in prior terminology has supported unstandardised reporting methods and large variation in exercise prescription (e.g., exercise selection, intensity, work to rest ratio). The term HIMT was defined in 2022 as an umbrella term to capture all relevant styles of this training format [2]. This term refers to all exercise modalities that emphasise whole-body movements combining aerobic, resistance and/or bodyweight training throughout a single session completed at high or vigorous intensity [2, 13]. Based on similarities to HIIT the term HIMT refers to activity performed above the moderate-intensity domain of exercise (i.e., > 77%HR_max_, > 60% heart rate reserve [HRR], > 64% maximum oxygen uptake [VO_2_max], > 14/20 RPE) and may encompass more intense training methods such as sprint interval training and ‘all out efforts’ [13, 14]. More commonly, this classification may be referred to as ‘vigorous’ intensity [15].

### Growth of HIMT Research

**Fig. 2:**
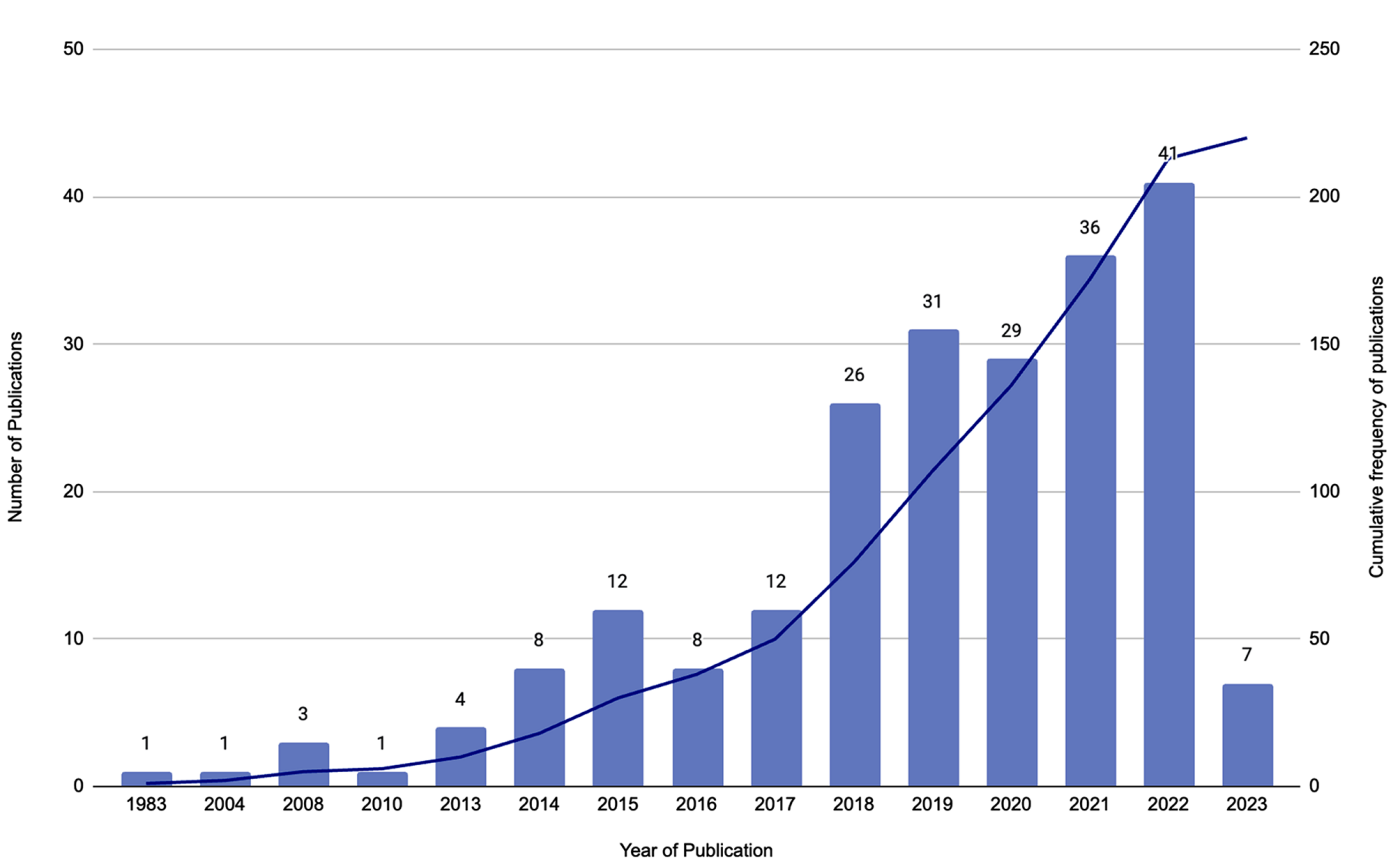
Publication frequency of included studies

The number of publications per year in HIMT has dramatically grown. There was a sharp increase in publication quantity in 2018 with a continued steady increase thereafter (Fig. 2). This trend may reflect the growing uptake of HIMT in the community and popularity of various companies and/ or businesses that deliver HIMT sessions [1]. A total of 34 countries have contributed to HIMT research (per first author affiliation). The United States of America (*n* = 61), Brazil (*n* = 34) and Spain (*n* = 24) have contributed the greatest quantity of HIMT research among the 34 countries (Table S5). Previous studies have used various unique terms to describe HIMT interventions (*n* = 85).

### Prescriptive Considerations

#### Exercise Selection & Order

**Fig. 3:**
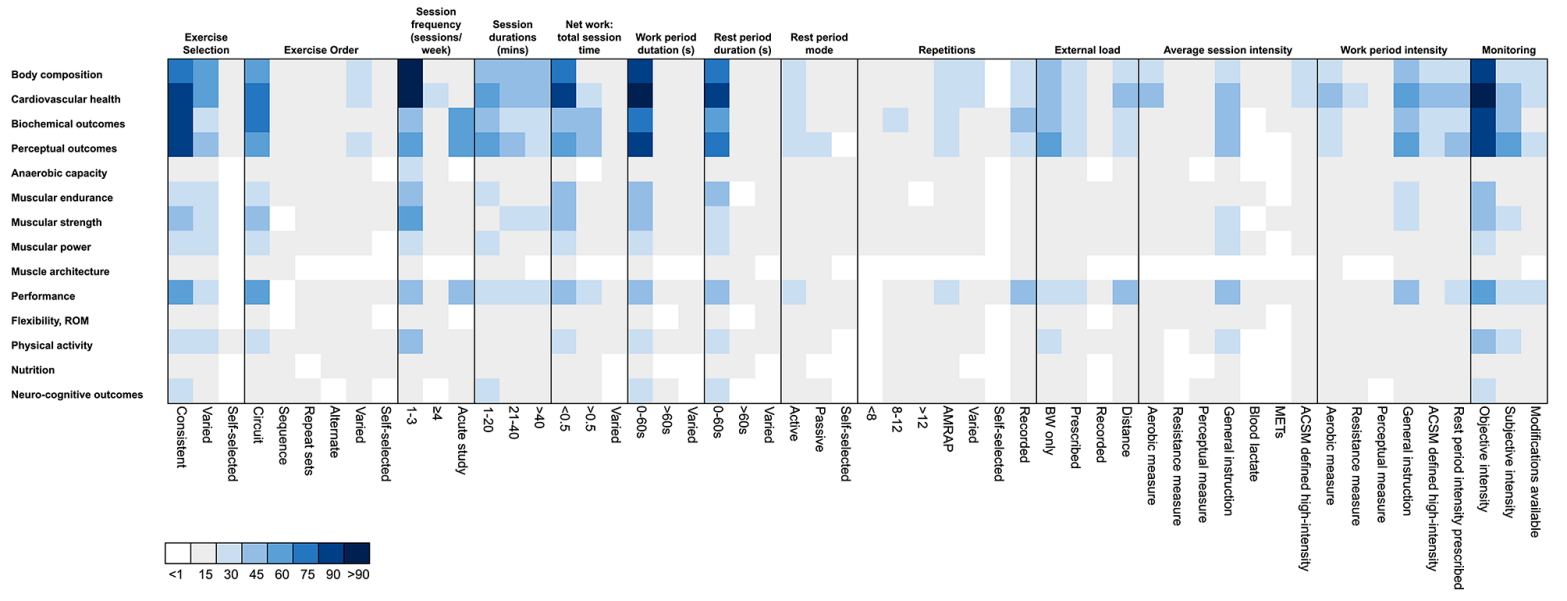
Heat map of the frequency of exercise prescription and monitoring methods (x-axis) and training outcomes reported (y-axis). Colours indicate the number of studies, whereby darker rectangles indicate higher research density ROM, *range of motion*, AMRAP, *as many rounds/ repetitions as possible*, mins, *minutes*, s, *seconds*, BW, *body weight*, METs, *metabolic equivalent of task*, ACSM, *American College of Sports Medicine*, consistent exercise selection, *exercises remained the same in each session/ training week*, varied exercise selection, *exercises varied in each session/ training week*, circuit, *repeat rounds of exercise sequences*, sequence, *exercise performed in sequence without repeating*, repeat sets, *one exercise was repeated before completing the next exercise*, alternate, *two exercises were alternated*, varied, *exercise order changed each day/ session*, aerobic measure, *(e.g., heart rate)*, resistance measure, *(e.g., percentage of repetition maximum)*, perceptual measure, *(e.g., rating of perceived exertion)*, general instruction, *(e.g., ‘all out’)*

Included HIMT protocols (*n* = 246) most commonly prescribed a selection of consistent exercises (*n* = 174) (i.e., exercises remained constant each training session or week) (Fig. 3). Exercises were most commonly prescribed in a circuit (i.e., repeat rounds of exercise sequences) format (*n* = 138) compared to varied (i.e., exercise order changed each day/ session) (*n* = 29) and repeat set formats (i.e., one exercise was repeated before completing the next exercise) (*n* = 27). Ninety-three protocols explicitly reported the use of only bodyweight resistance (Table S3).

#### Exercise Intensity

Average session and/ or work period intensity was prescribed in several ways including but not limited to %HR_max_, HRR, percentage peak oxygen uptake (%VO_2_peak), percentage of 1 repetition maximum (%1RM), RPE and general instructions (e.g., “all out”, “as many reps as possible [AMRAP]”, “as fast as possible”) [4, 5, 16–21]. The use of general instruction was the most frequently used method for both average session (*n* = 72) and work period intensity (*n* = 105). This occurred often in CrossFit^®^ HIMT protocols, where the workout of the day (WOD) may be described as a time or task priority [20, 21]. Among HIMT protocols that involved external resistance, intensity was prescribed based on percentage of repetition maximum (%RM) [22–24]. Select protocols reported prescribing specific intensities for individual exercises (i.e., 30% 1RM bench press), while others prescribed the average session intensity (e.g., 50%/ 65% 1RM for females/ males or 4-6RM) [22, 23, 25]. Among protocols that did report exercise intensity, few described an intensity at or above ACSM guidelines (based on the first session or week of prescription provided) (i.e., *n* = 41 [session intensity], *n* = 49 [work period intensity]) [13, 14]. Another 136 and 103 protocols did not report the average session and work period intensity, respectively. Finally, objective exercise intensity (e.g., HR) was monitored in 174 protocols, while subjective measures (e.g., RPE) were used in 80 protocols.

#### Exercise Volume

The average frequency of HIMT among chronic training protocols was 3 sessions/ week, while the average intervention length was 11.1 ± 8.7 weeks. The average net work (12.2 ± 12.7 min) to total session duration (30.6 ± 22.2 min) ratio was 0.46 ± 0.2. Short work (*n* = 166) and rest (*n* = 140) duration periods (0–60 s) were most commonly prescribed among included protocols, however the rest period mode was not reported among 169 protocols. Previous HIMT studies have commonly prescribed session volume based on work and rest period duration or reps and sets/ rounds and load lifted. For example, few studies described the repetition range prescribed in a numerical format (*n* = 56) while 41 protocols reported an “AMRAP” style. Prescribing volume based on duration was common in circuit or interval style HIMT and CrossFit^®^ WODs (e.g., “AMRAP”s or “for time”). Generally, a timed work period often involved instructions to work “as fast as possible” or perform the maximum number of reps possible in the given time period [19, 26, 27]. For example, previous studies described circuits involving as little as 10 seconds of work interspersed with 10 seconds of rest, compared to a 15-minute round “AMRAP” without designated rest [19, 28]. Unlike traditional aerobic HIIT, it was common for HIMT protocols to be without designated rest periods [2, 29]. Namely, CrossFit^®^, circuit and high-intensity power training (HIPT) formats of HIMT often indicate a transition between exercises rather than specified recovery period [21, 22, 24, 30–32]. Other formats of HIMT commonly provided general descriptions of rest as self-regulated, time spent waiting for a partner to complete a task, active, passive, light walking and marching [33–40]. In contrast, few HIMT formats including circuit or Tabata have prescribed rest based on objective measures (e.g., 60s of light walking, < 60% HR_max_, RPE < 5) [33, 34].

#### Progressive Overload, Regression & Modification

**Fig. 4:**
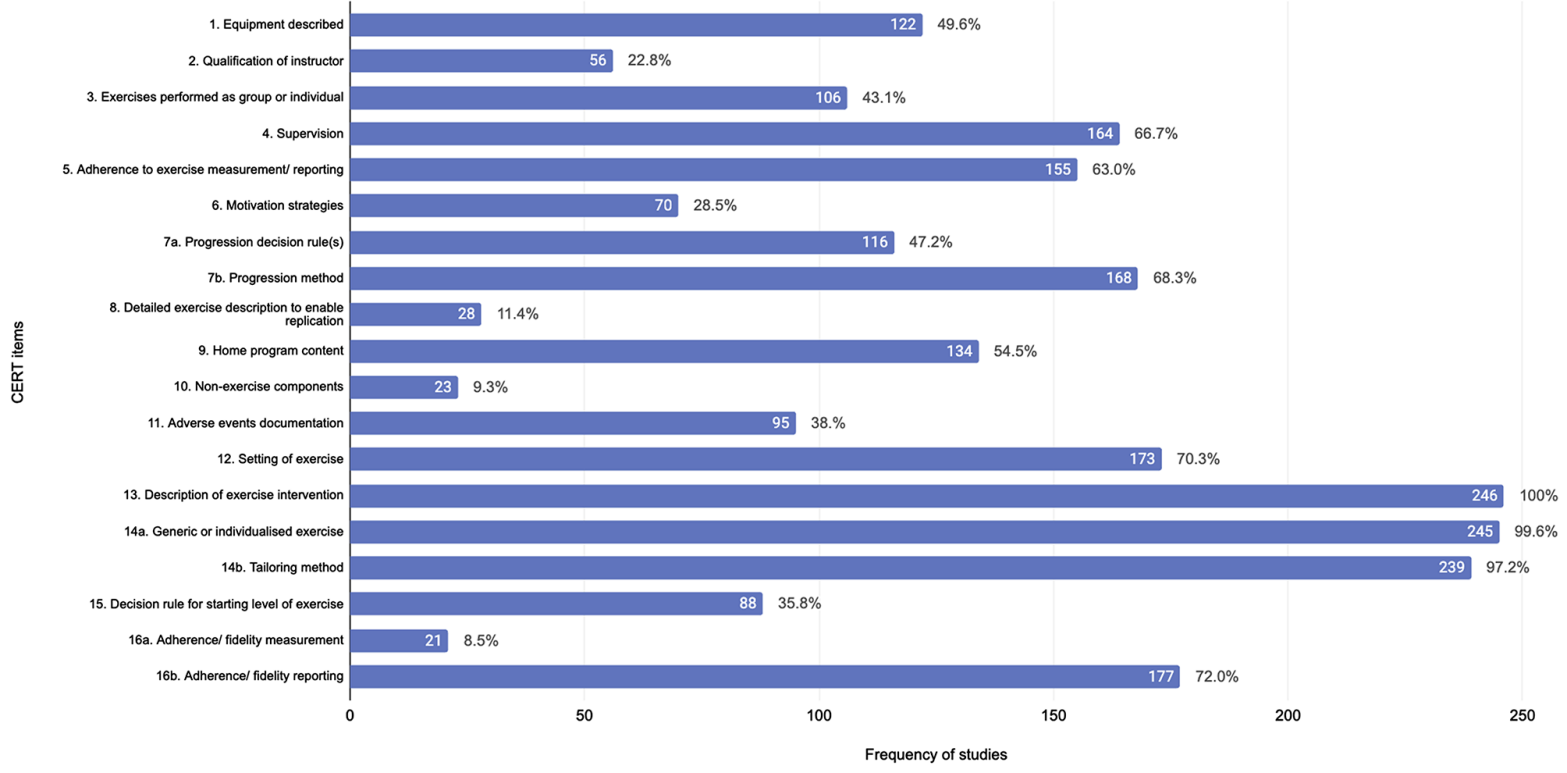
Frequency and relative percentage of HIMT protocols that reported on checklist items of the CERT. HIMT, High-Intensity Multimodal Training, CERT, Consensus on Exercise Reporting Template

Among the 153 unique chronic training protocols, 75 reported progressive overload methods throughout the intervention (e.g., volume, intensity, exercise difficulty) and 23 outlined the decision rule for progression (Fig. 4). For example, training volume was manipulated whereby the numbers of reps, sets or circuits performed was increased or the work to rest ratio was increased [3, 22, 27, 32, 33, 41–47]. Other studies have reported increasing the external load (weight) lifted [22, 25, 48]. In contrast, exercise intensity was less commonly manipulated to promote progression in HIMT programs (i.e., increase in %1RM or %HRR) [16, 49]. Only 38 protocols described modifications or scaling methods available to participants (e.g., varied box jump heights) [50]. Modifications or ‘scaled’ alternatives were often provided in HIMT (in particular CrossFit^®^) to facilitate session completion (e.g., ring row vs. kipping pull ups or single unders vs. double unders) [20, 51]. However, regression was scarcely monitored and events of scaled alternatives were often not reported [20, 51, 52]. Other authors have also described manipulating the weight lifted in order regress participants when a certain rep range was not met (i.e., < 8 reps) [53]. Additional HIMT studies reported progressing exercise complexity throughout an intervention [47, 54]. For example, a school-based CrossFit^®^ training program demonstrated progression in exercise complexity and loads lifted [54]. Despite the various reported methods of progression, few studies (*n* = 23) justified the rate or means of advancement in training stimulus (Fig. 4) [11]. These studies employed volume (e.g., repetitions reached) or perceived level of effort [18, 23, 25, 48, 55, 56].

**Fig. 5:**
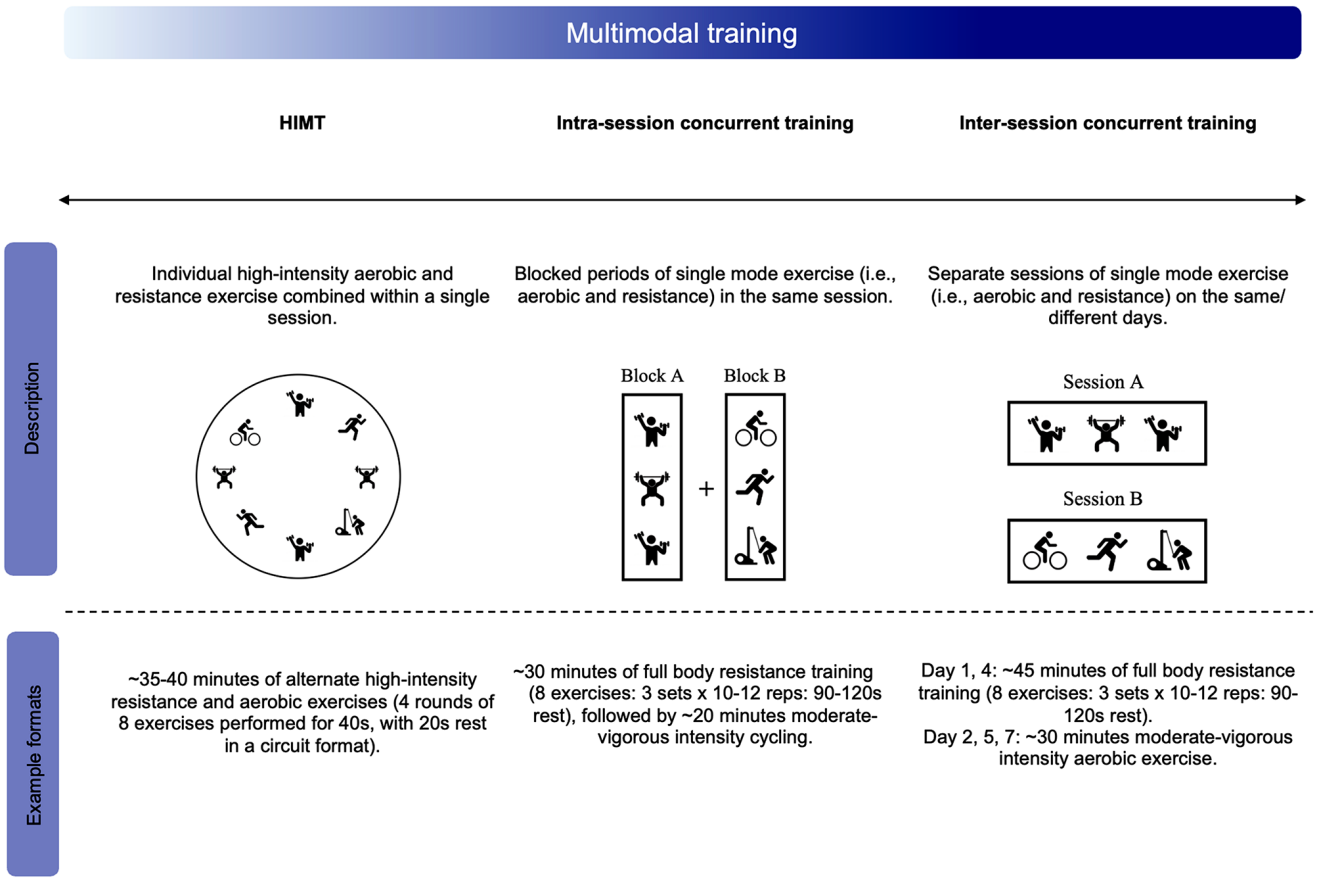
Continuum of multimodal training, HIMT, high-intensity multimodal training, HIIT, high-intensity interval training, BW HIIT, bodyweight high-intensity interval training

#### Non-Training Prescriptive Variables

Additional non-training prescriptive variables that were listed in the CERT or deemed relevant were included in analysis (Fig. 4, Table S3) [12]. It was common for protocols to report details regarding supervision (*n* = 164), however only 56 protocols provided information on the qualification of the instructor or supervisor. Motivation or encouragement strategies provided by instructors were described in 70 protocols (e.g., verbal feedback, encouragement to maintain or increase intensity) (Table S3) [4, 18, 35, 57]. The setting of the training was also commonly described (*n* = 174) where studies reported HIMT taking place indoors and outdoors, in a university gym, weights room, basketball court, fitness camp centre, online and at home [6, 24, 27, 41, 58–61]. Online and home-based HIMT featured in more recent studies, which may reflect the shift to telehealth service delivery following the COVID-19 pandemic [41, 60, 61]. Other non-training variables such as music (*n* = 27) and the intended variety (*n* = 36) of the training were less frequently described (Table S3). Group training formats (*n* = 75) were the most common participation format followed by individual (*n* = 23) and partner training (*n* = 21). Only 2 included studies published after the dissemination of the CERT in 2016 referred to the checklist in their reporting [11, 12, 62, 63] (Fig. 4).

The majority of studies in HIMT involved participants between ages 18 to 65 years (healthy adults), with few other studies including participants outside this range (Table S3) [3, 54]. For example, various physical health outcomes were examined following a school based CrossFit^®^ intervention in 10th grade students (e.g., 15–16 years) [54]. Previous HIMT studies have also involved participants of varying training status. Many HIMT investigations involved sedentary or moderately trained populations which may reflect the majority of the population participating in the community [17, 22, 23, 46, 64]. Other studies have examined a range of HIMT protocols in athletes involved in handball, soccer, basketball, hockey and bodybuilding [16, 24, 65–67]. Additional studies examining HIMT have previously involved participants with occupation or sport specific fitness and performance requirements (e.g., firefighters, or team sport athletes) [16, 24, 38, 65, 66]. These studies have often involved a greater number of task specific physical fitness and/ or performance tests compared to studies concerning the general population [38]. For example, changes in body composition, aerobic fitness, muscular endurance and power, firefighter ability and flexibility were examined following long term HIMT participation [38]. Specifically, firefighter ability was characterised by total performance in several activities (i.e., Keiser sled, Self-Contained Breathing Apparatus crawl, victim drag, hose advance, equipment carries, ladder raise). Similarly, sport specific changes in anaerobic power, anaerobic output, sprint performance, blood lactate, muscular endurance, strength, power, flexibility and shuttle run performance in handball players [16].

### Training Outcomes

Training outcomes of HIMT were categorised into several domains (Fig. 3, Table S3). Cardiovascular health (*n* = 138), perceptual outcomes (*n* = 112), body composition (*n* = 111) and biochemical markers (*n* = 106) were the most commonly reported training outcomes among included studies. For a complete list of the individual outcomes and testing protocols used see Supplementary Table S3.

#### Cardiovascular Health

Measures of cardiovascular health included maximal oxygen uptake (VO_2_max), resting HR, blood pressure (BP) and HR variability also using a variety of protocols (e.g., Bruce protocol, Balke protocol, 20 m shuttle run test, 20 m Fitness Gram Pacer test) [4, 68–70]. Other cardiovascular health tests have included a one-mile run, 6-minute walk test, time to fatigue, time trial tests, cardiovascular risk and vascular age [3, 16, 17, 28, 71].

#### Perceptual Outcomes

Perceptual outcomes included but were not limited to RPE, affective responses and perceived muscle soreness [35, 56, 72]. RPE was frequently used as an outcome measure (separate to an intensity prescriptive tool) in previous acute and chronic HIMT studies [6, 18, 51, 72–77]. However, different RPE scales (e.g., Borg 1–10, 6–20) were used, contributing to the inconsistency of reporting in HIMT [78, 79]. Additional perceptual outcomes previously examined in HIMT included exercise enjoyment, quality of life, motivation, HIIT self-efficacy, wellbeing, anxiety, sleep, psychological distress, subjective vitality, amotivation, external regulation, introjected regulation, intrinsic regulation, identified regulation, exercise initiation, exercise intentions, feelings scale, arousal, perceived stress, and various mood states [5, 6, 17, 20, 40, 41, 47, 58, 72, 73, 80–84].

#### Body Composition & Physical Activity

Measures of body composition included body weight, body fat, bone density, circumferences using a range of methods (e.g., skinfolds, dual x-ray absorptiometry, InBody scan) [46, 85, 86]. Physical activity was observed using measures such as metabolic equivalent of task (METs), resting metabolic rate, kilocalories, minutes spent exercising and step count (e.g., activity trackers) [4, 26, 31, 52, 77, 87, 88].

#### Biochemical Outcomes

Biochemical outcomes included but were not limited to lactate, creatine kinase, glucose, insulin, cholesterol, cortisol, body temperature and energy expenditure [29, 35, 89, 90]. These outcomes were more commonly observed in acute HIMT studies [39, 65, 74, 75, 91]. Common biochemical measures examined in longitudinal HIMT studies include changes in cholesterol, glucose, glucose AUC, c-reactive protein, liver enzymes [29, 52, 71, 92].

#### Musculoskeletal Outcomes

Musculoskeletal health and fitness was assessed in various ways including strength (*n* = 59) (e.g., 1RM, hand grip, 6RM, peak isokinetic torque), endurance (*n* = 45) (e.g., maximum push-up, sit-up, crunch repetitions), power (*n* = 42) (e.g., vertical jump, broad jump, medicine ball throw), and muscle architecture (*n* = 8) (e.g., pennation angle) [16, 30, 49, 60, 93–95].

#### Neurocognitive Outcomes

Neuro-cognitive outcomes such as reaction, attention and memory have been examined in HIMT. These outcomes were more commonly observed in acute settings (i.e., pre and post single session) compared to chronic HIMT interventions (e.g., autonomic nervous system function, reaction time, attention, concentration, Stroop test, Corsi test, reward positivity, accuracy, processing speed, working memory, episodic memory, visual-spatial memory, verbal ability and global cognitive function) [57, 58, 65, 96–100].

#### Other Physical Performance Outcomes

HIMT studies previously examined other physical outcomes beyond cardiovascular and musculoskeletal health. For example, lower and upper body flexibility was assessed with the sit and reach and back scratch protocols respectively [16, 38, 54, 55, 83, 101, 102]. Few studies have also examined balance (i.e., Romberg test, one standing leg test, single-leg stance), bipedal postural control and joint range of motion (e.g., ankle dorsiflexion, knee extension, hip extension, shoulder extension, glenohumeral internal rotation) [3, 47, 83, 101, 102]. Additional functional capacity tests in HIMT previously included the functional movement screen, prone timed up and go, lift and carry test, chair stand test, repeated stair climb test, and walking speed [3, 27, 61, 101, 102].

### Research Design

The 220 included studies yielded 222 research designs as two author groups reported on two investigations within one publication [103, 104]. Among these 222 designs, 46 protocols utilised a true experimental design (i.e., randomised control trial) (Table 2). Another 99 protocols identified as randomised, however did not indicate the method of randomisation used. Previous experimental studies included parallel group, randomised control trials or randomised cross-over designs [4, 55, 105]. Moreover, 64 included protocols used a quasi-experimental design, while only two study were descriptive (i.e., case study, cohort study). Quasi-experimental designs included pre-post-test, repeated measures, and cross-over designs [38, 73, 106]. These studies were commonly interested in experimentally determining the effects of an intervention on a chosen outcome. Only 11 included studies were considered to be implementation studies in the context of the ARMSS. These studies involved HIMT intervention implementation and evaluation (e.g., efficacy, feasibility) in real world settings such as schools, universities and the workplace [17, 54, 69, 107–114].

**Table 2:** Frequency of study designs and categorisation of level of research design (ARMSS)

Level of research design (ARMSS)					
**Study Design**	Descriptive	Quasi-experimental	Experimental*	Experimental	Implementation
1 group pre-post test		25			
1 group repeated measures		20			
2 arm parallel trial			51		2
2 arm parallel trial/ RCT			1	24	7
2 group pre-post test		9			
2 group repeated measures		2			
2 way cross-over		3	22	2	
3 arm parallel trial		2	12		
3 arm parallel trial/ RCT				14	2
3 group pre-post test		2			
3 way cross-over		1	7	2	
4 arm parallel trial			3		
4 arm parallel trial/ RCT				4	
4 way cross-over			1		
5 arm parallel trial			2		
Case-study	1				
Cohort	1				
**TOTAL**	2	64	99	46	11

RCT, *Randomised Control Trial*, ARMSS, *Applied Research Model for the Sport Sciences*, *, *did not report method of randomisation*

## Discussion

### Summary of Findings

This is the first systematic mapping review to synthesise the available literature examining HIMT. This review provides an overview of the quantity and type of evidence relating to the prescriptive considerations and training outcomes of HIMT. Researchers and practitioners may use these findings as a guide when developing future studies or training protocols (i.e., Table S3). Following a discussion of the trends relating to training and non-training variables in the existing literature, recommendations for future reporting of HIMT protocols are provided. Additionally, this review offers a summary of the levels of research design authors have utilised in the HIMT research. This may provide insight into possible biases in reporting methods and evidence within current HIMT literature.

### HIMT Terminology

Previous literature has used a variety of terms to describe combined high-intensity aerobic and resistance exercise. The term HIMT and associated operational definition introduced by the same author group may be more inclusive and overcome limitations of previous terms described in the literature regarding training mode description. For example, the term ‘functional’ (i.e., functional HIIT or HIFT), may be misleading in its association with motor learning, skill performance or neuromotor outcomes [115, 116]. Moreover, this term has been criticised for the lack of additional descriptive value it provides a movement or exercise. It has been suggested that many ‘functional’ movements or exercise sessions designed to replicate activities of daily life reflect traditional resistance exercise modes. This suggests the term ‘functional’ may be redundant when describing an activity mode or movement [117–119]. Therefore, the descriptor ‘multimodal’ may more appropriately highlight the combined modes of exercise, without prematurely associating the training mode with suggested outcomes. It is important to separate the term and definition from the proposed training outcomes, as the latter may be associated with the manipulation of training and non-training variables (i.e., exercise prescription). It is also critical to distinguish HIMT from other methods of multimodal training such as concurrent training. Namely, training that combines blocks of aerobic and resistance exercise into a single or separate session(s) may be understood as intra-session and inter-session concurrent training respectively (Fig. 5). In contrast, HIMT involves the combination of many individual aerobic and resistance exercises in a circuit, alternating or varied order (Fig. 3). Additionally, when distinguishing HIMT from other concurrent or single-mode training formats possible discrepancies between the intended and actual execution of exercise prescription become significant. Namely, an exercise prescribed in isolation may create primarily a resistance and/ or aerobic stimulus based on the nature of prescription. For example, a squat thrust prescribed at a slow tempo may reflect a greater resistance stimulus compared to a tempo of ‘as fast as possible’ which may indicate a bias towards an aerobic stimulus. Finally, the physical capacity (i.e., aerobic and musculoskeletal fitness) of the individual may mediate the stimulus of the training creating discrepancies between the prescribed and actual stimulus elicited. For example, low aerobic fitness may reduce the speed of execution and alter the intended stimulus from primarily aerobic to resistance. Therefore, the context of HIMT (i.e., prescription intentions) must also be considered when prescribing interventions.

Recommendations for communication relating to HIMT:

- The term multimodal may more appropriately describe the combined modes of exercise in HIMT without making suggestions about associated outcomes (e.g., motor learning, performance or neuromotor),
- The intentions or context of exercise prescription in HIMT (i.e., desired stimulus) should be considered when combining aerobic and resistance exercise.

### Training Variables & Associated Training Outcomes

Given the fundamental similarities to HIIT, it is suggested that HIMT may also elicit appropriate levels of energy expenditure and exercise intensity [31, 88, 120]. Further to this, the inclusion of resistance exercise suggests that HIMT may be a time-efficient method to meet both aerobic and resistance activity guidelines. Previous studies have also examined chronic positive health and performance outcomes of HIMT in healthy adult populations (e.g., body composition, cardiovascular health, musculoskeletal fitness, flexibility, range of motion, balance and functional capacity) [3–6, 101, 102]. While these positive outcomes are more prevalent in studies examining HIMT alone or compared to sedentary behaviour, the efficacy of HIMT compared to other combined training modes (e.g., concurrent training) remains unclear. This may be due to the large variety in previous prescription of training variables in the literature (e.g., exercise selection, order, volume, intensity).

Previous literature has examined positive outcomes of chronic HIMT participation on cardiovascular health (e.g., aerobic capacity, RHR and BP). These findings are likely attributed to the ‘high-intensity’ workloads and whole-body movements associated with HIMT [121, 122]. However, these findings may be at risk of certain biases including inconsistent prescription and monitoring of exercise intensity (i.e., prescription of intensity at a level lower than ACSM criteria [< 77% HRmax]). Additionally, many previous experimental studies are described as randomised, yet fail to report the method or type of randomisation implemented [46, 74, 92]. Other HIMT interventions have also demonstrated mixed findings for muscular fitness outcomes including strength, endurance and power [16, 27, 29, 33, 55]. The direction and magnitude of these outcomes may be related to the exercise prescription of HIMT (e.g., external load vs. bodyweight only). For example, some styles of HIMT (i.e., bodyweight HIIT) involve lifting minimal load for greater repetitions which may inhibit muscular fitness adaptations (e.g., strength) compared to protocols that involve greater external resistance (e.g., CrossFit^®^ or high-intensity power training) [20, 22]. Additionally, HIMT protocols may primarily prescribe whole-body movements throughout a session (e.g., burpee), while others may instead accumulate a whole-body stimulus across several separate exercises (e.g., squat, push up) [76, 80]. Other movements common in HIFT (i.e., HIMT) are suggested to mimic many of the most important physical tasks involved in combat or occupational tasks (e.g., lifting from the ground or overhead, pushing, pulling and/ or climbing, rotation, marching, running, change of direction) [1]. For example, previous studies describe circuit style HIFT using movements and equipment commonly used during firefighting tasks (e.g., hoses, sledgehammer) [38, 123]. Therefore, practitioners should consider the desired adaptations of training when prescribing resistance and/ or implement based exercise in HIMT to promote the appropriate training stimulus.

Recommendations for prescription/ monitoring of training variables in HIMT include:

- Ensure appropriate prescription and achievement of high-intensity exercise per ACSM guidelines to support cardiovascular adaptations (e.g., > 77% HRmax),
- Prescribe of a variety of progressions/ regressions to facilitate the maintenance of a desired exercise intensity both intra and inter exercise group/s,
- Consider the desired adaptations (e.g., strength vs. endurance, occupational task capacity) when prescribing resistance-based exercises in HIMT (i.e., bodyweight vs. external resistance, task specific implements).

### Non-Training Variables & Associated Training Outcomes

Given HIMT is a training mode commonly delivered in the community (e.g., schools, universities, fitness companies), non-training variables including biological age, training age and factors associated with a group environment are relevant to consider. Namely, participants’ age may dictate progressions/ regressions required of the training program in terms of exercise complexity and loads lifted. For example, a HIMT intervention in a younger demographic prescribed bodyweight aerobic exercises and loaded multi-joint movements performed for no longer than 30s to minimise the risk of fatigue or exhaustion. This intervention also involved a group training atmosphere, encouraging students to work in partners for select WODs [54]. In contrast, high-intensity interval bodyweight only circuit training has been observed in adults over the age of 65 [3]. This intervention focused on specific functional tests (e.g. senior fitness test and single leg balance) in addition to more common outcomes (e.g., body composition, muscular strength, endurance) [3]. This demonstrates that HIMT prescription and monitoring methods can be tailored to specific participants’ ages and associated abilities or considerations.

Training age or experience of participants may also be important to account for when prescribing HIMT at a community level due to its impact on motor learning and skill acquisition. For example, the motor skills demanded by different exercises prescribed in HIMT may vary between protocols. Specifically, an isometric wall sit hold requires greater motor capacity (e.g., muscular strength) than a wall ball throw, which in contrast may require greater motor performance and movement proficiency (e.g., coordination) [32, 64, 124]. This may impact the ability of the individual to perform each exercise efficiently and/ or safely, influencing the session and training outcomes (e.g., ability to maintain certain intensity of effort). Additionally, the suggested attenuation of muscular fitness gains caused by the interference effect (i.e., concurrent aerobic and resistance exercise) may be less applicable to individuals with a young training age vs. experienced athletes [125]. It is suggested that divergent signalling pathways of resistance and aerobic exercise are recognised as one stimulus in novice exercisers. This may contribute to greater initial adaptations despite the possibility of the interference effect occurring [125]. Therefore, this suggests that various training variables (e.g., exercise order/ selection) should be manipulated (i.e., regressed/ progressed) relative to the population participating in HIMT.

Moreover, many HIMT formats use a group exercise model which has previously been shown to support greater social capital, belongingness, social recognition and affiliation [1, 72, 126–130]. Notably, shared apparel, terminology and social norms associated with specific HIMT formats have been shown to establish feelings of identity [129, 130]. Additionally, previous findings suggest psychosocial factors related to the group training environment are associated with increased enjoyment and motivation in current HIMT participants [131]. Other HIMT styles such as CrossFit^®^ have been likened to sporting environments which may support competition, community, status and recognition [127, 132]. For example, a HIMT protocol encouraging competitiveness was previously delivered to prison inmates [133]. Additional studies have described other social formats involved in HIMT (e.g., partner exercise) [4, 5, 17, 31, 38, 72]. Specifically, one author group allowed participants to choose to participate in HIMT sessions as an individual or with a work colleague [17]. Given that HIMT is commonly supervised in a social format by a practitioner in the community, it may be relevant for future research to consider these factors more specifically to promote ecological validity. In particular, supervision has previously been suggested to promote better quality of exercise execution, increase safety and provide knowledge, feedback and support which may contribute to exercise adherence [134, 135]. Therefore, a greater understanding of the influence of supervision and encouragement on the psychosocial experience of HIMT may have implications for promoting long-term exercise behaviours.

Recommendations for prescription/ monitoring of non-training variables in HIMT include:

- Consider the possible impact that biological or training age may have on an individual’s ability to execute certain exercises at a specific exercise intensity,
- Consider the implications of the group training environment and supervision on exercise execution and other psycho-social factors (e.g., exercise adherence) to promote ecological validity in future studies,
- Clearly report features of the group training environment and supervision in future HIMT research to promote replicability.

### Challenges in Prescribing and Monitoring Training Load in HIMT

Given that HIMT is a highly demanding exercise modality which has the potential to lead to a short term overreached state (when completed over a sustained period), appropriate prescription and monitoring of training load (i.e., external/ internal) is important [136]. External load indicates the amount of training participants are exposed to (i.e., exercise selection, volume, intensity), while internal load denotes the psycho-physiological stress that occurs following this exposure (i.e., HR, RPE) [137–139]. In order to promote appropriate cardiovascular adaptations associated with HIMT, correct levels of ‘high-intensity’ activity (i.e., external load) must be prescribed and achieved. However, previous HIMT studies demonstrate an inability to consistently prescribe high or vigorous levels of intensity as defined by ACSM guidelines (based on initial prescription) (e.g., < 77% HR_max_) [1, 13, 14]. This may be attributed to challenges in describing the intensity of the multi-faceted components in HIMT prescription (i.e., combined aerobic and resistance stimulus). Likewise, the prescription and monitoring of exercise volume is another key consideration of many traditional aerobic and resistance training programs to promote appropriate training load management, thereby promoting desired adaptations, without overreaching [13]. Therefore, clarity on the best practices to prescribe and monitor both exercise intensity and volume in HIMT may promote more effective, longitudinal service delivery in the community.

A key limitation is the inability for a single metric to capture both aerobic and resistance exercise intensity in HIMT. This is likely due to the combined stimulus of HIMT (i.e., aerobic and resistance exercise) that cannot be captured by one single measure. One method that has been previously used (albeit inconsistently) to prescribe and monitor exercise intensity in HIMT is HR (i.e., %HR_max_, % HRR). For example, some studies prescribed intensity based on HR, without monitoring this during sessions [4, 16, 17]. Similarly, other studies did not prescribe intensity based on HR, but reported that HR data was collected during sessions [5, 18–21]. Although HR based methods may be accessible in the community (e.g., smart watches), this metric does not appropriately reflect the musculoskeletal, metabolic response and/ or perceptual response to HIMT (e.g., accumulated peripheral fatigue following alternate resistance and aerobic exercises). Other methods previously used to prescribe exercise intensity in HIMT include %RM or RM values. While these methods may be more appropriate for HIMT formats using external resistance (e.g., CrossFit^®^ or high-intensity power training) they are not without limitations [20, 22]. For example, previous work has described the exercise specificity of 1RM performance in resistance exercise, which may limit its applicability to whole-body exercise prescription [124, 140]. Additionally, an individual’s 1RM value has been suggested to be dynamic and influenced by changes in physiological or psychological state (e.g., residual fatigue) [141]. This is particularly relevant in HIMT, where participants may accumulate central and/ or peripheral fatigue throughout a session due to the intended high intensity level of effort, often high repetitions and reduced rest periods [142]. Therefore, the use of 1RM or predicted values may be more appropriately combined with subjective methods of exercise prescription and monitoring in HIMT to account for this [124, 143]. For example, RPE has been commonly used to prescribe exercise intensity in both aerobic and resistance exercise [13]. Given RPE captures the subjective response to exercise it may be a useful global indicator to monitor intensity in HIMT, in particular the combined training stimulus [3, 25, 27, 33, 58, 59, 74].

Two groups of authors have previously demonstrated the validity of monitoring training load with session-RPE (S-RPE) in CrossFit^®^ based HIMT [144, 145]. However, these investigations measured S-RPE at different time points following exercise (5 and 30 min respectively), which may influence participants’ perceived exertion. Additionally, Crawford and colleagues [144] demonstrated poor intra-rater reliability in participants’ ability to match their perceived exertion with the relative level of physiological effort. Despite this, there was a slight improvement in both validity and reliability as participants became accustomed to HIMT during the six-week intervention [144]. Future research should examine the use of RPE or S-RPE in other formats of HIMT (e.g., bodyweight HIIT) to more clearly understand the validity and reliability of these tools. Other subjective generalised instructions such as “all out”, "as fast as possible" or “AMRAP” have also been used to prescribe intensity in HIMT. While these general instructions may offer autonomy among participants, it greatly reduces the ability to compare between HIMT protocols and track long term training load (i.e., internal/ external). Finally, another less common method of prescribing exercise intensity in HIMT is estimated %VO_2_ based on HR [146]. While oxygen uptake may be useful in laboratory-based contexts, it is impractical in applied settings (e.g., community gyms). Therefore, future studies should attempt to determine the validity and reliability of RPE based methods in conjunction with other methods such as HR and %1RM across several formats of HIMT. Moreover, methods of prescribing and monitoring intensity must account for ecological validity and practical implementation in applied settings.

Similarly, the combined stimulus of HIMT (i.e., aerobic and resistance exercise) poses challenges for consistently prescribing and monitoring exercise volume (e.g., work period duration, repetitions, sets, load lifted). Duration based prescription (e.g., seconds, minutes) may be appropriate for certain exercises (e.g., cycle ergometer, isometric plank), while repetitions, sets and loads may be more appropriate in HIMT formats involving external resistance (e.g., integrated concurrent training, high-intensity resistance training) [16, 23, 55, 56, 147]. These discrepancies limit the ability to monitor chronic training load for individuals and compare between HIMT sessions or against other forms of combined training. This also reduces the understanding of the efficacy of HIMT for health and performance outcomes (e.g., musculoskeletal fitness). Future studies should seek to investigate appropriate volume prescription methods in HIMT. This may assist in ensuring effective prescription (i.e., external training load) for specific health and performance adaptations.

These limitations relating to the prescription and monitoring of exercise intensity and volume in HIMT also suggest that traditional linear progression methods (e.g., increased intensity with reduced volume over time) may be difficult to achieve in HIMT. Although the characteristic varied stimulus of HIMT provides numerous methods that can be concurrently employed to elicit progression (e.g., exercise difficulty, complexity, intensity, volume), it reduces the ability to monitor and control progression over time. This may pose a challenge for practitioners intending to progressively overload clients and may also reduce desire to participate among individuals seeking specific outcomes (e.g., muscular strength). However, this may not be a significant consideration for the general population perceiving HIMT as a proposed time efficient method to meet physical activity guidelines. One suggestion for monitoring progression in HIMT is to use subjective methods such as RPE to account for the dynamic nature of an individual’s physiological and psychological state [141].

Recommendations for overcoming challenges of prescribing and measuring training in HIMT:

- Investigate the validity/ reliability of subjective measures of intensity prescription and monitoring (e.g., RPE) among different HIMT styles (e.g., bodyweight HIIT vs. high-intensity resistance or power training),
- Investigate the most appropriate method/s to prescribe intensity based on the desired training stimulus,
- Investigate appropriate methods for defining, prescribing and monitoring volume in HIMT.

### Lack of Reporting According to CERT Recommendations in HIMT

Recommendations for the reporting of exercise interventions were introduced in 2016 to facilitate clearer exercise reporting, enable research replication and improve participant outcomes (i.e., CERT) [11]. Limited HIMT studies have provided appropriate levels of detail in their reporting according to these guidelines [11, 12, 62, 63]. For example, exercise selection and equipment requirements have been infrequently reported to a level of replication in HIMT (*CERT 1, 8*) (Fig. 4). Given the breadth of variety in exercise selection in HIMT, this poses challenges for comparing the efficacy of HIMT for various health and performance outcomes. Other key prescriptive and monitoring considerations such as exercise intensity (e.g., HR prescribed vs. achieved), volume (e.g., reps prescribed vs. completed) and progression/ regression have previously scarcely reported on, further contributing to these difficulties (*CERT 8, 13, 14a, b, 15*) (Fig. 4). More rigorous reporting of progression/ regression methods may be particularly relevant to practitioners required to modify the constant varied stimulus of HIMT for a range of participants.

Adhering to reporting guidelines may also be relevant to non-training variables of HIMT. For example, details regarding the qualification of the instructor may hold implications for the quality of supervision and exercise delivery participants receive (e.g., execution, safety, education, feedback, support and associated contribution to adherence) [134, 135]. Other details relating to ‘home program content’ and ‘non-exercise components’ may have an impact on the health and performance outcomes associated with HIMT (*CERT 9, 10*). For example, few studies appear to acknowledge the influence of energy intake on changes in fat or muscle mass. Specifically, nutritional intake has been scarcely reported in HIMT interventions (i.e., energy intake, macronutrients), reducing the understanding of the efficacy of HIMT for body composition changes. [4, 27, 34, 43, 44]. Therefore, future research examining changes in body composition should also attempt to control for and report on nutritional intake to allow clearer comparisons between HIMT interventions.

Future studies should endeavour to standardise reporting of protocols against guidelines (i.e., CERT) to facilitate clearer comparisons between HIMT protocols as well as with other formats of concurrent training [11]. This may facilitate matching of training protocols (i.e., dose) and a greater understanding of the effects of HIMT on health and performance outcomes. However, it must be acknowledged that stricter standardisation in reporting may increase interval validity of select HIMT protocols, yet decrease the ecological validity of others (i.e., CrossFit^®^ or workplace interventions).

Recommendations for overcoming a lack of reporting according to CERT recommendations in HIMT:

- Implement reporting guidelines (i.e., CERT) or adopted versions (i.e., Table S6) when reporting on future HIMT research [11],
- Consider the impact of strict reporting methods on interval vs. ecological validity.

### Heterogeneity in Intervention and Outcome Reporting in HIMT

Variety in exercise prescription is a key characteristic of HIMT that has been suggested to facilitate perceptions of self-efficacy and competency [17, 131, 148]. This may be attributed to certain opportunities to self-select work intensity, rest periods or exercise modifications. However, this heterogeneity in prescriptive considerations limits the ability to compare between protocols. For example, protocols have involved durations of as little as 5–8 min up to 110 min [16, 22]. The vast disparities in total and net work duration (i.e., training volume) among other training variables (e.g., exercise selection, order, intensity, progression/ regression) limit the ability to compare between HIMT protocols and other forms of concurrent training (i.e., match training dose). It also limits the understanding of the minimum effective dose for fulfilling the physical activity guidelines and/ or promoting athletic adaptations. Additionally, longer HIMT protocols may misrepresent the proposed time efficiency of HIMT or promote a state of overreaching.

Moreover, previous HIMT studies employ a wide variety of terminology and measurement tools used to examine heath and performance outcomes in HIMT further limiting the ability to make comparisons between training modes. For example, cardiovascular and aerobic fitness variables have previously included resting HR, submaximal HR, HR_max_, BP, VO_2max_, VO_2peak_, estimated VO_2_ [28, 32, 38, 46, 48, 80]. Other performance based cardiovascular health tests have previously included a one-mile run, 20 m shuttle run, 6-minute walk test, time to fatigue and time trial tests [3, 16, 17, 28]. Similar disparities are apparent in previous measures of body composition (e.g., skinfolds, girths, dual x-ray absorptiometry and InBody scans), energy expenditure (e.g., METs, resting metabolic rate, kilocalories) and physical activity in HIMT (e.g., minutes spent exercising and step count) [4, 26, 31, 46, 52, 77, 85–88]. Comparable heterogeneity is evident in previous measures of strength (e.g., 1RM, hand grip, 6RM), endurance (e.g., maximum repetitions push-ups, sit-ups, crunch) and power (e.g., vertical jump, medicine ball throw, broad jump) [16, 30, 49, 60, 93–95]. Perceptual outcomes also demonstrate large variety in HIMT (e.g., exercise enjoyment, RPE, affective responses, perceived muscle soreness) [35, 56, 72]. Future studies should attempt to examine consistent outcomes to more clearly understand perceptual responses to HIMT. In particular, possible associations between exercise intensity and enjoyment may be important to explore in HIMT given the association of exercise enjoyment and adherence in other forms of physical activity [149, 150]. The current understanding of this association is limited by conflicting findings, whereby high-intensity aerobic activity has been found to be both enjoyable and painful or unpleasurable [151, 152].

Collectively, previous outcome measures reported in HIMT demonstrate diversity, limiting comparisons between protocols and other forms of training. Future research should seek standardisation in outcomes assessed to enable a clearer understanding of the efficacy for HIMT for health and performance. Given the vast possible outcomes available to report on, it may be appropriate for future research to select accessible health and performance outcome measures in HIMT that consistently reflect training prescription goals (e.g., body composition, aerobic fitness) via collaboration with practitioners and/ or participants. This may have implications for more targeted physical activity promotion or training goals in various populations (e.g., general population vs. athletic or occupational populations).

Recommendations for overcoming heterogeneity in reporting of HIMT:

- Select accessible outcomes measures that reflect training goals (e.g., low cost, non-invasive tests).

### Recommendations for Future Research

#### Interference or ‘Interaction‘ Effect in HIMT

A key concept of HIMT that remains poorly understood due to inconsistent prescription and reporting is the impact of exercise order on training stimulus (i.e., combined aerobic and resistance exercise). Previous findings have demonstrated the impact of aerobic and resistance exercise order on training stimulus and subsequent adaptations within traditional concurrent training (Fig. 5) [153]. This phenomenon described as the interference effect, refers to attenuated musculoskeletal adaptations when aerobic and resistance exercise blocks occur subsequently within a single session [154]. This effect may occur via the reduction of the anabolic response to resistance exercise or by indirect compromise of the stimulus (due to residual fatigue or substrate depletion) [155]. Specifically, HIIT-based concurrent training (with longer duration aerobic intervals [e.g., > 1 min]) is suggested to reduce muscular strength gains but not hypertrophy [153]. However, the appropriate manipulation of training variables may reduce this effect (e.g., longer rest durations and shorter work periods of a higher intensity) [153].

It is plausible that the interference effect may be relevant to HIMT given the combination of aerobic and resistance exercises that occur within a single session (often with minimal rest periods). However, the mechanisms of this proposed effect in HIMT remain unclear, due to the complexities associated with exercise prescription. While this interference effect may be detrimental to athletes or populations with specific muscular fitness goals (i.e., explosive power or hypertrophy), it may be less relevant to the general population with more holistic training goals (e.g., general health and wellbeing). Despite the potential attenuation of specific muscular gains, HIMT stands to provide a simultaneous aerobic and resistance exercise stimulus, and proposed alternate method for individuals to meet physical activity guidelines. Furthermore, the sequenced combination of exercise modalities with reduced rest in HIMT may instead contribute to an ‘interaction’ effect, whereby greater physiological work and central or peripheral fatigue is accumulated during a session compared to traditional blocked concurrent training [142]. Minimal rest periods may promote a sustained elevation in heart rate (HR), while constant variety in exercise (aerobic vs. resistance, upper vs. lower body) may facilitate greater perceived recovery and allow participants to continue to exercise at a high or vigorous intensity. Additionally, similar forceful contractions associated with both high-intensity aerobic and resistance exercise may collaboratively support hypertrophy, despite peripheral energetic limitations [156]. Moreover, one group of authors have previously demonstrated reduced delayed onset muscle soreness (DOMS) in integrated concurrent training (i.e., HIMT) compared to serial concurrent training [56]. It was proposed that the elevated HR during aerobic exercise may increase blood flow to the skeletal muscle, which may support short term perceived recovery and long-term angiogenesis [56]. Further well-controlled research is required to understand the effects of exercise order on the potential interaction effect in HIMT and how this may impact acute session performance and chronic training outcomes.

#### Implementation Studies

In order to promote the effective transfer and uptake of research into practice, the ARMSS recommends implementation and evaluation based research in sport science [10]. Therefore, researchers should seek to conduct further implementation studies that consider how HIMT is prescribed in practical settings [10]. This will require consideration of ecologically valid approaches that may reduce internal validity of investigations (e.g., school-based intervention vs. closed exercise physiology laboratory). For example, the efficacy and feasibility of a longitudinal HIIT intervention in a school setting (i.e., Burn 2 Learn) was previously examined [35, 36]. In these process evaluations both participants and teachers involved in the intervention delivery were engaged. In order to better understand the practical implementation of HIMT, further qualitative research that examines the lived experiences and perceptions of HIMT practitioners is required. This may reveal possible barriers to the uptake of findings by practitioners or coaches and promote more effective integration of HIMT research into practice [10]. Implementation research should also seek to better understand the use of HIMT for specific training adaptations in occupational and sporting athletes (e.g., military, firefighters, team sports).

### Study Considerations

This is the first systematic mapping review to synthesise the literature concerning all formats of HIMT in a training context and identify common prescriptive methods and outcomes examined. Moreover, this review summarises the levels of research designs previously employed in HIMT research, providing insight into potential methodological and reporting bias in the literature. Also, this review refines the previous operational definition of HIMT, providing additional clarity for researchers and practitioners alike. This may facilitate clearer assessment and comparison of the various formats of HIMT in the literature. Due to the high volume of original articles retrieved in the initial search the risk for human error in the screening process must be acknowledged. To minimise this risk, two authors independently screened relevant articles and a third resolved conflicts according to PRSIMA-ScR guidelines. Limitations may also be present in possible translation errors, given articles were translated by computer software (Google Translate 2023). Additionally, the full-texts of several articles could not be translated effectively or retrieved. In this instance the abstract was screened and a judgement was made based on available information. A risk of bias may also be present in the search term selection and eligibility criteria as developed by the authors. This may have resulted in potentially eligible studies that did not use the chosen search terms to describe HIMT being overlooked. However, reference lists were searched for other relevant studies and 15 additional texts were retrieved for screening. Also, based on the eligibility criteria defined, the findings of this review are limited to studies that reported on at least one factor relating to training volume, intensity and movement. As such, several articles examining HIMT in the context of training without this level of information were excluded. Additional bias may be present in the outcomes of interest to this review, wherein “group training” was not uniquely included in the search string, therefore limiting the nature of perceptual responses captured. While it is acknowledged that searching this area of literature may provide additional valuable insights into non-training factors associated with HIMT (i.e., group training features), this search was designed to primarily focus on the prescription of HIMT.

## Conclusion

The findings of this review provide an overview of previous methods used to prescribe and monitor HIMT. Researchers and practitioners can use these findings as a tool when designing future studies or training protocols. These findings highlight a lack of standardisation in reporting in the HIMT literature. Future studies should seek to more rigorously report protocols against guidelines that consider the complexity of HIMT prescription (i.e., Table S6, CERT) to promote clearer comparisons against other formats of concurrent training. This may assist future endeavours in developing exercise prescription and monitoring guidelines for HIMT (e.g., exercise intensity, volume), similar to current recommendations for traditional resistance training, and HIIT. Additionally, future research should attempt to more clearly understand current applied methods of HIMT exercise prescription (i.e., non-training variables) and compare these methods to the literature. A collaborative research process may promote greater ecological validity in the research and as well as more effective dissemination of future findings into practice. Finally, future research should seek to examine accessible health and performance outcome measures in HIMT that appropriately represent training prescription goals (e.g., body composition, aerobic fitness). This may be relevant in promoting targeted physical activity behaviour change in various populations (e.g., general community vs. athletic or occupational populations).

### Electronic Supplementary Material

Below is the link to the electronic supplementary material.

Supplementary Material 1

Supplementary Material 2

Supplementary Material 3

Supplementary Material 4

Supplementary Material 5

Supplementary Material 6

## Abbreviations

ACSM: American College of Sports Medicine
AMRAP: As many reps as possible
ARMSS: Applied Research Model for the Sport Sciences
BP: Blood pressure
CERT: Consensus on Exercise Reporting Template
CF: CrossFit
DOMS: Delayed onset muscle soreness
HIIT: High-intensity interval training HIFT: High-intensity functional training
HIMT: High-intensity Multimodal Training
HR: Heart rate
HRR: Heart rate reserve
MET: Metabolic equivalent of task
PRISMA-ScR: Preferred Reporting Items for Systematic Reviews and Meta-Analyses extension for scoping reviews
RPE: Rating of perceived exertion
VO_2_max: Maximum oxygen uptake
WOD: Work out of the day
%1RM: Percentage of 1 repetition maximum
%HR_max_: Percentage heart rate maximum
%RM: Percentage of repetition maximum
%VO_2_peak: Percentage peak oxygen uptake
