# Supplementary material for: Solving the High-Intensity Multimodal Training Prescription Puzzle: A Systematic Mapping Review
Source: Sports Med Open. 2024 Jul 23;10:82. doi: 10.1186/s40798-024-00747-z (PMC11263329; doi:10.1186/s40798-024-00747-z)
Supplement: Supplementary file 1 — Supplementary Material 1 [file 40798_2024_747_MOESM1_ESM.pdf]

Sports Medicine Open

Title: Solving the High-Intensity Multimodal Training Prescription Puzzle: A Systematic Mapping Review.

Tijana Sharp[1](0000-0001-6878-6343); Katie Slattery[1], Aaron J. Coutts[1]; Mikah v Gogh[2], Lara Ralph[1], Lee Wallace[1]

[1]School of Sport, Exercise and Rehabilitation, University of Technology, Sydney, Human Performance Research Centre Moore Park, Sydney, Australia, [2]Australian College of Physical Education, 10 Parkview Dr, Sydney Olympic Park, Sydney, Australia

All supplementary materials including data extracted from included studies are available online (osf.io/yknq4).

Supplementary Table S1 Search strategy (22.02.2023)

Database	Search strategy	Limits	Results
**SPORTDiscus**	#1<br>TI ("weight training" OR "multimodal training" OR "multi-modal training" OR "multimodal exercise" OR "multi-modal exercise" OR "functional training" OR "functional circuit training" OR "circuit training" OR "circuit resistance training" OR "resistance training" OR "strength training" OR "cardio- resistance training" OR "cardioresistance training" OR "exercise" OR "combined training" OR "combination training" OR "calisthenics" OR "bodyweight training" OR "body weight training" OR "body weight exercise" OR "total body exercise" OR "total body training" OR "all extremity exercise" OR "high-velocity training" OR "high-velocity resistance training" OR "high-velocity resistance exercise" OR "CrossFit" OR "crossfit" OR "interval training" OR "interval exercise" OR "impact exercise" OR "impact training" OR "functional movement*" OR "interval power training" OR "weightlift*" OR "olympic lift*" OR "tabata" OR "suspension training") OR AB ("weight training" OR "multimodal training" OR "multi-modal training" OR "multimodal exercise" OR "multi-modal exercise" OR "functional training" OR "functional circuit training" OR "circuit training" OR "circuit resistance training" OR "resistance training" OR "strength training" OR "cardio- resistance training" OR "cardioresistance training" OR "exercise" OR "combined training" OR "combination training" OR "calisthenics" OR "bodyweight training" OR "body weight training" OR "body weight exercise" OR "total body exercise" OR "total body training" OR "all extremity exercise" OR "high-velocity training" OR "high-velocity resistance training" OR "high-velocity resistance exercise" OR "CrossFit" OR "crossfit" OR "interval training" OR "interval exercise" OR "impact exercise" OR "impact training" OR "functional movement*" OR "interval power training" OR "weightlift*" OR "olympic lift*" OR "tabata" OR "suspension training")<br><br>#2<br>TI ("high-intensity" OR "high intensity" OR "vigorous-intensity" OR "vigorous intensity" OR "all out" OR "AMRAP" OR "as many reps as possible" OR "as many rounds as possible" OR "as fast as possible" OR "max* effort" OR "max* exertion") OR AB ("high-intensity" OR "high intensity" OR "vigorous-intensity" OR "vigorous intensity" OR "all out" OR "AMRAP" OR "as many reps as possible" OR "as many rounds as possible" OR "as fast as possible" OR "max* effort" OR "max* exertion")<br><br>#1 AND #2	Academic articles only extracted	**7,045**
**Ovid Medline**	#1<br>(weight training or multimodal training or multi-modal training or multimodal exercise or multi- modal exercise or functional training or functional circuit training or circuit training or circuit resistance training or resistance training or strength training or cardio-resistance training or cardioresistance training or exercise or combined training or combination training or calisthenics or bodyweight training or body weight training or body weight exercise or bodyweight exercise or total body exercise or total body training or all extremity exercise or high- velocity training or high-velocity resistance training or high-velocity resistance exercise or CrossFit or crossfit or interval training or interval exercise or impact exercise or impact training or functional movement* or	N/A	**13,417**

	interval power training or weightlift* or olympic lift* or tabata or suspension training).ab,ti.<br><br>#2<br>(high-intensity or high intensity or vigorous-intensity or vigorous intensity or all out or AMRAP or as many reps as possible or as many rounds as possible or as fast as possible or max* effort or max* exertion).ab,ti.<br><br>#1 AND #2		
**Cochrane Library**	#1<br>(weight training or multimodal training or multi-modal training or multimodal exercise or multi- modal exercise or functional training or functional circuit training or circuit training or circuit resistance training or resistance training or strength training or cardio-resistance training or cardioresistance training or exercise or combined training or combination training or calisthenics or bodyweight training or body weight training or body weight exercise or bodyweight exercise or total body exercise or total body training or all extremity exercise or high- velocity training or high-velocity resistance training or high-velocity resistance exercise or CrossFit or crossfit or interval training or interval exercise or impact exercise or impact training or functional movement* or interval power training or weightlift* or olympic lift* or tabata or suspension training):ti<br><br>#2<br>(weight training or multimodal training or multi-modal training or multimodal exercise or multi- modal exercise or functional training or functional circuit training or circuit training or circuit resistance training or resistance training or strength training or cardio-resistance training or cardioresistance training or exercise or combined training or combination training or calisthenics or bodyweight training or body weight training or body weight exercise or bodyweight exercise or total body exercise or total body training or all extremity exercise or high- velocity training or high-velocity resistance training or high-velocity resistance exercise or CrossFit or crossfit or interval training or interval exercise or impact exercise or impact training or functional movement* or interval power training or weightlift* or olympic lift* or tabata or suspension training):ab<br><br>#3<br>(high-intensity or high intensity or vigorous-intensity or vigorous intensity or all out or AMRAP or as many reps as possible or as many rounds as possible or as fast as possible or max* effort or max* exertion):ti<br><br>#4<br>(high-intensity or high intensity or vigorous-intensity or vigorous intensity or all out or AMRAP or as many reps as possible or as many rounds as possible or as fast as possible or max* effort or max* exertion):ab<br><br>#5<br>#1 OR #2<br><br>#6<br>#3 OR #4	Trials only	**16628**

	#7
	#5 AND #6

N/A, not applicable
