# Supplementary material for: Solving the High-Intensity Multimodal Training Prescription Puzzle: A Systematic Mapping Review
Source: Sports Med Open. 2024 Jul 23;10:82. doi: 10.1186/s40798-024-00747-z (PMC11263329; doi:10.1186/s40798-024-00747-z)
Supplement: Supplementary file 2 — Supplementary Material 2 [file 40798_2024_747_MOESM2_ESM.pdf]

Sports Medicine Open

Title: Solving the High-Intensity Multimodal Training Prescription Puzzle: A Systematic Mapping Review.

Tijana Sharp[1](0000-0001-6878-6343); Katie Slattery[1], Aaron J. Coutts[1]; Mikah v Gogh[2], Lara Ralph[1], Lee Wallace[1]

[1]School of Sport, Exercise and Rehabilitation, University of Technology, Sydney, Human Performance Research Centre Moore Park, Sydney, Australia, [2]Australian College of Physical Education, 10 Parkview Dr, Sydney Olympic Park, Sydney, Australia

All supplementary materials including data extracted from included studies are available online (osf.io/yknq4).

Supplementary Table S2 Eligibility Criteria

	Inclusion Criteria	Exclusion Criteria
**Population**	Non-clinical populations.<br>Any age.<br>Any gender.	Clinical populations (i.e., individuals requiring assessment and clearance to exercise by a health professional such as a general practitioner or exercise physiologist due to their condition or pathology).<br><br>Animal subjects.
**Exposure**	HIMT (i.e., exercise that primarily emphasises whole-body movements and combines aerobic and muscular training [resistance or bodyweight] throughout a single exercise session [1]. Exercise protocols were excluded if methods failed to specify a high, vigorous, all out or maximal intensity of activity. Protocols that describe this level of intensity but did not monitor or provide evidence for meeting the ACSM guidelines for high-intensity activity were included).<br><br>To be included studies were required to describe a minimum of the following three prescriptive variables:<br>• One factor related to training volume (e.g., work duration, sets, reps, rounds),<br>• One factor related to training intensity prescription and/ or monitoring (e.g., prescribed RPE, $\%HR_{max}$, work rate [AMRAP]),<br>• One description of the movements performed (e.g., exercise selection, HIMT style).<br>Studies that included more than the above three prescriptive variables were included.	Concurrent training (i.e., where aerobic and resistance exercise were distributed into separate training blocks within a single session).<br><br>Single modular circuit training (i.e., resistance or aerobic exercise only performed in a circuit fashion).<br><br>CrossFit studies that prescribed only single-modular exercise sessions.<br><br>Plyometric training studies that only prescribed plyometric exercises.<br><br>Aerobic high-intensity interval training (HIIT).<br><br>High-intensity resistance training (HIRT) (i.e., where only resistance exercises were prescribed).<br><br>Studies that did not provide enough prescriptive information (e.g., did not report exercises prescribed).
**Outcomes**	Health and performance (e.g., body composition, cardiovascular health, musculoskeletal health, biochemical outcomes, subjective responses, neuro-cognitive outcomes and sport/ occupation specific performance).	n/a
**Purpose**	Acute training studies.<br>Chronic training studies.	Nutritional intervention.<br>Testing studies.

**Design**	Original research.<br>Experimental studies.<br>Quasi-experimental studies.<br>Descriptive studies.	Reviews.<br>Opinion pieces.<br>Letters.<br>Editorials.<br>Book chapters.<br>Conference or congress abstracts.

References:

1. Sharp T, Grandou C, Coutts AJ, Wallace L. The Effects of High-Intensity Multimodal Training in Apparently Healthy Populations: A Systematic Review. Sports Med Open. 2022 Mar 29;8(1):43. doi: 10.1186/s40798-022-00434-x.
