# Supplementary material for: Solving the High-Intensity Multimodal Training Prescription Puzzle: A Systematic Mapping Review
Source: Sports Med Open. 2024 Jul 23;10:82. doi: 10.1186/s40798-024-00747-z (PMC11263329; doi:10.1186/s40798-024-00747-z)
Supplement: Supplementary file 3 — Supplementary Material 3 [file 40798_2024_747_MOESM3_ESM.pdf]

Sports Medicine Open

Title: Solving the High-Intensity Multimodal Training Prescription Puzzle: A Systematic Mapping Review.

Tijana Sharp1(0000-0001-6878-6343); Katie Slattery1, Aaron J. Coutts1; Mikah v Gogh2, Lara Ralph1, Lee Wallace1

1School of Sport, Exercise and Rehabilitation, University of Technology, Sydney, Human Performance Research Centre Moore Park, Sydney, Australia, 2Australian College of Physical Education, 10 Parkview Dr, Sydney Olympic Park, Sydney, Australia

All supplementary materials including data extracted from included studies are available online (osf.io/vkmq6).

STUDY / DEMOGRAPHIC INFORMATION

Acute/ chronic (4 weeks)	Author	Title	Year	Nation_Auth1ARI	Nation_ARI2	Nation_ARI3	Nation_ARI4	Nation Participants	Chronic HIMT duration (weeks)	Acute HIMT duration (sessions)	Sample Size (total n)	HIMT (n)	Males (n)	Females (n)	Age (years)	Age (years)	Age (years)	Age (years)
CHRONIC	Ajjimaporn et al. 2019	Effect of 4-Week HICTBW Training	2019	Thailand				Thailand	4		26	12	0	26	35 ± 3			
CHRONIC	Ajjimaporn et al. 2023	Body composition and	2023	Thailand	UK			Thailand	8		26	12	0	26	31 ± 2 (HICTBW)	32 ± 3 (CG)		
CHRONIC	Alberto 2018	24 sessions of monitored	2018	Spain				Spain	12		184	90	98	86	13.73 ± 1.34			
CHRONIC	Alonso-Fernandez et al. 2017a	Effects of a high-intensity	2017	Spain				Spain	8		14	7	0	14	15.2 ± 0.6			
CHRONIC	Alonso-Fernandez et al. 2017b	Effect of a HIIT programme vs.	2017	Spain				Spain	10		32	16	16	16	22.37 ± 1.8			
CHRONIC	Alonso-Fernandez et al. 2019	Impact of a HIIT protocol on body	2019	Spain				Spain	7		26	13	13	13	15-16			
CHRONIC	Alonso-Fernandez et al. 2022	Impact of High-Intensity	2022	Spain				Spain	8		21	11	11	10	35.4 ± 5.6			
ACUTE	Alves Nazzi et al. 2018	Acute responses of high-intensity	2018	Brazil				Brazil		1	12	12	0	12	23.0 ± 4.7 (low physical fitness)	23.6 ± 2.9 (high physical fitness)		
CHRONIC	Artese et al. 2021	Effect of Functional	2021	USA				USA	24		44	21	0	44	60.3 ± 8.3			
CHRONIC	Asilah Za'don et al. 2019	High-intensity interval training	2019	Malaysia				Malaysia	12		50	25	25	25	29 ± 5			
ACUTE	Babiash 2013*	Determining the energy	2013	USA				USA		1	15	15	7	8	28.4 ± 9.27 (F)	30.7 ± 8.60 (M)		
CHRONIC	Bahremand et al. 2020*	A comparison of CrossFit and	2020	Iran				Iran	8		30	16	0	30	30.8 ± 6.9 (CF)	30.9 ± 6.8 (CT)		
CHRONIC	Ballesta-Garcia et al. 2019	High-intensity Interval Circuit	2019	Spain				Spain	18		54	18	0	54	67.8 ± 6.2			
ACUTE	Barreto et al. 2023*	Heart rate variability and	2023	Brazil	Portugal			Portugal		1	10	10	10	0	29.6 ± 3.5			
CHRONIC	Battucci et al. 2022	Impact of Intermittent	2022	Brazil				Brazil	8		36	26	0	36	32.2 ± 6.6			

ACUTE	Nunez et al. 2020	Metabolic effects of two	2020	USA				USA		1	16	16	16	0	25.7 ± 4.6	[illegible]
ACUTE	Pantaleao et al. 2022	A single functional	2022	Brazil	Australia			Brazil		1	23	23	16	9	21.4 ± 3.8	[illegible]
CHRONIC	Paoli et al. 2010	Effects of three distinct protocols	2010	Italy				Italy	12		40	10	_	_	56 ± 2.7	[illegible]
CHRONIC	Paoli et al 2013	Effects of high-intensity	2013	Italy				Italy	12		58	19	58	0	61 ± 3.3	[illegible]
ACUTE	Pearson et al. 2020	Tabata-style functional	2019	USA				USA		1	11	11	11	0	23 ± 2.68	[illegible]
CHRONIC	Popowczak et al. 2022	The high-intensity	2022	Poland				Poland	10		141	73	52	89	16.12 ± 0.42 (F)	[illegible]
CHRONIC	Posnakidis et al. 2022a	High-Intensity Functional	2022	Greece				Greece	8		13	13	5	8	28.3 ± 3.8	[illegible]
CHRONIC	Posnakidis et al. 2022b	The Addition of High-Load	2022	Greece				Greece	8		20	20	8	12	30 ± 4	[illegible]
CHRONIC	Posnakidis et al. 2022b															[illegible]
CHRONIC	Quindry et al. 2020	Health and fitness benefits	2020	USA				USA	8		23	23	8	17	38.0 ± 1.7	[illegible]
CHRONIC	Ramirez-Velez et al. 2020	Effects of Different Doses	2020	Spain	Chile	Colombia		Colombia	26 (6 months)		95	21	28	67	13.5 ± 1.6	[illegible]
CHRONIC	Rengers et al. 2021	Effects of High-Intensity	2021	USA	Canada			USA	12		16	9	0	16	23.0 ± 5.9	[illegible]
ACUTE	Reppa et al. 2023	The Effect of Aerobic Fitness	2023	Greece				Greece		1	28	28	0	28	24.2 ± 1.5	[illegible]
ACUTE	Ricci et al. 2021	Acute Cardiometabolic	2021	USA				USA		2	19	19	0	19	9.1 ± 1.1	[illegible]
ACUTE	Ricci et al. 2021															[illegible]
CHRONIC	Ricci et al. 2022	Program Evaluation and	2022	USA				USA	6		67	45	25	42	10.5 ± 0.9	[illegible]
ACUTE	Riegler et al. 2017	Acute Responses to the 7-Minute	2017	USA				USA		1	14	14	7	7	25.4 ± 8.8	[illegible]
CHRONIC	Romero-Arenas et al. 2018	Neuromuscular and	2018	Spain				Spain	6		29	10	29	0	23.1 ± 2.7	[illegible]
CHRONIC	Ruiz-Ariza et al. 2019	The effect of cooperative	2019	Spain				Spain	12		184	90	98	86	13.73 ± 1.34	[illegible]
CHRONIC	Sanchez Perez & Gabriel Angel	Effects of Tabata Training in the	2015	Spain				Spain	8		16	9	16	0	17.8 ± 1.3	[illegible]
CHRONIC	Schaun & Alberton 2022	Using Bodyweight as	2022	Brazil	USA			Brazil	16		41	12	41	0	23.7 ± 0.7	[illegible]
ACUTE	Schaun & Del Vecchio 2018	High-Intensity Interval	2018	Brazil				Brazil		1	12	12	12	0	23.3 ± 3.9	[illegible]
CHRONIC	Schaun et al. 2018	Whole-Body High-Intensity	2018	Brazil				Brazil	16		55	19	55	0	23.7 ± 0.7	[illegible]
CHRONIC	Schaun et al. 2019	Neuromuscular adaptations to	2019	Brazil				Brazil	16		41	12	41	0	23.73 ± 0.65	[illegible]
ACUTE	Schlegel et al. 2020	Physiological Response to	2020	Czech Republic				Czech Republic		1	1	1	1	0	35	[illegible]
ACUTE	Schleppenbach et al. 2017	Speed- and Circuit-Based	2017	USA				USA		1	22	22	7	15	21.36 ± 2.65	[illegible]
CHRONIC	Schmidt et al. 2016	The effect of high-intensity	2016	USA				USA	8		96	60	43	53	20.7 ± 1.3 (CT-7F)	[illegible]
ACUTE	Scott et al. 2016	Total energy costs of 3 all-out	2016	USA				USA		4	15	15	11	4	22.1 ± 1.9	[illegible]
ACUTE	Scott et al. 2016															[illegible]
CHRONIC	Scott et al. 2019	Home-hit improves muscle	2019	UK				UK	12		32	9	13	19	36 ± 10	[illegible]
CHRONIC	Sian et al. 2021	Short-Term, Equipment-Free	2021	UK				UK	4		30	20	15	15	24 ± 5	[illegible]
CHRONIC	Sian et al. 2021															[illegible]
CHRONIC	Sian et al. 2022	Equipment-free, unsupervised	2022	UK				UK	4		30	20	14	16	71 ± 5	[illegible]
CHRONIC	Sian et al. 2022															[illegible]
CHRONIC	Silva et al. 2022	Effects of High-Intensity	2022	Portugal	Iran	Romania	Poland	Iran	8		18	9	18	0	20.4 ± 0.7	[illegible]
ACUTE	Smith et al. 2022	The Effects of a Single Session of	2022	USA	New Zealand			New Zealand		1	13	13	5	8	47 ± 10	[illegible]
CHRONIC	Sobrero et al. 2017	A Comparison of High Intensity	2017	USA				USA	6		19	8	0	19	26.0 ± 7.31 (HIFT)	[illegible]
CHRONIC	Soltani et al. 2020a	Meta-inflammatory state and	2020	Iran				Iran	10		26	13	0	26	18-25	[illegible]
CHRONIC	Soltani et al. 2020b	Combined All-Extremity	2020	Iran				Iran	10		26	13	0	26	20.38 ± 1.5 (CHIIT)	[illegible]
ACUTE	Soltani et al. 2020c	Assessment of the Effect of	2020	Iran				Iran		10	26	13	0	26	21.3 ± 1.37 (CHIIT)	[illegible]
CHRONIC	Songsorn et al. 2022	The effect of whole-body	2022	Thailand				Thailand	6		21	10	15	6	22.0 ± 0.8 (WB-HIIT)	[illegible]
CHRONIC	Sperlich et al. 2017	Functional High-Intensity	2017	Germany	Canada	Norway		Germany	9		19	11	0	19	23 ± 2	[illegible]
ACUTE	Sperlich et al. 2018a	Prolonged Sitting Interrupted by	2018	Germany	Belgium	Norway	Sweden	Germany		1	12	12	5	7	22 ± 2	[illegible]
CHRONIC	Sperlich et al. 2018b	A 4-Week Intervention	2018	Germany	Sweden	Canada	Norway	Germany	4		26	24	10	14	25 ± 5	[illegible]
CHRONIC	Sperlich et al. 2018b															[illegible]
ACUTE	Streetman et al. 2022	Anticipatory Anxiety	2022	USA				USA		4	16	16	6	10	20.2 ± 1.14	[illegible]
CHRONIC	Taal et al. 2016	Feasibility study into	2016	Netherlands				Netherlands	12		15	15	7	8	53 ± 10	[illegible]
CHRONIC	Takeshima et al. 2004	Effect of concurrent	2004	Japan	USA			Japan	12		35	18	15	20	68.3 ± 4.9 (PACE)	[illegible]
ACUTE	Thomas et al. 2017	Acute exercise and motor	2017	Denmark	USA	Canada		Denmark		1	40	10	40	0	25.3 ± 3.6	[illegible]
CHRONIC	Tian et al. 2021	The Efficacy of Brief	2021	China				China		26 (6 months)	164	64	92	72	12.2 ± 0.6 (HBHEx)	[illegible]
ACUTE	Tibana et al. 2016	Two Consecutive Days of Extreme	2016	Brazil	USA			Brazil		2	9	9	9	0	26.7 ± 6.6	[illegible]
ACUTE	Tibana et al. 2016															[illegible]
ACUTE	Tibana et al. 2018*	Lactate, Heart Rate and Rating	2018	Brazil				Brazil		1	9	9	9	0	27.7 ± 3.2	[illegible]
ACUTE	Tibana et al. 2022	Time-course effects of	2022	Brazil	Canada			Brazil		2	8	8	8	0	28.1 ± 5.4	[illegible]
ACUTE	Tibana et al. 2022															[illegible]
CHRONIC	Timmons et al. 2023	Increased Lean Body Mass After	2022	Ireland	USA			Ireland	8		18	9	18	0	25.7 ± 4.3	[illegible]
ACUTE	Timon et al. 2019	48-hour recovery of biochemical	2019	Spain				Spain		1	12	12	12	0	30.4 ± 5.37	[illegible]
ACUTE	Toledo et al. 2021	Comparison of Physiological	2021	Brazil	Portugal	USA		Brazil		2	23	23	13	10	26.5 ± 4.3	[illegible]
ACUTE	Toledo et al. 2021															[illegible]
CHRONIC	Tottori et al. 2019	Effects of High Intensity Interval	2019	Japan				Japan	4		56	27	31	25	10.0 ± 1.0 (HIIT)	[illegible]
CHRONIC	Valkenborghs et al. 2022	Effect of high-intensity	2022	Australia	USA			Australia	26 (6 months)		56	30	21	35	16.1 ± 0.4	[illegible]
CHRONIC	Vliedtra et al. 2023	High-intensity interval aerobic	2023	New Zealand	USA			New Zealand	20		82	41	12	70	64.8 ± 3.2 (HIART)	[illegible]
ACUTE	Walsh et al. 2018	Brief, High-Intensity	2018	Canada	USA			Canada		1	22	22	3	19	20.0 ± 1.0	[illegible]
ACUTE	Walsh et al. 2018									1	23	23	6	17	19.0 ± 0.5	[illegible]
ACUTE	Walsh et al. 2019	High-intensity interval exercise	2019	Canada				Canada		1	25	25	9	16	22.4 ± 3.5	[illegible]
CHRONIC	Wibowo et al. 2021	The effect of a short term high	2021	Indonesia				Indonesia	6		24	12	24	0	27.50 ± 0.95 (AMRAP)	[illegible]
CHRONIC	Wibowo et al. 2022	Endurance cardiovascular,	2022	Indonesia				Indonesia	6		30	30	30	0	26.14 ± 0.95 (AMRAP)	[illegible]
CHRONIC	Wibowo et al. 2022															[illegible]
CHRONIC	Wibowo et al. 2022															[illegible]
ACUTE	Wilke & Roye 2020	Exercise Intensity May Not	2020	Germany				Germany		1	24	24	11	13	26 ± 3	[illegible]
ACUTE	Wilke 2020	Functional high-intensity	2020	Germany				Germany		1	35	35	17	18	26.7 ± 3.6	[illegible]
CHRONIC	Wilke et al. 2019	Effects of high-intensity	2019	Germany				Germany	6		33	20	12	21	25 ± 5	[illegible]
ACUTE	Willis et al. 2019	Energy Expenditure and	2019	USA				USA		3 (max)	20	20	10	10	31.4 ± 9.2	[illegible]
CHRONIC	Winn et al. 2021	Effect of high-intensity	2021	UK				UK	26 (6 months)		616	221	334	282	13.0 ± 1.1	[illegible]
ACUTE	Yang et al. 2018	High-intensity Intermittent	2018	Taiwan				Taiwan		1	9	9	9	0	22 ± 1.3	[illegible]
CHRONIC	Zhang et al. 2021	Effects of Online Bodyweight	2021	China	Canada			China	6		70	33	0	70	22.61 ± (HIIT)	[illegible]
CHRONIC	Ziyaiyan et al. 2023	High-intensity circuit training	2023	Iran	Germany	Qatar	Tunisia	Iran	8		21	11	21	0	15.0 ± 3.4	[illegible]
