# Supplementary material for: Solving the High-Intensity Multimodal Training Prescription Puzzle: A Systematic Mapping Review
Source: Sports Med Open. 2024 Jul 23;10:82. doi: 10.1186/s40798-024-00747-z (PMC11263329; doi:10.1186/s40798-024-00747-z)
Supplement: Supplementary file 4 — Supplementary Material 4 [file 40798_2024_747_MOESM4_ESM.pdf]

Sports Medicine Open

Title: Solving the High-Intensity Multimodal Training Prescription Puzzle: A Systematic Mapping Review.

Tijana Sharp1(0000-0001-6878-6343); Katie Slattery1, Aaron J. Coutts1; Mikah v Gogh2, Lara Ralph1, Lee Wallace1

1School of Sport, Exercise and Rehabilitation, University of Technology, Sydney, Human Performance Research Centre Moore Park, Sydney, Australia, 2Australian College of Physical Education, 10 Parkview Dr, Sydney Olympic Park, Sydney, Australia

All supplementary materials including data extracted from included studies are available online (osf.io/yknq4).

Title
A 2-week combined high-intensity interval training regulates inflammatory status in young females with obesity
A 3-week multimodal intervention involving high-intensity interval training in female cancer survivors: a randomized controlled trial
4 weeks of high-intensity interval training does not alter the exercise-induced growth hormone response in sedentary men
A 4-week exercise and protein program improves muscle mass and physical functioning in older adults - A pilot study
A 10-Week Block of Combined High-Intensity Endurance and Strength Training Produced Similar Changes in Dynamic Strength, Body Composition, and Serum Hormones in Women and Men
12min/week of high-intensity interval training reduces aortic reservoir pressure in individuals with metabolic syndrome: a randomized trial
A 16-week circuit interval-based exercise intervention reduces systemic inflammation in obese, sedentary cancer survivors
Acceleration of VO2 kinetics in heavy submaximal exercise by hyperoxia and prior high-intensity exercise
Activating brown adipose tissue through exercise (ACTIBATE) in young adults: Rationale, design and methodology
Acute affective responses to high-intensity interval exercise: Implications on the use of different stimulus-recovery amplitudes
Acute and Post-Exercise Physiological Responses to High-Intensity Interval Training in Endurance and Sprint Athletes
Acute appetite and eating behaviour responses to apparatus-free, high-intensity intermittent exercise in inactive women with excess weight
Acute Caffeine Supplementation Does Not Improve Performance in Trained CrossFit R Athletes
Acute Cardiopulmonary and Metabolic Responses to High-Intensity Interval Training Protocols Using 60 s of Work and 60 s Recovery
Acute Cardiopulmonary Response to Interval and Continuous Exercise in Older Adults: A Randomized Crossover Study
Acute cardiopulmonary responses to strength training, high-intensity interval training and moderate-intensity continuous training
Acute changes in glycemic homeostasis in response to brief high-intensity intermittent exercise in obese adults
Acute continuous moderate-intensity exercise, but not low-volume high-intensity interval exercise, attenuates postprandial suppression of circulating osteocalcin in young overweight and obese adults
Acute effect of brief low- and high-intensity exercise on circulating insulin-like growth factor (IGF) I, II, and IGF-binding protein-3 and its proteolysis in young healthy men
Acute effect of high intensity interval training (Hiit) on testosterone levels, cortisol and testosterone on cortisol ratio in obese and overweight children untrained and trained
Acute effect of high-intensity interval exercise and moderate-intensity continuous exercise on appetite in overweight/obese males: a pilot study
Acute effect of high-intensity interval exercise on vascular endothelial function and possible mechanisms of wall shear stress in young obese males
Acute Effect of Intermittent Exercise and Action-Based Video Game Breaks on Math Performance in Preadolescent Children
The Acute Effect Of Mindful High-intensity Interval Training On Inhibitory Control, Attention, And Cognitive Flexibility: 246
Acute effect of three different exercise training modalities on executive function in overweight inactive men: A secondary analysis of the BrainFit study
Acute effects of aerobic exercise on mood and hunger feelings in male obese adolescents: a crossover study
The Acute Effects of Different Exercise Intensities on Associative Novel Word Learning in Healthy Older Adults: A Randomized Controlled Trial
Acute Effects of Different Plyometric and Strength Exercises on Balance Performance in Youth Weightlifters
Acute Effects of Exercise Intensity on Insulin Sensitivity under Energy Balance
Acute Effects of High Intensity, Resistance, or Combined Protocol on the Increase of Level of Neurotrophic Factors in Physically Inactive Overweight Adults: The BrainFit Study
Acute effects of high-intensity exercise on hematological and iron metabolic parameters in elite male and female dragon boating athletes
Acute Effects of High-Intensity Interval and Moderate-Intensity Continuous Exercise on GLP-1, Appetite and Energy Intake in Obese Men: A Crossover Trial
Acute Effects of High-Intensity Interval Exercise While Wearing a Sauna Suit on Energy Expenditure and Excess Post-exercise Oxygen Consumption
Acute effects of high-intensity interval training and moderate-intensity continuous exercise on BDNF and irisin levels and neurocognitive performance in late middle-aged and older adults
The acute effects of high-intensity interval training and moderate-intensity continuous exercise on declarative memory and inhibitory control
Acute effects of high-intensity interval training and moderate-intensity continuous training on arterial stiffness in young obese women
Acute effects of high-intensity interval training session and endurance exercise on pulmonary function and cardiorespiratory coupling
Acute effects of high-intensity interval, resistance or combined exercise protocols on testosterone - cortisol responses in inactive overweight individuals
Acute effects of highly intense interval and moderate continuous exercise on the modulation of neural oscillation during working memory
Acute Effects of Intermittent Physical Activity on Perceptions of Exercise-Related Fatigue in Children
Acute effects of interval versus continuous endurance training on pulse wave reflection in healthy young men
The Acute Effects of Milk Consumption on Systemic Inflammation after Combined Resistance and Plyometric Exercise in Young Adult Females
Acute Effects of Oatmeal on Exercise-Induced Reactive Oxygen Species Production Following High-Intensity Interval Training in Women: A Randomized Controlled Trial
Acute Effects of Physical Exercise on Cognitive and Psychological Functioning in College Students with Attention-Deficit/Hyperactivity Disorder
Acute Effects of Sprint Interval Training and Chronic Effects of Polarized Training (Sprint Interval Training, High Intensity Interval Training, and Endurance Training) on Choice Reaction Time in Mountain Bik[illegible]
Acute Effects of Two Different Resistance Circuit Training Protocols on Performance and Perceived Exertion in Semiprofessional Basketball Players
Acute effects of two different work-to-rest ratio of high-intensity interval training on brain-derived neurotrophic factor in untrained young men
Acute Effects of Wearing Different Surgical Face Masks during High-Intensity, Short-Rest Resistance Exercise on Cardiorespiratory and Pulmonary Function and Perceptual Responses in Weightlifters
Acute Exercise Intensity and Memory Function: Evaluation of the Transient Hypofrontality Hypothesis
Acute high-intensity and moderate-intensity interval exercise do not change corticospinal excitability in low fit, young adults
Acute high-intensity exercise with low energy expenditure reduced LDL-c and total cholesterol in men
Acute high-intensity exercise-induced cognitive enhancement and brain-derived neurotrophic factor in young, healthy adults
Acute High-Intensity Intermittent Aerobic Exercise Reduces Plasma Angiopoietin-Like 2 in Patients With Coronary Artery Disease
Acute high-intensity interval exercise induces greater levels of serum brain-derived neurotrophic factor in obese individuals
Acute High-Intensity Interval Exercise-Induced Redox Signaling Is Associated with Enhanced Insulin Sensitivity in Obese Middle-Aged Men
Acute high-intensity interval training improves Tvent and peak power output in highly trained males
Acute HIIE elicits similar changes in human skeletal muscle mitochondrial H2O2 release, respiration, and cell signaling as endurance exercise even with less work
Acute intraocular pressure responses to high-intensity interval-training protocols in men and women
Acute Low-Volume High-Intensity Interval Exercise and Continuous Moderate-Intensity Exercise Elicit a Similar Improvement in 24-h Glycemic Control in Overweight and Obese Adults
Acute Metabolic and Chronic Hypertrophic Skeletal Muscle Responses to Low-Volume High-Intensity Resistance Exercise in Humans
Acute Modification of Cardiac Autonomic Function of High-Intensity Interval Training in Collegiate Male Soccer Players with Different Chronotype: A Cross-Over Study
Acute molecular responses to concurrent resistance and high-intensity interval exercise in untrained skeletal muscle
Acute Partial Sleep Deprivation And High-intensity Exercise Effects On Cardiovascular Autonomic Regulation And Lipemia Network: 442
Acute partial sleep deprivation and high-intensity interval exercise effects on postprandial endothelial function
Acute Perceptive Responses to 2 Combined Training Methods in Adolescents: A Crossover Study
Acute Physiological and Performance Responses to High-Intensity Resistance Circuit Training in Hypoxic and Normoxic Conditions
Acute Physiological Responses to High-Intensity Resistance Circuit Training vs. Traditional Strength Training in Soccer Players
Acute Physiological Responses To High-intensity Resistance Exercise With Blood Flow Restriction: 1103
Acute responses of platelet count and ADP-induced platelet aggregation to different high intensity interval exercise modes
Acute Responses to Resistance and High-Intensity Interval Training in Early Adolescents
Acute tendon changes in intense CrossFit workout: an observational cohort study
Adaptations Associated With an After-School Strength and Conditioning Program in Middle-School-Aged Boys: A Quasi-Experimental Design
Adaptations in mechanical muscle function, muscle morphology, and aerobic power to high-intensity endurance training combined with either traditional or power strength training in older adults: a rando[illegible]
Additional cardiovascular fitness when progressing from moderate- to high-intensity exercise training in previously trained breast cancer survivors
Adrenal cortical responses to high-intensity, short rest, resistance exercise in men and women
Advances for high-intensity interval training prescription in obesity
Aerobic and Anaerobic Fitness according to High-Intensity Interval Training Frequency in Youth Soccer Players in the Last Stage of Rehabilitation
Aerobic and resistance exercise training reverses age-dependent decline in NAD+ salvage capacity in human skeletal muscle
Affective and Enjoyment Responses to High-Intensity Interval Training in Overweight-to-Obese and Insufficiently Active Adults
Affective and perceptual responses during reduced-exertion high-intensity interval training (REHIT)
The affective interval: An investigation of the peaks and valleys during high- and moderate-intensity interval exercise in regular exercisers
Affective Responses during High-Intensity Interval Exercise Compared with Moderate-Intensity Continuous Exercise in Inactive Women
Affective Responses to Repeated Endurance Training Sessions with Different Intensities: A Randomized Trial
Age differences in human skeletal muscle fatigue during high-intensity intermittent exercise
Air pollution and high-intensity interval exercise: Implications to anti-inflammatory balance, metabolome and cardiovascular responses
Airflow restriction mask induces greater central fatigue after a non-exhaustive high-intensity interval exercise
Altering the rest interval during high-intensity interval training does not affect muscle or performance adaptations
Ambulatory heart rate variability in overweight and obese men after high-intensity interval exercise versus moderate-intensity continuous exercise
Anaerobic threshold is significantly enhanced by high intensity interval training (HIIT) in balinese athletic students
Analysis of body-composition techniques and models for detecting change in soft tissue with strength training
Ankle force and rate of force production increase following high intensity strength training in frail older adults
Appetite, energy intake and food reward responses to an acute High Intensity Interval Exercise in adolescents with obesity
Application of High-Intensity Functional Resistance Training in a Skilled Nursing Facility: An Implementation Study
Applying the critical velocity model for an off-season interval training program
Aquatic myofascial release applied after high intensity exercise increases flexibility and decreases pain
Are musculoskeletal benefits of 8 months high-intensity exercise maintained in postmenopausal women with low bone mass? LIFTMOR trial follow-up
Assessing the effectiveness of High Intensity Interval Training (HIIT) for smoking cessation in women: HIIT to quit study protocol
Assessing The Feasibility And Characterizing The Energy Expenditure And Relative Intensity Of A High-intensity Functional Training Regimen For Obese Female Adults: A Feasibility Study: 916
Assessment of prescribed increases in physical activity: application of a new method for microprocessor analysis of heart rate
Association Between Changes in Serum and Skeletal Muscle Metabolomics Profile With Maximum Power Output Gains in Response to Different Aerobic Training Programs: The Times Study
Association of serum BDNF concentration with high-intensity interval training
Association of skeletal muscle and serum metabolites with maximum power output gains in response to continuous endurance or high-intensity interval training programs: The TIMES study - A randomized
Astaxanthin Supplemented with High-Intensity Functional Training Decreases Adipokines Levels and Cardiovascular Risk Factors in Men with Obesity
Asymmetry inter-limb and performance in amateur athletes involved in high intensity functional training
Avaliação da força, potência e temperatura corporal em uma sessão de crossfit. / Assessment of body strength, power and temperature in a crossfit session
Battle between high-intensity interval training and moderate-intensity continuous training in cardiac rehabilitation practice
Beneficial Effects Of Supra High-Intensity Interval Training In Police Officers: 918
Beneficial Molecular Adaptations In BRCA-Mutation Carriers By Combined HIT/HIRT Intervention: Results From A Pilot Study
The Benefits of High-Intensity Interval Training on Cognition and Blood Pressure in Older Adults With Hypertension and Subjective Cognitive Decline: Results From the Heart & Mind Study
Benefits of selected physical exercise programs in detention: a randomized controlled study
Benefits on Hematological and Biochemical Parameters of a High-Intensity Interval Training Program for a Half-Marathon in Recreational Middle-Aged Women Runners
Beta-alanine supplementation and high-intensity interval training augments metabolic adaptations and endurance performance in college-aged men
Beta-endorphin response to high intensity exercise and music in college-age women
Biochemical responses and physical performance during high-intensity resistance circuit training in hypoxia and normoxia
Biological Response of Irisin Induced by Different Types of Exercise in Obese Subjects: A Non-Inferiority Controlled Randomized Study
Blood glucose turnover during high- and low-intensity exercise
Blood Lactate Concentration Is Not Related to the Increase in Cardiorespiratory Fitness Induced by High Intensity Interval Training
Blood Pressure and Heart Rate Variability Responses to High-Intensity Interval Training in Untrained Postmenopausal Women
Body Composition And Anaerobic Capacity In High Intensity Functional Training Athletes
Body composition and insulin sensitivity after high-intensity interval training in overweight/obese patients
Body Composition Changes in Response to Moderate- or High-Intensity Exercise Among Older Adults With or Without HIV Infection
Body weight multicomponent program improves power and functional capacity responses in older adults: a quasi-experimental study
The Bone Endocrine Function, Insulin Resistance Index, and Lipid Profile of Inactive Girls after 4 Weeks of High-Intensity Plyometric Training
Bone: High-intensity exercise to prevent fractures - risk or gain?
Brazilian jiu-jitsu specific training model highly emulates simulated match demands
Brief Exercise at Work (BE@Work): A Mixed-Methods Pilot Trial of a Workplace High-Intensity Interval Training Intervention
Brief Exercise Counseling and High-Intensity Interval Training on Physical Activity Adherence and Cardiometabolic Health in Individuals at Risk of Type 2 Diabetes: Protocol for a Randomized Controlled Tri[illegible]
Burpee Interval Training Is Associated With a More Favorable Affective Valence and Psychological Response Than Traditional High Intensity Exercise
Calculation of aerobic contribution during high intensity exercise
A call for universal criteria of high-intensity interval training in cardiac rehabilitation
CAMBIOS EN LA POTENCIA DEL SALTO VERTICAL POSTERIOR A ENTRENAMIENTO HIIT EN BASQUETBOLISTAS AMATEUR. / CHANGES IN THE POWER OF THE VERTICAL JUMP POSTERIOR TO HIIT TRAINING[illegible]
Can exercise improve reduced right ventricle function in obese children?
Can Gaming Get You Fit?
Can short-term high-intensity intermittent training reduce adiposity?
Can we identify who will adhere to long-term physical activity? Signal detection methodology as a potential aid to clinical decision making
Carbohydrate ingestion during prolonged high-intensity intermittent exercise: impact on affect and perceived exertion
Cardiac biomarkers response to high-intensity interval exercise in collegiate basketball players
Cardiac remodeling after six weeks of high-intensity interval training to exhaustion in endurance-trained men
The cardiac troponin response during high-intensity interval training
Cardio-Hypothalamic-Pituitary Coupling during Rest and in Response to Exercise
Cardiometabolic risk markers, adipocyte fatty acid binding protein (aFABP) and the impact of high-intensity interval training (HIIT) in obese adolescents
Cardiopulmonary and metabolic markers following a 6-week high-intensity interval training and moderate-intensity continuous training intervention in moderately trained individuals
Cardiopulmonary Demand of 16-kg Kettlebell Snatches in Simulated Girevoy Sport
Cardiorespiratory and perceptual responses of two interval training and a continuous training protocol in healthy young men
Cardiorespiratory fitness and accelerometer-determined physical activity following one year of free-living high-intensity interval training and moderate-intensity continuous training: a randomized trial
Cardiorespiratory Fitness and Body Composition Responses to Different Intensities and Frequencies of Exercise Training in Colorectal Cancer Survivors
Cardiorespiratory fitness modulates the proportions of monocytes and T helper subsets in lean and obese men
Cardiovascular endocrinology: High-intensity interval training benefits patients with T2DM
Cardiovascular fitness and energy expenditure response during a combined aerobic and circuit weight training protocol
Cardiovascular perturbations in high-intensity exercise. Can you exercise too hard?
Cardiovascular response to interval and continuous training in women
Catecholamine responses to short term high intensity resistance exercise overtraining (Modifications des catecholamines suite a un exercice de resistance bref et de haute intensite)
Central and peripheral adjustments during high-intensity exercise following cold water immersion
Central and Peripheral Oxygen Distribution in Two Different Modes of Interval Training
Cerebral Hemodynamic and Neurotrophic Factor Responses Are Dependent on the Type of Exercise
Cerebral oxygenation during cortical activation: the differential influence of three exercise training modalities. A randomized controlled trial
Cerebrovascular Regulation In Response To High-intensity Interval Exercise: 2422
Cerebrovascular response to an acute bout of low-volume high-intensity interval exercise and recovery in young healthy adults
Change in maximal fat oxidation in response to different regimes of periodized high-intensity interval training (HIIT)
Changes in anaerobic capacity following high-intensity interval training in females. (Abstract)
Changes in BNP and cardiac troponin I after high-intensity interval and endurance exercise in heart failure patients and healthy controls
Changes in Body Composition and Strength after 12 Weeks of High-Intensity Functional Training with Two Different Loads in Physically Active Men and Women: A Randomized Controlled Study
Changes in cardiorespiratory fitness and coronary heart disease risk factors following 24 wk of moderate- or high-intensity exercise of equal energy cost
Changes in Fat Oxidation and Body Composition after Combined Exercise Intervention in Sedentary Obese Chinese Adults
Changes in inflammation markers after a 10-week high-intensity combined strength and endurance training block in women: The effect of hormonal contraceptive use
Changes in muscle activation and conduction velocity during sustained contractions following endurance and high-intensity interval training
Changes in muscle quality following 12 weeks of power training in mobility-limited older adults: a randomized controlled trial
Changes in myosin heavy chain composition with heavy resistance training in 60- to 75-year-old men and women
Changes in Physical Fitness After 12 Weeks of Structured Concurrent Exercise Training, High Intensity Interval Training, or Whole-Body Electromyostimulation Training in Sedentary Middle-Aged Adults: A R[illegible]
Changes in plasma FFA concentrations before and after reduction in high intensity exercise
Changes in spinal bone density, back muscle size, and visceral adipose tissue and their interaction following a multi-component exercise program in older men: secondary analysis of an 18-month randomi[illegible]
Changes in the heart rate recovery to endurance effort after high intensity interval, strength, and concurrent exercise training in patients with insulin resistance
Changes in the timed finger-to-nose task performance following exercise of different intensities
The changes of some total antioxidant capacity and lipid peroxidation markers following the combined with high intensity interval training in sedentary postmenopausal women
Children's Enjoyment, Perceived Competency, and Vigorous Physical Activity During High-Intensity Interval Training in Physical Education
Chlorella vulgaris in combination with high intensity interval training in overweight and obese women: a randomized double-blind clinical trial
Circulating inflammatory biomarkers are not improved with combined low-volume high-intensity interval training and progressive resistance training in prediabetes
Cognitive Impairment during High-Intensity Exercise: Influence of Cerebral Blood Flow
Combinatory effects of high-intensity-strength training and sensorimotor training on muscle strength
Combined effect of eight weeks high intensity resistance training with ginger supplementation on waist to hip ratio, body composition and body mass in obese women
The combined effect of high-intensity intermittent training and vitamin D supplementation on glycemic control in overweight and obese adults
Combined effects of very short "all out" efforts during sprint and resistance training on physical and physiological adaptations after 2 weeks of training
Combined high-intensity interval training and green tea supplementation enhance metabolic and antioxidant status in response to acute exercise in overweight women
Combined high-intensity interval training as an obesity-management strategy for adolescents
Combined Small-Sided Game and High-Intensity Interval Training in Soccer Players: The Effect of Exercise Order
Combined strength and endurance training improves health-related quality of life in healthy middle-aged and older adults
Combined Training Reduces Subclinical Inflammation in Obese Middle-Age Men
COMBINING DIFFERENT CONCURRENT TRAINING METHODS IN OLDER ADULTS WITH METABOLIC SYNDROME
Comparable Effects of High-Intensity Interval Training and Prolonged Continuous Exercise Training on Abdominal Visceral Fat Reduction in Obese Young Women
The Comparative Effects Of Periodized Lower Body Strength Training Versus High Intensity Circuit Programming In Experienced Runners
Comparing Simple Insulin Sensitivity Indices to the Oral Minimal Model Postexercise
Comparing the effects of 8-weeks of high intensity interval trainings (HIIT), endurance and resistance training on serum miRNA-210 expression and HIF-1α concentration in young male athletes
Comparing the reinforcing value of high intensity interval training versus moderate intensity aerobic exercise in sedentary adults
Comparison between a high intensity interval training program with one resistance training to improvement of glycemic control of obese adults with insulin resistance
Comparison of Acute Cardiometabolic Responses in a 7-Minute Body Weight Circuit to 7-Minute HIIT Training Protocol
Comparison of bioelectric impedance and near infrared interactance for human body composition following either high intensity resistance or endurance training
Comparison of Cardiorespiratory and Metabolic Responses in Kettlebell High-Intensity Interval Training Versus Sprint Interval Cycling
Comparison of cerebrovascular reactivity recovery following high-intensity interval training and moderate-intensity continuous training
Comparison of Different Recovery Strategies After High-Intensity Functional Training: A Crossover Randomized Controlled Trial
Comparison of energy-matched high-intensity interval and moderate-intensity continuous exercise sessions on latency to eat, energy intake, and appetite
A comparison of growth hormone and cortisol responses to high intensity intermittent exercise between two different training periods
A Comparison Of High-intensity Interval Running And Tabata On Post-exercise Metabolism: A Pilot Analysis
Comparison of High-Intensity Resistance and Power Training Programs in Pre-Frail and Frail Older Adults
Comparison of HIIT and HIPT on Oxygen consumption
A comparison of low and high intensity exercise strategies on heat acclimatisation. (Abstract)
A comparison of low volume 'high-intensity-training' and high volume traditional resistance training methods on muscular performance, body composition, and subjective assessments of training
Comparison of performance and health indicators between perimenopausal and postmenopausal obese women: the effect of high-intensity interval training (HIIT)
Comparison of Psychological and Physiological Responses to Imposed vs. Self-selected High-Intensity Interval Training
Comparison of Strength-Training Adaptations in Early and Older Menopausal Women
Comparison of the acute effects of high-intensity interval training and continuous aerobic walking on inhibitory control
Comparison of the Affective Responses to Continuous Training and High-Intensity Interval Training Protocols: Application of the Dual-Mode Model
Comparison of the Effectiveness of High-Intensity Interval Training in Hypoxia and Normoxia in Healthy Male Volunteers: A Pilot Study
A comparison of the effects of reduced-exertion high-intensity interval training and continuous moderate-intensity training in obese young adults women
Comparison Of The Effects Of Tai Chi And High-intensity Interval Training On Bone Mineral Density In Substance Abuse: 450
Comparison of the Serum Metabolic Fingerprint of Different Exercise Modes in Men with and without Metabolic Syndrome
Comparison of Three Popular Exercise Modalities on V˙O2max in Overweight and Obese
Comparison of Three-Month HIIT and CMT Effects on Left Ventricle Echocardiography Observations in Male Employees
Comparison of two different resistance training intensities on excess post-exercise oxygen consumption in African American women who are overweight
Comparison of Ultrashort Versus Short High-Intensity Interval Training for Body Composition, Anaerobic, and Aerobic Performance: 680 May 30 4:30 PM - 4:45 PM
Comparison of vascular and hemodynamic responses between a continuous exercise session of moderate intensity and high intensity interval exercise in normotensive individuals
Compatibility of high-intensity strength and endurance training on hormonal and skeletal muscle adaptations
The compensatory effect of exercise on physical activity and energy intake in young men with overweight: The EFECT randomised controlled trial
Concomitant external pneumatic compression treatment with consecutive days of high intensity interval training reduces markers of proteolysis
Concomitant high-intensity interval training but not resistance exercise training improves muscle protein synthesis following six weeks very low-calorie diet among healthy men with overweight or obesity
Concurrent cardiovascular and resistance training in healthy older adults
Concurrent Exercise Training: Long-Term Changes in Body Composition and Motives for Continued Participation in Women with Obesity
Concurrent Training and Detraining: The Influence of Different Aerobic Intensities
Concurrent Training in Rugby Sevens: Effects of High-Intensity Interval Exercises
CONDICIONAMENTO FÍSICO PREVENTIVO E SAÚDE DE PRATICANTES ADULTOS DE CROSSFIT. / Preventive fitness and health of adults CrossFit practitioners
Continuous and high-intensity interval training: which promotes higher pleasure?
Continuous exercise but not high intensity interval training improves fat distribution in overweight adults
Continuous Moderate-Intensity but Not High-Intensity Interval Training Improves Immune Function Biomarkers in Healthy Young Men
Continuous One-Arm Kettlebell Swing Training on Physiological Parameters in US Air Force Personnel: A Pilot Study
Contrasting effects of a mixed-methods high-intensity interval training intervention in girl football players
Creatine Monohydrate Supplementation Does Not Augment Fitness, Performance, or Body Composition Adaptations in Response to Four Weeks of High-Intensity Interval Training in Young Females
Creatine supplementation and dynamic high-intensity intermittent exercise
CrossTalk opposing view: High intensity interval training does not have a role in risk reduction or treatment of disease
CrossTalk proposal: High intensity interval training does have a role in risk reduction or treatment of disease
The curse of inactivity: failure of acute exercise to enhance feeling states in a community sample of sedentary adults
Day-to-Day Changes in Muscle Protein Synthesis in Recovery From Resistance, Aerobic, and High-Intensity Interval Exercise in Older Men
Decreased insulin-stimulated brown adipose tissue glucose uptake after short-term exercise training in healthy middle-aged men
Deleterious effects of short-term, high-intensity exercise on immune function: evidence from leucocyte mitochondrial alterations and apoptosis
DESEMPENHO AERÓBIO DE MESATENISTAS INICIANTES SUBMETIDOS A TREINAMENTO INTERVALADO DE ALTA INTENSIDADE. / Aerobic performance of beginners table tennis players submitted to high inte[illegible]
Determinants of change in blood pressure during S.W.E.A.T.: the sedentary women exercise adherence trial
Determinants of PCr resynthesis following high-intensity exercise. (Abstract)
DEVELOPING A COMPREHENSIVE EXERCISE PRESCRIPTION: THE OPTIMAL ORDER FOR CARDIORESPIRATORY, RESISTANCE, FLEXIBILITY, AND NEUROMOTOR EXERCISE
The Difference Between Exercise-Induced Autonomic and Fitness Changes Measured After 12 Weeks and 20 Weeks of Medium-to-High Intensity Military Training: Erratum
The difference of maximum cardiorespiratory test on high-intensity interval training (HIIT) and aerobic
Differences in Carotid Artery Geometry and Flow Caused by Body Postural Changes and Physical Exercise
Different amount of training affects body composition and performance in High-Intensity Functional Training participants
Different consecutive training protocols to design an intervention program for overweight youth: a controlled study
Different exercise training modalities similarly improve heart rate variability in sedentary middle-aged adults: the FIT-AGEING randomized controlled trial
Differential effects of aerobic endurance, interval and resistance training on cellular senescence and inducible NO synthase
Differential Effects of Continuous and Interval Exercise Training on the Atherogenic Index of Plasma in the Non-Obese Young Male
Differential Effects of Exercise Programs on Neuregulin 4, Body Composition and Cardiometabolic Risk Factors in Men With Obesity
Differential effects of high-intensity interval training (HIIT) on choriocapillaris perfusion in healthy adults and patients with type 1 diabetes mellitus (T1DM)
Differential effects of intermittent energy restriction vs. continuous energy restriction combined high-intensity interval training on overweight/obese adults: A randomized controlled trial
Distracting factors involving the high-intensity interval training as modulator of retinal microvascular phenotype and DNA methylation of SHC1 gene
Diverse Exercises Similarly Reduce Older Adults' Mobility Limitations
Do No Harm: Is High Intensity Exercise Beneficial for Treatment of Neuromyelitis Optica?
Do physiological measures predict selected CrossFit(R) benchmark performance?
Do Short-Term Exercise Interventions Improve Cardiometabolic Risk Factors in Children?
Does a single session of high-intensity interval training provoke a transient elevated risk of falling in seniors and adults?
Does Becoming Fit Mean Feeling (f)it? A Comparison of Physiological and Experiential Fitness Data From the iReAct Study
Does exercise evoke neurological symptoms in healthy subjects?
Does inflammation markers or treatment type moderate exercise intensity effects on changes in muscle strength in cancer survivors participating in a 6-month combined resistance- and endurance exerci[illegible]
Does participation in a structured high-intensity exercise program influence daily physical activity patterns in older adults?
Does the cool-down content affect cortisol and testosterone production after a whole-body workout? A pilot study
Does time of day and short-duration high-intensity exercise effect haemostasis?
Dose-response effects of high-intensity interval neuromuscular exercise training on weight loss, performance, health and quality of life in inactive obese adults: Study rationale, design and methods of the [illegible]
Dose-response relationships between exercise intensity, cravings, and inhibitory control in methamphetamine dependence: An ERPs study
Dynamic Cerebral Autoregulation Is Maintained during High-Intensity Interval Exercise
Dynamic warm-up protocols, with and without a weighted vest, and fitness performance in high school female athletes
Early Motor Unit Conduction Velocity Changes to High-Intensity Interval Training versus Continuous Training
Efecto de un programa de entrenamiento interválico aeróbico de alta intensidad en población escolar femenina con sobrepeso u obesidad. / Effect of a high intensity aerobic interval training program on a[illegible]
Efecto del C-HIIT sobre control inhibitorio y comportamiento de jóvenes diagnosticados TDAH. / Effect of a C-HIIT program in the inhibitory control and behaviour of young ADHD
Efecto del entrenamiento interválico de alta intensidad sobre el comportamiento del sistema nervioso autónomo. / Effect of high intensity interval training on the autonomic nervous system
Efectos de la frecuencia de entrenamiento en circuito de alta intensidad sobre la fuerza isocinética y la composición corporal en sujetos no entrenados. / Effects of high intensity circuit training frequency[illegible]
Efectos de los juegos modificados en parámetros de la Condición física y antropométricos en escolares no deportistas. / Effects of modified games on physical condition and anthropometric parameters in [illegible]
Efectos del entrenamiento cardiovagal en la respuesta autonómica en personas con sobrepeso. / Effects of cardiovagal training on autonomic response in overweight people
EFEITOS FISIOLÓGICOS AGUDOS DE EXERCÍCIOS CONTÍNUO E INTERMITENTE EM SUJEITOS COM OBESIDADE. / Acute physiological effects of continuous and intermittent exercise in obese subjects
Effect of 3-week high-intensity interval training on VO2max, total haemoglobin mass, plasma and blood volume in well-trained athletes
Effect of 5 Years of Exercise Intervention at Different Intensities on Brain Structure in Older Adults from the General Population: A Generation 100 Substudy
Effect of 5 years of exercise training on the cardiovascular risk profile of older adults: the Generation 100 randomized trial
Effect of 6 weeks of very low-volume high-intensity interval training on oral glucose-stimulated incretin hormone response
Effect of 8 weeks of HIIT on hepatic enzyme levels, lipid profile and body composition in overweight young men
The effect of 12 weeks of combined upper- and lower-body high-intensity interval training on muscular and cardiorespiratory fitness in older adults
The Effect of 12 Weeks of Different Exercise Training Modalities or Nutritional Guidance on Cardiometabolic Risk Factors, Vascular Parameters, and Physical Fitness in Overweight Adults: Cardiometabolic[illegible]
Effect Of 12-weeks Of Moderate Versus High-intensity Interval Exercise Training On Postprandial Lipemia, Vascular Function And Arterial Stiffness After High-fat Meal Ingestion In Inactive Adults: 934 Boar[illegible]
[The effect of 24 weeks of moderate-to-high intensity strength training on the elderly]
Effect of a 12-Week Low vs. High Intensity Aerobic Exercise Training on Appetite-Regulating Hormones in Obese Adolescents: A Randomized Exercise Intervention Study
The effect of a combined high-intensity strength and speed training program on the running and jumping ability of soccer players
Effect of a field-based, plyometric protocol on cardiovascular, perceptual and performance responses during short sprinting in healthy active adults
Effect of a Four-Week Vegan Diet on Performance, Training Efficiency and Blood Biochemical Indices in CrossFit-Trained Participants
Effect of a game-based high intensity interval training program on body composition in primary education: comparison of the Sport Education model and traditional methodology
The effect of a high-intensity functional exercise program on activities of daily living: a randomized controlled trial in residential care facilities
Effect of a high-intensity functional exercise program on functional balance: preplanned subgroup analyses of a randomized controlled trial in residential care facilities
The Effect of a High-intensity Interval Training on Plasma Vitamin D Level in Obese Male Adolescents
The effect of a high-intensity interval training program on combat soldiers fitness
The effect of a lifestyle intervention on metabolic health in young women
Effect of a novel low volume, high intensity concurrent training regimen on recruit fitness and resilience
Effect of a short multicomponent exercise intervention focused on muscle power in frail and pre frail elderly: A pilot trial
The Effect of a Single Bout of High Intensity Intermittent Exercise on Glucose Tolerance in Non-diabetic Older Adults
Effect of a six-week high-intensity interval training on antioxidant capacity and lipid peroxidation in inactive men
The effect of a three month, low-load- high-repetitions group-based exercise program versus pilates on physical fitness and body composition in inactive women
The Effect of a Wingate Sprint with High-Intensity Circuit Weight Training on Growth Hormone Responses in Resistance Trained Young Males
Effect of adding high-intensity interval training to diet and carboxytherapy on metabolic cardiovascular risk factors in women with metabolic syndrome

Effect of Aerobic and Resistance Exercise on Cardiac Adipose Tissues: Secondary Analyses From a Randomized Clinical Trial	Christensen, Regitse Hojgaard; Wedell-Neergaard, Anne-Sophie; Lehrskov, Louise Lang; Legaard, Grit Elster; Dorph, Emma; [illegible]	[illegible]	2019		JAMA cardiology	4	8	778-787		https://dx.doi.org/10.1001/jamacardio.2019.2074	#2125
Effect of Aerobic Exercise Intensity on Energy Expenditure and Weight Loss in Severe Obesity-A Randomized Controlled Trial	Berge, Jarle; Hjelmesaeth, Joran; Hertel, Jens K.; Gjevestad, Espen; Smastuen, Milada Cvancarova; Johnson, Line Kristin; [illegible]	[illegible]	2021		Obesity (Silver Spring, Md.)	29	2	359-369		https://dx.doi.org/10.1002/oby.23078	#2128
Effect of aerobic exercise intensity on health-related quality of life in severe obesity: a randomized controlled trial	Berge, Jarle; Hjelmesaeth, Joran; Kolotkin, Ronette L.; Storen, Oyvind; Bratland-Sanda, Solfrid; Hertel, Jens Kristoffer; Gjevestad, [illegible]	[illegible]	2022		Health and quality of life outcomes	20	1	34		https://dx.doi.org/10.1186/s12955-022-01940-y	#2129
Effect of age and combined sprint and strength training on plasma catecholamine responses to a Wingate-test	Sellami, Maha; Abderrahman, Abderraouf Ben; Casazza, Gretchen A.; Kebsi, Wiem; Lemoine-Morel, Sophie; Bouguerra, Lotfi; [illegible]	[illegible]	2014		European Journal of Applied Physiology	114	5	969-82		https://dx.doi.org/10.1007/s00421-014-2828-7	#2138
THE EFFECT OF AGE ON PULMONARY AND MUSCLE OXYGENATION KINETICS FOLLOWING HIGH-INTENSITY INTERVAL TRAINING	Borges, Nattai R.; Reaburn, Peter R.; Doering, Thomas M.; Argus, Christos K.; Driller, Matthew W.	[illegible]	2017		Japanese Journal of Physical Fitness & Sports Medicine	66	1	73-73	122286844		#2139
The Effect of Age on the VO2max Response to High-Intensity Interval Training	Storen, Oyvind; Helgerud, Jan; Saebo, Mona; Stoa, Eva Maria; Bratland-Sanda, Solfrid; Unhjem, Runar J.; Hoff, Jan; Wang, [illegible]	[illegible]	2017		Medicine and science in sports and exercise	49	1	78-85			#2141
The Effect of All Extremity High Intensity Interval Training on Athero-Protective Factors and Endothelial Function in Overweight and Obese Women	Hovsepian, Volga; Marandi, Sayad M.; Esfarjani, Fahimeh; Zavar, Reihaneh; Sadeghi, Masoumeh	[illegible]	2021		International journal of preventive medicine	12		141		https://dx.doi.org/10.4103/ijpvm.IJPVM_248_19	#2145
The Effect of All-Extremity High-Intensity Interval Training on Plasma Pentraxin 3 in Young Overweight and Obese Women	Hovsepian, Volga; Marandi, Sayad Mohammad; Esfarjani, Fahimeh; Zavar, Reihaneh; Sadeghi, Masoumeh	[illegible]	2019		Asian Journal of Sports Medicine	10	4	1-7	141054493		#2147
The Effect of Angiotensin Converting Enzyme Genotype on Aerobic Capacity Following High Intensity Interval Training	Goddard, Nicole; Baker, Matthew; Higgins, T. I. M.; Cobbold, Christian	[illegible]	2014		International Journal of Exercise Science	7	3	250-259	102065320		#2162
The Effect of Beetroot Supplementation on High-Intensity Functional Training Performance	Collins, Sean M.; Kearns, Danielle	[illegible]	2020		International Journal of Exercise Science	13	2	667-676			#2172
Effect of block-periodized exercise training on bone and coronary heart disease risk factors in early post-menopausal women: a randomized controlled study	Kemmler, W.; Bebenek, M.; von Stengel, S.; Engelke, K.; Kalender, W. A.	[illegible]	2013		Scandinavian Journal of Medicine & Science in Sports	23	1	121-9		https://dx.doi.org/10.1111/j.1600-0838.2011.01335.x	#2181
Effect of Breaks in Prolonged Sitting or Low-Volume High-Intensity Interval Exercise on Markers of Metabolic Syndrome in Adults With Excess Body Fat: A Crossover Trial	Freire, Yuri Alberto; Macedo, Geovani de Araujo Dantas de; Browne, Rodrigo Alberto Vieira; Farias-Junior, Luiz Fernando; [illegible]	[illegible]	2019		Journal of Physical Activity & Health	16	9	727-735		https://dx.doi.org/10.1123/jpah.2018-0492	#2185
The effect of chronic progressive-dose sodium bicarbonate ingestion on CrossFit-like performance: A double-blind, randomized cross-over trial	Durkalec-Michalski, Krzysztof; Zawieja, Emilia E.; Podgorski, Tomasz; Loniewski, Igor; Zawieja, Bogna E.; Warzybok, Marta; [illegible]	[illegible]	2018		PloS one	13	5	e0197480		https://dx.doi.org/10.1371/journal.pone.0197480	#2198
The Effect of Circuit Resistance Training With Varying Intensity on Selected Inflammatory Markers in Obese Men	Emamdoost, S.; Daloii, A. A.; Barari, A.; Saeidi, A.	[illegible]	2021		Acta medica Iranica	59	12	733-739	CN-02387779	10.18502/acta.v59i12.8067	#2200
Effect of combined resistance and aerobic training on reactive hyperemia in men	Kawano, Hiroshi; Fujimoto, Kouhei; Higuchi, Mitsuru; Miyachi, Motohiko	[illegible]	2009		The journal of physiological sciences : JPS	59	6	457-64		https://dx.doi.org/10.1007/s12576-009-0057-3	#2208
The effect of combining HIIT and dry-land training on strength, technique, and 100-m butterfly swimming performance in age-group swimmers: a randomized controlled trial	Amara, Sofiene; Hammami, Raouf; Zacca, Rodrigo; Mota, Jorge; Negra, Yassine; Chortane, Sabri Gaied	[illegible]	2023		Biology of Sport	40	1	85-91		https://dx.doi.org/10.5114/biolsport.2023.110747	#2210
The Effect of Complex Training on Performance Indices	Chatzinikolaou, Athanasios; Kallinis, Konstantinos; Avloniti, Christina; Kampas, Antonis; Avloniti, Alexandra; Gourgoulis, Vassilis; [illegible]	[illegible]	2010		Inquiries in Sport & Physical Education	8	1	70-79	67763967		#2211
Effect of concurrent endurance and circuit resistance training sequence on muscular strength and power development	Chtara, Mokhtar; Chaouachi, Anis; Levin, Gregory T.; Chaouachi, Mustapha; Chamari, Karim; Amri, Mohamed; Laursen, Paul B.	[illegible]	2008		Journal of strength and conditioning research	22	4	1037-45		https://dx.doi.org/10.1519/JSC.0b013e31816a4419	#2216
Effect of Concurrent Training on Body Composition and Gut Microbiota in Postmenopausal Women with Overweight or Obesity	Dupuit, Marine; Rance, Melanie; Morel, Claire; Bouillon, Patrice; Boscaro, Audrey; Martin, Vincent; Vazeille, Emilie; Barnich, [illegible]	[illegible]	2022		Medicine and science in sports and exercise	54	3	517-529		https://dx.doi.org/10.1249/MSS.0000000000002809	#2218
Effect of concurrent training, flexible nonlinear periodization, and maximal-effort cycling on strength and power	McNamara, John M.; Stearne, David J.	[illegible]	2013		Journal of strength and conditioning research	27	6	1463-70		https://dx.doi.org/10.1519/JSC.0b013e318274f343	#2219

[illegible]

Effects of High-Intensity Interval Training vs. Sprint Interval Training on Anthropometric Measures and Cardiorespiratory Fitness in Healthy Young Women	Naves, Joao Pedro A.; Viana, Ricardo B.; Rebelo, Ana Cristina S.; de Lira, Claudio Andre B.; Pimentel, Gustavo D.; Lobo, Patricia Cristina B.; de Oliveira, Jordana C.; Ramirez-Campillo, Rodrigo; Gentil, Paulo
EFFECTS OF HIGH-INTENSITY INTERVAL TRAINING WITH DIFFERENT INTERVAL DURATIONS ON PHYSICAL PERFORMANCE IN HANDBALL PLAYERS	Rey, JorgeviaÑO-Santasmarinas; Ezequiel,; Carballeira, Sergio; PadrÓN-Cabo, Alexis
Effects of high-intensity interval training with hyperbaric oxygen	Alvarez Villela, Miguel; Dunworth, Sophia A.; Kraft, Bryan D.; Harlan, Nicole P.; Natoli, Michael J.; Suliman, Hagir B.; Moon, Richard E.
Effects of High-Intensity Interval Training With Specific Techniques on Jumping Ability and Change of Direction Speed in Karate Athletes: An Inter-individual Analysis	Ojeda-Aravena, Alex; Herrera-Valenzuela, Tomas; Valdes-Badilla, Pablo; Martin, Eduardo Baez-San; Cancino-Lopez, Jorge; Gallardo, Jairo Azocar; Zapata-Bastias, Jose; Garcia-Garcia, Jose Manuel
Effects of high-intensity interval training: Risk of bias by definition	Andreato, Leonardo Vidal
Effects of high-intensity intervals and moderate-intensity exercise on baroreceptor sensitivity and heart rate variability during recovery	Burma, Joel S.; Copeland, Paige V.; Macaulay, Alannah; Khatra, Omeet; Smirl, Jonathan D.
Effects of high-intensity multi-modal exercise training (HIT-MMEX) on bone mineral density and muscle performance in postmenopausal women. A Pilot randomized controlled trial	Riaz, Huma; Babur, Muhammad Naveed; Farooq, Ahsen
The Effects of High-Intensity Power Training versus Traditional Resistance Training on Exercise Performance	Chang, Yu-Hua; Chou, Yi-Chen; Chang, Yun-Chi; Tan, Kok-Hwa; Wu, Mei-Hsuan
Effects of high-intensity resistance circuit-based training in hypoxia on body composition and strength performance	Martinez-Guardado, Ismael; Ramos-Campo, Domingo J.; Olcina, Guillermo J.; Rubio-Arias, Jacobo A.; Chung, Linda H.; Marin-Cascales, Elena; Alcaraz, Pedro E.; Timon, Rafael
Effects of high-intensity resistance exercise on bone mineral density and muscle strength of 40-50-year-old women	Dornemann, T. M.; McMurray, R. G.; Renner, J. B.; Anderson, J. J.
Effects of high-intensity resistance training and low-intensity resistance training with vascular restriction on bone markers in older men	Karabulut, Murat; Bemben, Debra A.; Sherk, Vanessa D.; Anderson, Mark A.; Abe, Takashi; Bemben, Michael G.
Effects of high-intensity resistance training on bone mineral density in young male powerlifters	Tsuzuku, S.; Ikegami, Y.; Yabe, K.
Effects of High-Intensity Resistance Training on Circulating Levels of Irisin in Healthy Adults: A Randomized Controlled Trial	Fernandez-del-Valle, Maria; Short, Matthew J.; Chung, Eunhee; McComb, Jacalyn; Kloiber, Shelby; Naclerio, Fernando; Larumbe-Zabala, Eneko
Effects of high-intensity strength interval training program on body composition	JurÁNková, Michaela; BÍLÝ, JIŘÍ; HrazdÍRa, Eduard
Effects of high-intensity strength training on cardiovascular function	Hurley, B. F.; Seals, D. R.; Ehsani, A. A.; Cartier, L. J.; Dalsky, G. P.; Hagberg, J. M.; Holloszy, J. O.
Effects of high-intensity strength training on multiple risk factors for osteoporotic fractures. A randomized controlled trial	Nelson, M. E.; Fiatarone, M. A.; Morganti, C. M.; Trice, I.; Greenberg, R. A.; Evans, W. J.
Effects of high-intensity training on exercise performance of pre-adolescent female soccer players. (Abstract)	Grossner, C. M.; Johnson, E. M.; Cabrera, M. E.
Effects of high-intensity training on muscle lactate transporters and postexercise recovery of muscle lactate and hydrogen ions in women	Bishop, David; Edge, Johann; Thomas, Claire; Mercier, Jacques
Effects of High-Intensity Training on Physiological and Hormonal Adaptions in Well-Trained Cyclists	Sylta, Oystein; Tonnessen, Espen; Sandbakk, Oyvind; Hammarstrom, Daniel; Danielsen, Jorgen; Skovereng, Knut; Ronnestad, Bent R.; Seiler, Stephen
EFFECTS OF HIGH-INTENSITY TRAINING WITH ONE VERSUS THREE CHANGES OF DIRECTION ON YOUTH FEMALE BASKETBALL PLAYERS' PERFORMANCE	Sanchez-Sanchez, Javier; Carretero, Manuel; Ramirez-Campillo, Rodrigo; Petisco, Cristina; Diego, Maria; Gonzalo-Skok, Oliver; Nakamura, Fábio Y.
Effects of high-velocity circuit resistance and treadmill training on cardiometabolic risk, blood markers, and quality of life in older adults	Roberson, K. B.; Potiaumpai, M.; Widdowson, K.; Jaghab, A. M.; Chowdhari, S.; Armitage, C.; Seeley, A.; Jacobs, K. A.; Signorile, J. F.
Effects of HIIT and MIIT Suspension Training Programs on Sleep Quality and Fatigue in Older Adults: Randomized Controlled Clinical Trial	Jimenez-Garcia, Jose Daniel; Hita-Contreras, Fidel; de la Torre-Cruz, Manuel Jesus; Aibar-Almazan, Agustin; Achalandabaso-Ochoa, Alexander; Fabrega-Cuadros, Raquel; Martinez-Amat, Antonio
The effects of HIIT compared to MICT on endothelial function and hemodynamics in postmenopausal females	He, Hui; Wang, Chenghao; Chen, Xiaoke; Sun, Xinzheng; Wang, Yao; Yang, Jiani; Wang, Fatao
The effects of human sera conditioned by high-intensity exercise sessions and training on the tumorigenic potential of cancer cells	Baldelli, G.; De Santi, M.; Gervasi, M.; Annibalini, G.; Sisti, D.; Hojman, P.; Sestili, P.; Stocchi, V.; Barbieri, E.; Brandi, G.
Effects of in-season low-volume high-intensity plyometric training on explosive actions and endurance of young soccer players	Ramirez-Campillo, R.; Meylan, C.; Alvarez, C.; Henriquez-Olguin, C.; Martinez, C.; Cañas-Jamett, R.; Andrade, D. C.; Izquierdo, M.
Effects of indulgent food snacking, with and without exercise training, on body weight, fat mass, and cardiometabolic risk markers in overweight and obese men	Tucker, Wesley J.; Jarrett, Catherine L.; D'Lugos, Andrew C.; Angadi, Siddhartha S.; Gaesser, Glenn A.
Effects of intensity and amount of exercise on measures of insulin and glucose: Analysis of inter-individual variability	de Lannoy, Louise; Clarke, John; Stotz, Paula J.; Ross, Robert
Effects of Intermittent Hypoxia or Hyperoxia during High-Intensity Interval Training on Lymphocyte Mitochondrial Alteration	Wang, Ting Y.; Shih, Chun Y.; Lu, Kang H.; Lee, Mein M.; Chan, Kuei H.
[Effects of interval exercise in the improvement of glycemic control of obese adults with insulin resistance]	Dalmazzo, Verenna; Ponce, Alvaro; Delgado Floody, Pedro Antonio; Carrasco Alarcon, Vanessa Carola; Martinez Salazar, Cristian
Effects of Interval Training on Visceral Adipose Tissue in Centrally Obese 70-Year-Old Individuals: A Randomized Controlled Trial	Ballin, Marcel; Lundberg, Emmy; Sorlen, Niklas; Nordstrom, Peter; Hult, Andreas; Nordstrom, Anna
Effects of intrasession rest interval on strength recovery and reliability during high intensity exercise (Effets de periodes de repos sur la recuperation de la force et la fiabilite lors d'exercices de haute inter	Pincivero, D.; Lephart, S.; Karunakara, R.
Effects of L-citrulline supplementation on nitric oxide and antioxidant markers after high-intensity interval exercise in young men: a randomized controlled trial	Valaei, K.; Mehrabani, J.; Wong, A.
Effects of liquid carbohydrate ingestion on markers of anabolism following high-intensity resistance exercise	Thyfault, John P.; Carper, Michael J.; Richmond, Scott R.; Hulver, Matthew W.; Potteiger, Jeffrey A.
Effects of long or short duration stimulus during high-intensity interval training on physical performance, energy intake, and body composition	Alves, Elaine Dominguez; Salermo, Gabriela Pires; Panissa, Valeria Leme Goncalves; Franchini, Emerson; Takito, Monica Yuri
The effects of low intensity versus high intensity progressive resistance weight training on shoulder function in the elderly: a randomised controlled trial	Stavrinos, T. M.; Scarbek, Y.; Galambos, G.; Fiatarone-Singh, M. A.; Singh, N. A.
Effects Of Low Muscle Glycogen On High-intensity Exercise Performance: A Placebo-controlled Study: 756	Vigh-Larsen, Jeppe Foged; Ørtenblad, Niels; Andersen, Ole Emil; Overgaard, Kristian; Mohr, Magni
Effects of low- and high-intensity exercise training on body composition and substrate metabolism in obese adolescents	Lazzer, S.; Lafortuna, C.; Busti, C.; Galli, R.; Agosti, F.; Sartorio, A.
Effects of low- and high-intensity home-based exercise training on functional capacity in healthy middle-aged men	Gossard, D.; Haskell, W. L.; Taylor, C. B.; Mueller, J. K.; Rogers, F.; Chandler, M.; Ahn, D. K.; Miller, N. H.; DeBusk, R. F.
The effects of low-volume high-intensity interval training and circuit training on maximal oxygen uptake	Birkett, Stefan T.; Nichols, Simon; Sawrey, Richard; Gleadall-Siddall, Damien; McGregor, Gordon; Ingle, Lee
Effects of low-volume high-intensity interval training in a community setting: a pilot study	Reljic, Dejan; Wittmann, Felix; Fischer, Joachim E.
The effects of low-volume high-intensity interval versus moderate intensity continuous training on heart rate variability, and hemodynamic and echocardiography indices in men after coronary artery bypas	Ghardashi-Afousi, Alireza; Holisaz, Mohammad Taghi; Shirvani, Hossein; Pishgoo, Bahram
EFFECTS OF LOW-VOLUME HIGH-INTENSITY- VERSUS MODERATE-INTENSITY INTERVAL TRAINING ON CARDIOMETABOLIC HEALTH IN OBESE INDIVIDUALS: PRELIMINARY RESULTS OF A RANDOMIZED-CON	Reljic, D.; Frenk, F.; Herrmann, H. J.; Neurath, M. F.; Zopf, Y.
Effects of Low-Volume, High-Intensity Interval Training Compared with Continuous Training on Regional and Global Body Composition in Adults with Metabolic Syndrome: A post hoc Analysis of a Randomiz	Aristizabal, Juan Carlos; Montoya, Esperanza; Sanchez, Yeliana L.; Yepes-Calderon, Manuela; Narvaez-Sanchez, Raul; Gallo-Villegas, Jaime A.; Calderon, Juan C.
Effects of Low-Volume, High-Intensity Whole-Body Calisthenics on Army ROTC Cadets	Gist, Nicholas H.; Freese, Eric C.; Ryan, Terence E.; Cureton, Kirk J.

[illegible]

3

High-intensity interval training enhances mobilization/functionality of endothelial progenitor cells and depressed shedding of vascular endothelial cells undergoing hypoxia	Tsai, Hsing-Hua; Lin, Chin-Pu; Lin, Yi-Hui; Hsu, Chih-Chin; Wang, Jong-Shyan
High-intensity interval training every second week maintains VO2max in soccer players during off-season	Slettaløkken, Gunnar; Rønnestad, Bent R.
High-intensity interval training evokes larger serum BDNF levels compared with intense continuous exercise	Saucedo Marquez, Cinthia Maria; Vanaudenaerde, Bart; Troosters, Thierry; Wenderoth, Nicole
High-intensity interval training for health and fitness: can less be more?	Gaesser, Glenn A.; Angadi, Siddhartha S.
High-Intensity Interval Training for Improvement of Overhand Throwing Velocity	Maddigan, Meaghan E.; Behm, David G.; Belfry, Glen R.
High-Intensity Interval Training for Older People: No Pain and Lots of Gain	Dipietro, Loretta
High-Intensity Interval Training for Overweight Adolescents: Program Acceptance of a Media Supported Intervention and Changes in Body Composition	Herget, Sabine; Reichardt, Sandra; Grimm, Andrea; Petroff, David; Kapplinger, Jakob; Haase, Michael; Markert, Jana; Bluher, Susann
High-intensity interval training for undergraduate students: Study protocols for randomized controlled trials	Iyakrus,; Usra, Meirizal; Bayu, Wahyu Indra; Solahuddin, Soleh; Yusfi, Herri
High-intensity interval training has positive effects on performance in ice hockey players	Naimo, M. A.; de Souza, E. O.; Wilson, J. M.; Carpenter, A. L.; Gilchrist, P.; Lowery, R. P.; Averbuch, B.; White, T. M.; Joy, J.
High-intensity interval training improves acute plasma volume responses to exercise that is age dependent	Jabbour, Georges; Iancu, Horia-Daniel; Zouhal, Hassane; Mauriege, Pascale; Joanisse, Denis R.; Martin, Luc J.
High-Intensity Interval Training Improves Airway Health in Inactive Non-asthmatic Children	Rosenkranz, Sara K.; Rosenkranz, Richard R.; Hastmann, Tanis J.; Harms, Craig A.
High-intensity interval training improves cerebrovascular endothelial function and modulates P66SHC gene expression in cardiovascular risk patients: a randomized controlled trial	Lukas Streese, L.; Khan, A. W.; Deiseroth, A.; Hussain, S.; Suades, R.; Cosentino, F.; Hanssen, H.
High-Intensity Interval Training Improves Cognitive Flexibility in Older Adults	Mekari, Said; Neyedli, Heather F.; Fraser, Sarah; O'Brien, Myles W.; Martins, Ricardo; Evans, Kailey; Earle, Meghan; Aucoin, Rachelle; Chiekwe, Joy; Hollohan, Quinn; Kimmerly, Derek S.; Dupuy, Olivier
High-intensity interval training improves inflammatory and adipokine profiles in postmenopausal women with metabolic syndrome	Steckling, Flavia Mariel; Farinha, Juliano Boufleur; Figueiredo, Felipe da Cunha; Santos, Daniela Lopes Dos; Bresciani, Guilherme; Kretzmann, Nelson Alexandre; Stefanello, Silvio Terra; Courtes, Aline Alves; Beck [illegible]
High-Intensity Interval Training Improves Lipocalin-2 and Omentin-1 Levels in Men with Obesity	Atashak, Sirvan; Stannard, Stephen R.; Daraei, Ali; Soltani, Mohammad; Saeidi, Ayoub; Moradi, Fatah; Laher, Ismail; Hackney, Anthony C.; Zouhal, Hassane
High-Intensity Interval Training Improves Maximal Oxygen Consumption in Untrained Adult Asthmatics	Toennesen, L. L.; Meier, N.; Hostrup, M.; Porsbjerg, C.; Backer, V.
High-intensity interval training improves mitochondrial bioenergetics and suppresses thrombin generation in platelets undergoing hypoxic stress	Wang, J. S.
High-Intensity Interval Training Improves Mitochondrial Function and Suppresses Thrombin Generation in Platelets undergoing Hypoxic Stress	Wu, Li-Hua; Chang, Shao-Chiang; Fu, Tieh-Cheng; Huang, Ching-Hui; Wang, Jong-Shyan
High-intensity interval training improves specific performance in taekwondo athletes. / El entrenamiento intervalado de alta intensidad mejora el rendimiento específico en los atletas de taekwondo	Aravena Tapia, Diego Enrique; RomÁN Barrera, Valeria; da Silva Santos, Jonatas Ferreira; Franchini, Emerson; ValdÉS-Badilla, Pablo; Orihuela, Pedro; Herrera-Valenzuela, Tomás
High-intensity interval training in allogeneic adoptive T-cell immunotherapy - a big HIT?	Heinemann, Nele Carolin; Tischer-Zimmermann, Sabine; Wittke, Torge Christian; Eigendorf, Julian; Kerling, Arno; Framke, Theodor; Melk, Anette; Heuft, Hans-Gert; Blasczyk, Rainer; Maecker-Kolhoff, Britta; Eiz- [illegible]
High-intensity interval training in high-school physical education classes: Study protocol for a randomized controlled trial	Paulino da Silva Bento, Andre Filipe; Paez, Luis Carrasco; Manuel de Mendonca Raimundo, Armando
High-Intensity Interval Training in Patients with Pulmonary Embolism: A Randomized Controlled Trial	Ghram, Amine; Jenab, Yaser; Soori, Rahman; Choobineh, Siroos; Hosseinsabet, Ali; Niyazi, Sepideh; Shirani, Shapoor; Shafiee, Akbar; Jalali, Arash; Lavie, Carl J.; Wisloff, Ulrik
High-Intensity Interval Training in Prostate Cancer	Slomski, Anita
High-Intensity Interval Training in the Real World: Outcomes from a 12-Month Intervention in Overweight Adults	Roy, Melyssa; Williams, Sheila M.; Brown, Rachel C.; Meredith-Jones, Kim A.; Osborne, Hamish; Jospe, Michelle; Taylor, Rachael W.
High-Intensity Interval Training Increases Cardiac Output and ...O2max	Astorino, Todd A.; Edmunds, Ross M.; Clark, A. M. Y.; King, Leesa; Gallant, Rachael A.; Namm, Samantha; Fischer, Anthony; Wood, Kimi M.
High-Intensity Interval Training Increases Muscle GLUT4 and Mitochondrial Capacity in Prediabetes and Type 2 Diabetes	Little, Jonathan P.; Gillen, Jenna; Percival, Mike; Safdar, Adeel; Punthakee, Zubin; Tarnopolsky, Mark A.; Gibala, Martin J.
High-intensity interval training increases SIRT1 activity in human skeletal muscle	Gurd, Brendon J.; Perry, Christopher G. R.; Heigenhauser, George J. F.; Spriet, Lawrence L.; Bonen, Arend
High-intensity interval training is more effective than moderate-intensity continuous training in reducing abdominal fat mass in postmenopausal women with type 2 diabetes: A randomized crossover study	Maillard, F.; Rousset, S.; Pereira, B.; Boirie, Y.; Duclos, M.; Boisseau, N.
"High-intensity interval training may reduce in-stent restenosis following percutaneous coronary intervention with stent implantation: A randomized controlled trial evaluating the relationship to endotheli[illegible]	Meyer, Philippe; Gayda, Mathieu; Normandin, Eve; Guiraud, Thibaut; Juneau, Martin; Nigam, Anil
High-intensity interval training modulates male factor infertility through anti-inflammatory and antioxidative mechanisms in infertile men: A randomized controlled trial	Hajizadeh Maleki, Behzad; Tartibian, Bakhtyar
High-intensity interval training modulates retinal microvascular phenotype and DNA methylation of p66Shc gene: a randomized controlled trial (EXAMIN AGE)	Streese, Lukas; Khan, Abdul Waheed; Deiseroth, Arne; Hussain, Shafaat; Suades, Rosa; Tiaden, [illegible]
High-intensity interval training on body composition, functional capacity and biochemical markers in healthy young versus older people	Marzuca-Nassr, Gabriel Nasri; Artigas-Arias, Macarena; Olea, Maria Angelica; SanMartin-Calisto, [illegible]
High-Intensity Interval Training On Muscle Strength And Rate Of Torque Development In Female Lacrosse Players.: 63	Symons, T. Brock; Roberts, Alexandra H.; Carter, Kathleen A.; Caruso, John F.
High-intensity interval training or continuous training, combined or not with fasting, in obese or overweight women with cardiometabolic risk factors: study protocol for a randomised clinical trial	De Lorenzo, Andrea; Van Bavel, Diogo; de Moraes, Roger; Tibirica, Eduardo V.
High-intensity interval training reduces monocyte activation in obese adults	de Matos, Mariana Aguiar; Garcia, Bruna Caroline Chaves; Vieira, Denia Vargas; de Oliveira, Marc[illegible]
High-intensity interval training remodels the proteome and acetylome of human skeletal muscle	Hostrup, Morten; Lemminger, Anders Krogh; Stocks, Ben; Gonzalez-Franquesa, Alba; Larsen, J[illegible]
High-intensity interval training to promote cerebral oxygenation and affective valence during exercise in individuals with obesity	Marillier, Mathieu; Borowik, Anna; Chacaroun, Samarmar; Baillieul, Sebastien; Doutreleau, Stepha[illegible]
High-Intensity Interval Training versus Continuous Exercise: Is There a Difference Regarding the Magnitude of Blood Pressure Reduction?	Ferrari, Filipe; Martins, Vitor Magnus
High-intensity interval training versus continuous moderate intensity training: Effects on health outcomes and cardiometabolic disease risk factors in cancer survivors: A pilot study	Toohey, K.; Semple, S.; Pumpa, K.; Cooke, J.; Arnold, L.; Craft, P.; Yip, D.
High-Intensity Interval Training vs Moderate-Intensity Continuous Training for Women Undergoing Cardiovascular Rehabilitation	Van Iterson, Erik H.; Laffin, Luke J.; Cho, Leslie
High-intensity interval training vs. hydrochlorothiazide on blood pressure, cardiovascular health and cognition: Protocol of a non-inferiority trial	Langeard, Antoine; Cloutier, Simon-Olivier; Olmand, Miloudza; Saillant, Kathia; Gagnon, Christin[illegible]
High-intensity interval training vs. moderate interval training: the lack of equalisation, an ongoing problem	Andreato, L. V.; Coimbra, D. R.; de Carvalho, T.
High-intensity interval training vs. repeated-sprint training in tennis	Fernandez-Fernandez, Jaime; Zimek, Rico; Wiewelhove, Thimo; Ferrauti, Alexander
High-intensity interval training with or without chlorella vulgaris supplementation in obese and overweight women: effects on mitochondrial biogenesis, performance and body composition	Sanayei, Mahzad; Hajizadeh-Sharafabad, Fatemeh; Amirsasan, Ramin; Barzegar, Ali
High-intensity interval training-induced metabolic adaptation coupled with an increase in Hif-1alpha and glycolytic protein expression	Abe, Takaaki; Kitaoka, Yu; Kikuchi, Dale Manjiro; Takeda, Kohei; Numata, Osamu; Takemasa, To[illegible]
High-Intensity Interval Training, Appetite, and Reward Value of Food in the Obese	Martins, Catia; Aschehoug, Irina; Ludviksen, Marit; Holst, Jens; Finlayson, Graham; Wisloff, Ulrik [illegible]
High-intensity interval training: a simplified exercise programme in Phase 2 cardiac rehabilitation	Li, Wei-Guang; Wu, Yi-Xin; Liu, Pei-Liang
High-intensity interval training: a time-efficient strategy for health promotion?	Gibala, Martin J.
High-Intensity Interval Training: Applications for General Fitness Training	Schoenfeld, Brad; Dawes, Jay
High-intensity interval training: how much pain to get a gain?	Nassis, George P.
High-intensity interval training: Methodological caution to minimize risks of bias	Esteves, Joao Victor; Andreato, Leonardo Vidal
High-Intensity Interval Versus Continuous Moderateintensity Training in Adolescent Obesity Treatment	Wickham, E.; Evans, R.; Farthing, S.; Hollon, H.; Kang, L.; Bean, M.
High-intensity interval vs. moderate steady-state exercise	Ciolac, Emmanuel G.; Carvalho, Vitor O.; Guimaraes, Guilherme V.
High-Intensity Multimodal Training for Young People: It's Time to Think Inside the Box!	Gentil, Paulo; de Lira, Claudio Andre Barbosa; Vancini, Rodrigo Luiz; Ramirez-Campillo, Rodrigo [illegible]
High-Intensity Resistance and Impact Training Improves Bone Mineral Density and Physical Function in Postmenopausal Women With Osteopenia and Osteoporosis: The LIFTMOR Randomized Controlled T[illegible]	Watson, Steven L.; Weeks, Benjamin K.; Weis, Lisa J.; Harding, Amy T.; Horan, Sean A.; Beck, B[illegible]
High-intensity resistance exercise is not as effective as traditional high-intensity interval exercise for increasing the cardiorespiratory response and energy expenditure in recreationally active subjects	Jarvinen, Laura; Lundin Petersdotter, Sofi; Chaillou, Thomas
High-intensity resistance exercise with low repetitions maintains endothelial function	Morishima, Takuma; Tsuchiya, Yosuke; Iemitsu, Motoyuki; Ochi, Eisuke
High-Intensity Resistance Training Does Not Produce Immediate Ultrasonographic Changes in Muscle Tendons	Thygesen, Mathias Moller; Jordt, Ida; Kristensen, Malene Svane; Fisker, Filip Yang; Kildegaard, S[illegible]
High-intensity strength training improves quality of life in cancer survivors	De Backer, Ingrid C.; Van Breda, Eric; Vreugdenhil, Art; Nijziel, Marten R.; Kester, Arnold D.; Sche[illegible]
[High-intensity strength training of fragile elderly persons]	Kryger, A.; Bjerregaard, E.; Schroll, M.
High-intensity strength training to reduce risk factors for osteoporotic fractures	Macintyre, J.
High-Intensity Strength Training, Knee Pain, and Knee Joint Compressive Forces in Adults With Knee Osteoarthritis	Karaismailoglu, Bedri
High-intensity training elicits greater improvements in cardio-metabolic and reproductive outcomes than moderate-intensity training in women with polycystic ovary syndrome: a randomized clinical trial	Patten, Rhiannon K.; McIlvenna, Luke C.; Levinger, Itamar; Garnham, Andrew P.; Shorakae, Sou[illegible]
High-intensity training enhances executive function in children in a randomized, placebo-controlled trial	Moreau, David; Kirk, Ian J.; Waldie, Karen E.
High-intensity warm-ups: effects during subsequent intermittent exercise	Zois, James; Bishop, David; Aughey, Rob
High-intensity, high-volume exercise in addition to school exercise classes reduces endothelial progenitor cells, inflammation and catabolism in adolescent boys	Henriques, Magda; Fernandes, Raquel; Goncalves, Ana Cristina; Ribeiro, Ilda Patricia; Santos, Ru[illegible]
A high-intensity, intermittent exercise protocol and dynamic postural control in men and women	Whyte, Enda; Burke, Aoife; White, Elaine; Moran, Kieran
Higher Oxygen Uptake And Heart Rate Response In Recovery From Acute High Intensity Interval Exercise: 1124	Coe, Leah; Astorino, Todd
Higher-intensity exercise helps cancer survivors remain motivated	Martin, Eric; Battaglini, Claudio; Hands, Beth; Naumann, Fiona L.
Higher-Intensity Exercise Results in More Sustainable Improvements for VO2peak for Breast and Prostate Cancer Survivors	Martin, Eric A.; Battaglini, Claudio L.; Hands, Beth; Naumann, Fiona
HIIT enhances endurance performance and aerobic characteristics more than high-volume training in trained rowers	Ni Cheilleachair, Niamh J.; Harrison, Andrew J.; Warrington, Giles D.
HIIT moves out of the exercise lab and into the real world. A modified version of the High-Intensity Interval Training may be a time-saving, effective way for older, less fit adults to stay in shape	Anonymous,
HIIT produces increases in muscle power and free testosterone in male masters athletes	Herbert, P.; Hayes, L. D.; Sculthorpe, N. F.; Grace, F. M.
"HIIT the Inflammation": Comparative Effects of Low-Volume Interval Training and Resistance Exercises on Inflammatory Indices in Obese Metabolic Syndrome Patients Undergoing Caloric Restriction	Reljic, Dejan; Dieterich, Walburga; Herrmann, Hans J.; Neurath, Markus F.; Zopf, Yurdagul
HIIT the Road Jack: An Exploratory Study on the Effects of an Acute Bout of Cardiovascular High-Intensity Interval Training on Piano Learning	Swarbrick, Dana; Kiss, Alex; Trehub, Sandra; Tremblay, Luc; Alter, David; Chen, Joyce L.
HIITing it from all angles: assessing the feasibility of high-intensity interval training in schools, workplaces, and the community	Coombes, J.
Hippocampal structure, metabolism, and inflammatory response after a 6-week intense aerobic exercise in healthy young adults: a controlled trial	Wagner, Gerd; Herbsleb, Marco; de la Cruz, Feliberto; Schumann, Andy; Brunner, Franziska; Sch[illegible]
Hippocampal-Brainstem Connectivity Associated with Vagal Modulation after an Intense Exercise Intervention in Healthy Men	Bar, Karl-Jurgen; Herbsleb, Marco; Schumann, Andy; de la Cruz, Feliberto; Gabriel, Holger W.; W[illegible]
HORMETIC STRESS INTERVENTIONS AND MINDFULNESS EFFECTS ON COMPULSIVE EATING	Dileo, R.; Concepcion, C. J.; Fromer, E.; Guan, J.; Moore, J.; Zebarjadian, S.; Mason, A.; Prather [illegible]
How to exercise to increase lipolysis and insulin sensitivity: Fasting or following a single high-protein breakfast	Saghebjoo, Marziyeh; Kargar-Akbariyeh, Nasrin; Mohammadnia-Ahmadi, Mohsen; Saffari, Iman
Human hair follicle transcriptome profiling: a minimally invasive tool to assess molecular adaptations upon low-volume, high-intensity interval training	Zhang, Jing; Wallace, Sarah J.; Shiu, Maria Y.; Smith, Ingrid; Rhind, Shawn G.; Langlois, Valerie [illegible]
Human performance in high-intensity exercise with concentric and eccentric muscle contractions	Knuttgen, H. G.
IDENTIFICATION OF THE OPTIMAL HIIT PROTOCOL FOR FATIGUE RESISTANCE IN ADOLESCENT ATHLETES: A RANDOMIZED CONTROLLED TRIAL	Myong-Won, Seo; Jung-Min, Lee; Hyun Chul, Jung; Joon Young, Kim; Jong Kook, Song
IL-6 and IL-12 levels in response to honey supplementation combined with HIIT training in sedentary individuals	Tartibian, B.; Amanollahi, N.; Khayat, S. M. A.
IL-6, Antioxidant Capacity and Muscle Damage Markers Following High-Intensity Interval Training Protocols	Cipryan, Lukas
Immediate and sustained effects of intermittent exercise on inhibitory control and task-related heart rate variability in adolescents	Ludyga, Sebastian; Puhse, Uwe; Lucchi, Stefano; Marti, Jonas; Gerber, Markus
The impact of 2-years of high-intensity exercise training on left atrial function using high-resolution waveform analysis of pulmonary capillary wedge pressure pressure	Hieda, M.; Sarma, S.; Alad, N.; Kawalsky, J.; Hearon Christopher, M.; Erin, H.; Palmer, D.; Samel[illegible]
Impact of 4 weeks of interval training on resting metabolic rate, fitness, and health-related outcomes	Schubert, Matthew M.; Clarke, Holly E.; Seay, Rebekah F.; Spain, Katie K.
Impact of a 12-week high-intensity interval training without caloric restriction on body composition and lipid profile in sedentary healthy overweight/obese youth	Khammassi, Marwa; Ouerghi, Nejmeddine; Hadj-Taieb, Sameh; Feki, Moncef; Thivel, David; Bou[illegible]
Impact of a single bout of high-intensity interval exercise and short-term interval training on interleukin-6, FNDC5, and METRNL mRNA expression in human skeletal muscle	Eaton, Malcolm; Granata, Cesare; Barry, Julianne; Safdar, Adeel; Bishop, David; Little, Jonathan [illegible]
Impact of acute high-intensity interval exercise on plasma pentraxin 3 and endothelial function in obese individuals-a pilot study	Slusher, Aaron L.; Fico, Brandon G.; Dodge, Katelyn M.; Garten, Ryan S.; Ferrandi, Peter J.; Rod[illegible]
Impact of different exercise training modalities on energy and nutrient intake and food consumption in sedentary middle-aged adults: a randomised controlled trial	Jurado-Fasoli, L.; Amaro-Gahete, F. J.; De-la-O, A.; Castillo, M. J.
Impact of exercise intensity on body fatness and skeletal muscle metabolism	Tremblay, A.; Simoneau, J. A.; Bouchard, C.
THE IMPACT OF EXERCISE INTENSITY ON MOOD STATE OF PARTICIPANTS IN DANCE AEROBICS PROGRAMS	Rokka, Stella; Mavridis, George; Kouli, Olga
Impact of Exercise Intensity on Systemic Oxidative Stress, Inflammatory Responses, and Sirtuin Levels in Healthy Male Volunteers	Cho, Su-Youn; Chung, Young-Soo; Yoon, Hyoung-Ki; Roh, Hee-Tae
The impact of exercise intensity on whole body and adipose tissue metabolism during energy restriction in sedentary overweight men and postmenopausal women	Wahlin, Jean-Philippe; Dixon, Natalie C.; Betts, James A.; Thompson, Dylan
The Impact Of High Intensity Interval Training On Neurogenesis And Angiogenesis In The Dentate Gyrus: 2650	Lenhart, Darrin
The impact of high intensity interval training on serum chemerin, tumor necrosis factor-alpha and insulin resistance in overweight women	TaheriChadorneshin, H.; Cheragh-Birjandi, S.; Goodarzy, S.; Ahmadabadi, F.
Impact of High-Intensity Circuit Resistance Exercise on Physical Fitness, Inflammation, and Immune Cells in Female Breast Cancer Survivors: A Randomized Control Trial	Lee, Kwang-Jin; An, Keun-Ok
Impact of high-intensity exercise on energy expenditure, lipid oxidation and body fatness	Yoshioka, M.; Doucet, E.; St-Pierre, S.; Almeras, N.; Richard, D.; Labrie, A.; Despres, J. P.; Bouch[illegible]
Impact of high-intensity interval duration on perceived exertion	Kilpatrick, Marcus W.; Martinez, Nic; Little, Jonathan P.; Jung, Mary E.; Jones, Andrew M.; Price, [illegible]
The impact of high-intensity interval exercise training on NK-cell function and circulating myokines for breast cancer prevention among women at high risk for breast cancer	Coletta, Adriana M.; Agha, Nadia H.; Baker, Forrest L.; Niemiro, Grace M.; Mylabathula, Preteesh [illegible]
The impact of high-intensity interval training (HIIT) and moderate-intensity continuous training (MICT) on arterial stiffness and blood pressure in young obese women: a randomized controlled trial	de Oliveira, Gustavo H.; Boutouyrie, Pierre; Simoes, Caroline F.; Locatelli, Joao C.; Mendes, Victo[illegible]
Impact of high-intensity interval training combined with intermittent fasting on short-chain fatty acids in obese women	Battucci, G.; Almeida, O. G. G.; Solar, I.; De Martinis, E. C. P.; Cintra, D. E.; Freitas, E. C.
The impact of high-intensity interval training exercise on breast cancer survivors: a pilot study to explore fitness, cardiac regulation and biomarkers of the stress systems	Toohey, Kellie; Pumpa, Kate; McKune, Andrew; Cooke, Julie; Welvaert, Marijke; Northey, Joseph [illegible]
Impact of high-intensity interval training including Indigenous narratives on adolescents' mental health: a cluster-randomised controlled trial	Harris, Nigel; Warbrick, Isaac; Fleming, Theresa; Borotkanics, Robert; Atkins, Denise; Lubans, Da[illegible]
The Impact of High-Intensity Interval Training Versus Moderate-Intensity Continuous Training on Carotid Intima-Media Thickness and Ankle-Brachial Index in Middle-Aged Women	Farahati, Samaneh; Hosseini, Seyyed Reza Attarzadeh; Moazzami, Mahtab; Daloee, Mahdi Hasan[illegible]
The impact of high-intensity interval training versus moderate-intensity continuous training on endothelial progenitor cells in overweight women	Farahati, S.; Attarzadeh Hosseini, S. R.; Moazzami, M.; Hasanzadeh Daloee, M.; Hasanzadeh Dal[illegible]
Impact of High-Intensity Interval Training, Moderate-Intensity Continuous Training, and Resistance Training on Endothelial Function in Older Adults	O'Brien, Myles W.; Johns, Jarrett A.; Robinson, Susan A.; Bungay, Amanda; Mekary, Said; Kimm[illegible]
Impact of HIIT Sessions with and without Cognitive Load on Cortical Arousal, Accuracy and Perceived Exertion in Amateur Tennis Players	Clemente-Suarez, Vicente Javier; Villafaina, Santos; Garcia-Calvo, Tomas; Fuentes-Garcia, Juan P[illegible]
Impact of low-volume, high-intensity interval training on maximal aerobic capacity, health-related quality of life and motivation to exercise in ageing men	Knowles, Ann-Marie; Herbert, Peter; Easton, Chris; Sculthorpe, Nicholas; Grace, Fergal M.
IMPACT OF LOW, MEDIUM AND HIGH INTENSITY OF RESISTANCE TRAINING ON SOME SELECTED FITNESS VARIABLES AND MUSCULAR HYPERTROPHY AMONG UNDERWEIGHT MALES	Azeem, Kaukab
The Impact of Moderate or High-Intensity Combined Exercise on Systemic Inflammation Among Older Persons With and Without HIV	Erlandson, Kristine M.; Wilson, Melissa P.; MaWhinney, Samantha; Rapaport, Eric; Liu, Jay; Wils[illegible]
The Impact of Obesity on C1q/TNF-Related Protein-9 Expression and Endothelial Function following Acute High-Intensity Interval Exercise vs. Continuous Moderate-Intensity Exercise	Fico, Brandon G.; Garten, Ryan S.; Zourdos, Michael C.; Whitehurst, Michael; Ferrandi, Peter J.; [illegible]
The impact of resistance exercise on the cognitive function of the elderly	Cassilhas, Ricardo C.; Viana, Valter A. R.; Grassmann, Viviane; Santos, Ronaldo T.; Santos, Ruth [illegible]
Impact of resistance exercise order on postexercise hemodynamic measures in middle-aged and older women	Graca, A.; de Oliveira, D. V.; Ribeiro, A. S.; Lopes, W. A.; Fiorillo, R. G.; Fonseca, H. S.; Mayhew, [illegible]
Impact of training volume and intensity on RBC-NOS/NO pathway and endurance capacity	Koliamitra, Christina; Holtkamp, Bastian; Zimmer, Philipp; Bloch, Wilhelm; Grau, Marijke
The impact of variation and autonomy on psychological responses to high intensity interval training exercise	Mastrofini, Gianna F.; Collins, Robert P.; Rosado, Alanis P.; Tauran, Ralph C.; Fleming, Abby R.; [illegible]
The Impact of Vigorous-Intensity Aerobic Exercise Training on Lifestyle Behavior and Mental Health of Male Problem Gamblers	Okechukwu, Chidiebere Emmanuel
Implementation of High Intensity Interval Training and Autoregulatory Progressive Resistance Exercise in a Law Enforcement Training Academy	Martinez, Gabriel J.; Ma, Xin; Best, Stuart; Johnson, Benjamin F.; Abel, Mark G.
Improvements cardiometabolic risk factors in Latin American Amerindians (the Mapuche) with concurrent training	Alvarez, Cristian; Ramirez-Velez, Robinson; Ramirez-Campillo, Rodrigo; Lucia, Alejandro; Alonso-[illegible]
Improvements in cognition and associations with measures of aerobic fitness and muscular power following structured exercise	Cherup, N.; Roberson, K.; Potiaumpai, M.; Widdowson, K.; Jaghab, A. M.; Chowdhari, S.; Armi[illegible]
Improvements on Cardiovascular Diseases Risk Factors in Obese Adolescents: A Randomized Exercise Intervention Study	Silva, Humberto Jose; Andersen, Lars Bo; Lofrano-Prado, Mara Cristina; Barros, Mauro V.; Freita[illegible]
Improving health-related fitness in adolescents: the CrossFit Teens randomized controlled trial	Eather, N.; Morgan, P.; Lubans, D.
Improving Performance of Heavy Load Carriage During High-Intensity Combat-Related Tasks	Mala, Jesse; Szivak, Tunde K.; Kraemer, William J.
Improving the physical qualities of students in higher educational establishments of Ukraine on guard activity via circular training	Dmytro, Kyslenko; Yurii, Yukhno; Iryna, Zhukevych; Valentin, Bondarenko; Rostislav, Radzievski[illegible]
Increase in S6K1 phosphorylation in human skeletal muscle following resistance exercise occurs mainly in type II muscle fibers	Koopman, Rene; Zorenc, Antoine H. G.; Gransier, Rudy J. J.; Cameron-Smith, David; van Loon, [illegible]
Increased velocity at VO2max and load carriage performance in army ROTC cadets: prescription using the critical velocity concept	Dicks, Nathan D.; Mahoney, Sean J.; Kramer, Mark; Lyman, Katie J.; Christensen, Bryan K.; Pett[illegible]
Increases in motor unit action potential amplitudes are related to muscle hypertrophy following eight weeks of high-intensity exercise training in females	Jenkins, Nathaniel D. M.; Rogers, Emily M.; Banks, Nile F.; Muddle, Tyler W. D.; Colquhoun, Ry[illegible]
The independent and combined effects of 16 weeks of vigorous exercise and energy restriction on body mass and composition in free-living overweight men--a randomized controlled trial	Cox, Kay L.; Burke, Valerie; Morton, Alan R.; Beilin, Lawrie J.; Puddey, Ian B.
Individual Blood Lipid And Apolipoprotein Responses To A Single Bout Of High Intensity Functional Training: 1757	Smith, Leslie E.; Dages, Allison G.; Lewis, Nicole R.; Van Guilder, Gary P.; Harris, Nigel K.; Dalleck [illegible]
Individual cardiovascular responsiveness to work-matched exercise within the moderate- and severe-intensity domains	Maturana, Felipe Mattioni; Schellhorn, Philipp; Erz, Gunnar; Burgstahler, Christof; Widmann, Man[illegible]
Individual physiological and mitochondrial responses during 12 weeks of intensified exercise	Jacques, Macsue; Landen, Shanie; Alvarez Romero, Javier; Yan, Xu; Garnham, Andrew; Hiam, D[illegible]
Individual Variability in Waist Circumference and Body Weight in Response to Exercise	Hammond, Brittany P.; Stotz, Paula J.; Brennan, Andrea M.; Lamarche, Benoit; Day, Andrew G.; [illegible]
Individualized aerobic and high intensity training for asthmatic children in an exercise readaption program	Varray, A. L.; Mercier, J. G.; Terral, C. M.; Prefaut, C. G.
Inflammatory and Biochemical Biomarkers in Response to High Intensity Resistance Training in Trained and Untrained Men	Ashtary-Larky, Damoon; Lamuchi-Deli, Nasrin; Milajerdi, Alireza; Salehi, Mohsen Bakhtiar; Alipour[illegible]
Inflammatory cytokines and metabolic responses to high-intensity intermittent training: effect of the exercise intensity	Rhibi, Fatma; Zouhal, Hassane; Lira, Fabio Santos; Ouerghi, Nejmeddine; Prioux, Jacques; Besb[illegible]
The influence of a high intensity physical activity intervention on a selection of health related outcomes: an ecological approach	Buchan, Duncan S.; Ollis, Stewart; Thomas, Non E.; Baker, Julien S.
The Influence Of A Single Acute Bout Of High-intensity Interval Exercise On Cardiac Autonomic Modulation In Younger And Older Individuals With Good Cardiorespiratory Fitness: 2136	Torres, Ricardo; Lowry, Catherine; Adair, Kathleen; Ismaeel, Ahmed; Beeson, Cassidy; Koutakis, [illegible]
The influence of a specific high intensity circuit training during physical education classes in children's physical activity and body composition markers	Marinho, Daniel A.; Neiva, Henrique P.; Marques, Luis; Lopes, Vitor P.; Morais, Jorge E.
Influence of Acute and Chronic High-Intensity Intermittent Aerobic Plus Strength Exercise on BDNF, Lipid and Autonomic Parameters	Figueiredo, Caique; Antunes, Barbara M.; Giacon, Thais R.; Vanderlei, Luiz C. M.; Campos, Edu[illegible]
The Influence of Acute Sprint Interval Training on Cognitive Performance of Healthy Younger Adults	Herold, Fabian; Behrendt, Tom; Meisner, Caroline; Muller, Notger G.; Schega, Lutz
The influence of concurrent training intensity on serum irisin and abdominal fat in postmenopausal women	Rashti, Bita Azimi; Mehrabani, Javad; Damirchi, Arsalan; Babaei, Parvin
Influence of continuous and discontinuous training protocols on subcutaneous adipose tissue and plasma substrates. / Influence de protocoles d ' entrainement en continu et en discontinu sur les tissus ad[illegible]	Maeder, U.; Roth, P.; Furrer, R.; Brechet, J. P.; Boutellier, U.
Influence of continuous and interval training on oxygen uptake on-kinetics	Berger, Nicolas J. A.; Tolfrey, Keith; Williams, Alun G.; Jones, Andrew M.
The influence of exercise intensity and frequency on cardiorespiratory fitness and body composition in colorectal cancer survivors: a randomized controlled trial	Devin, J. L.; Jenkins, D. G.; Sax, A. T.; Hughes, G. I.; Aitken, J. F.; Chambers, S. K.; Dunn, J. C[illegible]
Influence of exercise intensity on abdominal fat and adiponectin in elderly adults	Coker, Robert H.; Williams, Rick H.; Kortebein, Patrick M.; Sullivan, Dennis H.; Evans, William J.
The influence of exercise intensity on fatigue in colorectal cancer survivors: a randomized controlled trial	Adlard, K. N.; Devin, J. L.; Jenkins, D. G.; Bolam, K. A.; Aitken, J. F.; Chambers, S. K.; Dunn, J. [illegible]
The influence of high-intensity compared with moderate-intensity exercise training on cardiorespiratory fitness and body composition in colorectal cancer survivors: a randomised controlled trial	Devin, James L.; Sax, Andrew T.; Hughes, Gareth I.; Jenkins, David G.; Aitken, Joanne F.; Cham[illegible]
Influence of high-intensity intermittent training on glycolipid metabolism in obese male college students	Sun, Jing; Cheng, Wenjuan; Fan, Zhengzheng; Zhang, Xuelin
The influence of high-intensity interval training and moderate-intensity continuous training on sedentary time in overweight and obese adults	Nugent, Sean F.; Jung, Mary E.; Bourne, Jessica E.; Loeppky, Jason; Arnold, Abigail; Little, Jona[illegible]
Influence of high-intensity interval training on ventilatory efficiency in trained athletes	Salazar-Martinez, Eduardo; Santalla, Alfredo; Orellana, Jose Naranjo; Strobl, Jochen; Burtscher, M[illegible]
Influence of high-intensity interval training to exhaustion on the directional sensitivity of the cerebral pressure-flow relationship in young endurance-trained men	Abbariki, Faezeh; Roy, Marc-Antoine; Labrecque, Lawrence; Drapeau, Audrey; Imhoff, Sarah; Sm[illegible]
Influence of personality and self-efficacy on perceptual responses during high-intensity interval exercise in adolescents	Malik, Adam A.; Williams, Craig A.; Weston, Kathryn L.; Barker, Alan R.
Influence of Recovery Postures on Thermoregulatory Responses following High-intensity Intermittent Exercise	Pinthong, Metta; Tikamram, Jatuporn
Influence of the ACTN3 Genotype and the Exercise Intensity on the Respiratory Exchange Ratio and Excess Oxygen Consumption After Exercise	de L Correa, Hugo; Ribeiro, Heitor S.; Maya, Athila T. D.; Neves, Rodrigo P.; de Moraes, Milton R[illegible]
Influence of training intensity on adaptations in acid/base transport proteins, muscle buffer capacity, and repeated-sprint ability in active men	McGinley, Cian; Bishop, David J.
Influence to high-intensity intermittent and moderate-intensity continuous exercise on indices of cardio-inflammatory health in men	Santos, Carolina C.; Diniz, Tiego A.; Inoue, Daniela S.; Gerosa-Neto, Jose; Panissa, Valeria L. G.; [illegible]
Influences of two high intensity interval exercise protocols on the main determinants of blood fluidity in overweight men	Ahmadizad, Sajad; Bassami, Minoo; Hadian, Mohsen; Eslami, Maryam
Influencia de la pliometría basada en un Entrenamiento Intervalado de Alta Intensidad sobre la altura de salto y pico de potencia en futbolistas Sub - 17. / The influence of High-Intensity Interval Training b[illegible]	Daniel Barahona-Fuentes, Guillermo; Huerta Ojeda, Álvaro; Galdames Maliqueo, Sergio
Insulin sensitivity and mitochondrial respiration following 12 weeks of high-intensity aerobic interval, resistance, or combined training in 18-to 30-year-old humans	Robinson, M. M.; Konopka, A. R.; Johnson, M. L.; Shankarappa, M.; Esponda, R. R.; Lanza, I. [illegible]
Integration of metabolomics and proteomics to reveal the metabolic characteristics of high-intensity interval training	Zhao, Jingjing; Wang, Yang; Zhao, Dan; Zhang, Lizhen; Chen, Peijie; Xu, Xin
Intensite d'entrainement et aptitude aerobie chez le sujet age. / High intensity training and aerobic fitness of trained aged subjects	Masse-Biron, J.; Mercier, J.; Adam, B.; Prefaut, C.
Intensity Dependent Effects of Interval Resistance Training on Myokines and Cardiovascular Risk Factors in Males With Obesity	Ataeinosrat, Ali; Saeidi, Ayoub; Abednatanzi, Hossein; Rahmani, Hiwa; Daloii, Asieh Abbassi; Pas[illegible]
Intensity Matters: High-intensity Interval Exercise Enhances Motor Cortex Plasticity More Than Moderate Exercise	Andrews, Sophie C.; Curtin, Dylan; Hawi, Ziarih; Wongtrakun, Jaeger; Stout, Julie C.; Coxon, Jan[illegible]
Intensity of resistance exercise determines adipokine and resting energy expenditure responses in overweight elderly individuals	Fatouros, Ioannis G.; Chatzinikolaou, Athanasios; Tournis, Symeon; Nikolaidis, Michalis G.; Jam[illegible]
Intensity-dependence of exercise and active recovery in high-intensity interval training	Kemi, Ole J.; Fowler, Ewan; McGlynn, Karen; Primrose, Deborah; Smithwaite, Rachel; Wilson, J[illegible]
Intensive lifestyle intervention improves cardiometabolic and exercise parameters in metabolically healthy obese and metabolically unhealthy obese individuals	Dalzill, Claudie; Nigam, Anil; Juneau, Martin; Guilbeault, Valerie; Latour, Elise; Mauriege, Pascale; [illegible]
Intensive lifestyle intervention including high-intensity interval training program improves insulin resistance and fasting plasma glucose in obese patients	Marquis-Gravel, Guillaume; Hayami, Douglas; Juneau, Martin; Nigam, Anil; Guilbeault, Valerie; La[illegible]
Inter- and Intra-Individual Analysis of Post-Exercise Hypotension Following a Single Bout of High-Intensity Interval Exercise and Continuous Exercise: A Pilot Study	Costa, E. C.; Dantas, T. C. B.; de Farias Junior, L. F.; Frazao, D. T.; Prestes, J.; Moreira, S. R.; R[illegible]
Interindividual responses to different exercise stimuli among insulin-resistant women	Alvarez, C.; Ramirez-Velez, R.; Ramirez-Campillo, R.; Ito, S.; Celis-Morales, C.; Garcia-Hermoso, [illegible]
Interindividual variability of adaptations following either traditional strength or power training combined to endurance training in older men: A secondary analysis of a randomized clinical trial	Formighieri, Carolina; Muller, Diana C.; Saez de Asteasu, Mikel L.; Mello, Alexandre; Teodoro, Ju[illegible]
Interleukin-6 and associated cytokine responses to an acute bout of high-intensity interval exercise: the effect of exercise intensity and volume	Cullen, Tom; Thomas, Andrew W.; Webb, Richard; Hughes, Michael G.
Internal versus external focus of attention on high-intensity exercise performance in judo athletes	Kons, Rafael Lima; Detanico, Daniele; Franchini, Emerson
The interplay between inflammatory markers and body composition with 6 months of HIIT in breast, colorectal and prostate cancer survivors	Farley, M. J.; Skinner, T. L.; Schaumberg, M. A.; Jenkins, D. G.
Interpretation of exercise-induced changes in human skeletal muscle mRNA expression depends on the timing of the post-exercise biopsies	Kuang, Jujiao; McGinley, Cian; Lee, Matthew J. C.; Saner, Nicholas J.; Garnham, Andrew; Bisho[illegible]
Interval training during concurrent training optimizes cardiorespiratory adaptations in women. / Exercício intervalado durante o treinamento concorrente otimiza as adaptações cardiorrespiratórias em mu[illegible]	Ferrari, Rodrigo; Lima Alberton, Cristine; Santana Pinto, Stephanie; Lusa Cadore, Eduardo; Silvei[illegible]
Interval Training with Different Intensities in Overweight/Obese Adolescent Females	Abassi, Wissal; Ouerghi, Nejmeddine; Nikolaidis, Pantelis T.; Hill, Lee; Racil, Ghazi; Knechtle, Bea[illegible]
Investigating changes in muscle occurring in association with high-intensity exercise	Hodgson, D. R.; Byrd, S. K.; McCutcheon, L. J.
Investigating the reproducibility of maximal oxygen uptake responses to high-intensity interval training	Del Giudice, Michael; Bonafiglia, Jacob T.; Islam, Hashim; Preobrazenski, Nicholas; Amato, Aless[illegible]
Investigation into the influence of food timing around exercise training and the effects on energy and metabolic health: is it better to eat before or after exercise?	Morencos, C. M.; Scott, G.; Collins, A.
The iReAct study â€" A biopsychosocial analysis of the individual response to physical activity	Thiel, A.; Sudeck, G.; Gropper, H.; Maturana, F. M.; Schubert, T.; Srismith, D.; Widmann, M.; Be[illegible]
Irisin in response to exercise in humans with and without metabolic syndrome	Huh, Joo Young; Siopi, Aikaterina; Mougios, Vassilis; Park, Kyung Hee; Mantzoros, Christos S.
Irisin response to acute moderate intensity exercise and high intensity interval training in youth of different obesity statuses: A randomized crossover trial	Colpitts, Benjamin H.; Rioux, Brittany V.; Eadie, Ashley L.; Brunt, Keith R.; Senechal, Martin
Is 2-week calisthenics high-intensity interval training enough to change aerobic and anaerobic capacity? / İki haftalık kalistenik yüksek şiddetli aralıklı egzersiz aerobik ve anaerobik kapasiteyi artırmaya [illegible]	Alsairawan, M. Abdullatif; GÜRpinar, Barış; İLÇİN, Nursen
Is a high-intensity exercise test better than a graded exercise test in eliciting exercise-related arrhythmias?	Sagray, Ezequiel; Allison, Thomas G.; Wackel, Philip L.
Is high-intensity interval training a time-efficient exercise strategy to improve health and fitness?	Gillen, Jenna B.; Gibala, Martin J.
Is Matching Exercise Intensity to Heart Rate Variability a Key to Effective Conditioning for Dancers? A Prospective Randomized Controlled Trial	Butulis, Meredith N.; Fedor, Brittany J.; McGaver, Rebecca Stone; Giveans, M. Russell
Is Oxygen Uptake Measurement Enough to Estimate Energy Expenditure During High-Intensity Intermittent Exercise? Quantification of Anaerobic Contribution by Different Methods	Panissa, Valeria L. G.; Fukuda, David H.; Caldeira, Renan S.; Gerosa-Neto, Jose; Lira, Fabio S.; Z[illegible]
Is Perceived Exertion a Useful Indicator of the Metabolic and Cardiovascular Responses to a Metabolic Conditioning Session of Functional Fitness?	Alsamir Tibana, Ramires; Manuel Frade de Sousa, Nuno; Prestes, Jonato; da Cunha Nascimento[illegible]
Is There a Dose-Response Relationship between High-Intensity Interval Exercise (HIIE) Intensity and Affective Valence? Analysis of Three HIIE Sessions Performed with Different Amplitudes	Silva, Michel Oliveira; Santos, Tony Meireles; Inoue, Allan; Santos, Lucas Eduardo Rodrigues; de[illegible]
Isokinetic force-power profile of the shoulder joint in males participating in CrossFit training and competing at different levels	Torres-Banduc, Maximiliano A.; Jerez-Mayorga, Daniel; Moran, Jason; Keogh, Justin W. L.; Ram[illegible]
KAATSU Training Improves Strength and mCSA of the Thigh Equivalent to High Intensity Resistance Training	Sherk, Kyle A.; Kim, Dae-Yeol; Singh, Harshvardhan; Sherk, Vanessa D.; Bemben, Debra A.; Bem[illegible]
KARADA VE SU İÇİNDE YAPILAN KISA DÖNEM YÜKSEK ŞİDDETLİ ARALIKLI EGZERSİZ EĞİTİMİNİN AEROBİK KAPASİTE VE SPİNAL STABİLİZASYON ÜZERİNE ETKİLERİNİN KARŞILAŞTIRILMASI / COMPARISON[illegible]	Altinkaya, Neyran; GÜRpinar, Barış; İLÇİN, Nursen
Kettlebell training for musculoskeletal and cardiovascular health: a randomized controlled trial	Jay, K.; Frisch, D.; Hansen, K.; Zebis, M. K.; Andersen, C. H.; Mortensen, O. S.; Andersen, L. L. [illegible]
Kinetics of VO(2) with very high intensity exercise	Hughson, R. L.
Lactate consumption mediates repeated high-intensity interval exercise-enhanced executive function in adult males	Cho, Hae-Sung; Lee, Won Sang; Yoon, Kyeong Jin; Park, Soo Hong; Shin, Hyung Eun; Kim, Ye[illegible]
Late Neutrophil Priming Following a Single Session of High-intensity Interval Exercise	de Oliveira Ottone, Vinicius; Costa, Karine Beatriz; Tossige-Gomes, Rosalina; de Matos, Mariana [illegible]
Left Atrial Electromechanical Remodeling Following 2 Years of High-Intensity Exercise Training in Sedentary Middle-Aged Adults	McNamara, David A.; Aiad, Norman; Howden, Erin; Hieda, Michinari; Link, Mark S.; Palmer, Dean[illegible]
Lifelong exercise, but not short-term high-intensity interval training, increases GDF11, a marker of successful aging: a preliminary investigation	Elliott, Bradley T.; Herbert, Peter; Sculthorpe, Nicholas; Grace, Fergal M.; Stratton, Daniel; Hayes, [illegible]
Loads and movement speed affect energy expenditure during circuit resistance exercise	Roberson, Kirk B.; Jacobs, Kevin A.; White, Morgan J.; Signorile, Joseph F.
Long Non-coding RNAs Are Differentially Expressed After Different Exercise Training Programs	Bonilauri, Bernardo; Dallagiovanna, Bruno
The Long-term Effect of Different Exercise Intensities on High-Density Lipoprotein Cholesterol in Older Men and Women Using the Per Protocol Approach: The Generation 100 Study	Berglund, Ida; Vesterbekkmo, Elisabeth Kleivhaug; Retterstol, Kjetil; Anderssen, Sigmund A.; Fia[illegible]
Long-term effects of aerobic training versus combined aerobic and resistance training in modifying cardiovascular disease risk factors in healthy elderly men	Sousa, Nelson; Mendes, Romeu; Abrantes, Catarina; Sampaio, Jaime; Oliveira, Jose
Long-term effects of high-intensity resistance and endurance exercise on plasma leptin and ghrelin in overweight individuals: the RESOLVE Study	Tremblay, Angelo; Dutheil, Frederic; Drapeau, Vicky; Metz, Lore; Lesour, Bruno; Chapier, Robert; [illegible]
Long-term high-intensity interval training associated with lifestyle modifications improves QT dispersion parameters in metabolic syndrome patients	Drigny, J.; Gremeaux, V.; Guiraud, T.; Gayda, M.; Juneau, M.; Nigam, A.
Long-term high-intensity interval training increases serum neurotrophic factors in elderly overweight and obese Chinese adults	Li, Xi; Han, Tianyu; Zou, Xu; Zhang, Han; Feng, Wenpin; Wang, Han; Shen, Yulin; Zhang, Li; Fa[illegible]
Long-term lifestyle intervention with optimized high-intensity interval training improves body composition, cardiometabolic risk, and exercise parameters in patients with abdominal obesity	Gremeaux, Vincent; Drigny, Joffrey; Nigam, Anil; Juneau, Martin; Guilbeault, Valerie; Latour, Elise[illegible]
Longitudinal affective response to high-intensity interval training and moderate-intensity continuous training in overweight women with polycystic ovary syndrome: A randomised trial	Patten, Rhiannon K.; Bourke, Matthew; McIlvenna, Luke C.; Moreno-Asso, Alba; Woessner, Ma[illegible]
Low Volume, High-Intensity Interval Training: A Pilot Study	Carl, Daniel L.; Bell, Jacqui; Bernhard, Andrea; Dyer, Ben; Phillips, James
Low Volume, Home-Based Weighted Step Exercise Training Can Improve Lower Limb Muscle Power and Functional Ability in Community-Dwelling Older Women	Mair, Jacqueline L.; De Vito, Giuseppe; Boreham, Colin A.
Low-Active Male Adolescents: A Dose Response to High-Intensity Interval Training	Logan, Greig Robert Melrose; Harris, Nigel; Duncan, Scott; Plank, Lindsay D.; Merien, Fabrice; Sc[illegible]
Low-Frequency High-Intensity Interval Training is an Effective Method to Improve Muscle Power in Lifelong Sedentary Aging Men: A Randomized Controlled Trial	Sculthorpe, Nicholas; Herbert, Peter; Grace, Fergal M.
Low-Frequency HIIT Improves Body Composition and Aerobic Capacity in Overweight Men	Chin, Edwin C.; Yu, Angus P.; Lai, Christopher W.; Fong, Daniel Y.; Chan, Derwin K.; Wong, Ste[illegible]
Low-level laser therapy prevents muscle apoptosis induced by a high-intensity resistance exercise in a dose-dependent manner	de Souza Oliveira, Claudia; de Oliveira, Helenita Antonia; Teixeira, Ighor Luiz Azevedo; Antonio, E[illegible]
Low-volume circuit versus high-volume periodized resistance training in women	Marx, J. O.; Ratamess, N. A.; Nindl, B. C.; Gotshalk, L. A.; Volek, J. S.; Dohi, K.; Bush, J. A.; Gó[illegible]
Low-Volume High-Intensity Aerobic Interval Training Is an Efficient Method to Improve Cardiorespiratory Fitness After Myocardial Infarction: PILOT STUDY FROM THE INTERFARCT PROJECT	Jayo-Montoya, Jon Ander; Maldonado-Martin, Sara; Aispuru, G. Rodrigo; Gorostegi-Anduaga, I[illegible]
Low-Volume High-Intensity Interval Training Is Sufficient to Ameliorate the Severity of Metabolic Syndrome	Ramos, Joyce S.; Dalleck, Lance C.; Borrani, Fabio; Beetham, Kassia S.; Wallen, Matthew P.; Ma[illegible]
Low-Volume High-Intensity Interval Training Versus Moderate- Intensity Continuous Training on Body Composition, Cardiometabolic Profile, and Physical Capacity in Older Women	Boukabous, Inès; Marcotte-Chénard, Alexis; Amamou, Taha; Boulay, Pierre; Brochu, Martin; Tes[illegible]
Lymphocyte enzymatic antioxidant responses to oxidative stress following high-intensity interval exercise	Fisher, G.; Schwartz, D. D.; Quindry, J.; Barberio, M. D.; Foster, E. B.; Jones, K. W.; Pascoe, D. [illegible]
Making a HIIT: study protocol for assessing the feasibility and effects of co-designing high-intensity interval training workouts with students and teachers	Duncombe, Stephanie L.; Barker, Alan R.; Price, Lisa; Walker, Jacqueline L.; Dux, Paul E.; Fox, A[illegible]
Martial arts-based high intensity interval training as a component of warfighter rehabilitation and tactical athlete fitness	Mills, Gavin L.; Johnson, Anthony E.
Martial arts-based high intensity interval training in the rehabilitation of combat amputees	Mills, Gavin L.; Tennent, David J.; Aldrete, Joseph F.; Johnson, Anthony E.
Masters athletes take longer to recover from high intensity exercise than training- matched younger athletes. Does increased protein intake enhance recovery?	Reaburn, P.; Doering, T.; Borges, N.
Maximal and ventilatory thresholds of oxygen uptake and rating of perceived exertion responses to water aerobic exercises	Alberton, C. L.; Antunes, A. H.; Beilke, D. D.; Pinto, S. S.; Kanitz, A. C.; Tartaruga, M. P.; Martins [illegible]
Maytenus ilicifolia Extract Increases Oxygen Uptake without Changes in Neuromuscular Fatigue Development during a High-Intensity Interval Exercise	Ferreira, Guilherme A.; Felippe, Leandro C.; Silva-Cavalcante, Marcos D.; De-Mello, Ana Paula A.; [illegible]
MCT1 genetic polymorphism influence in high intensity circuit training: a pilot study	Cupeiro, Rocio; Benito, Pedro J.; Maffulli, Nicola; Calderon, F. Javier; Gonzalez-Lamuno, Domin[illegible]
Meaningful weight loss in obese postmenopausal women: a pilot study of high-intensity interval training and wearable technology	Grossman, Joan A.; Arigo, Danielle; Bachman, Jessica L.
Mechanical and metabolic responses during a high-intensity circuit training workout in competitive runners	Di Michele, R.; Del Curto, L.; Merni, F.
Mediation effect of cardiorespiratory fitness on relationships between high-intensity interval training and body fat in overweight and obese adolescents	Domaradzki, Jaroslaw; Kozienia, Dawid; Popowczak, Marek
Men and Women Exhibit Similar Acute Hypotensive Responses After Low, Moderate, or High-Intensity Plyometric Training	Ramirez-Campillo, R.; Abad-Colil, F.; Vera, M.; Andrade, D. C.; Caniuqueo, A.; Martinez-Salazar, [illegible]
Menopause and high-intensity interval training: effects on body composition and physical performance	Buckinx, Fanny; Aubertin-Leheudre, Mylene
Metabolic and hormonal responses to isoenergetic high-intensity interval exercise and continuous moderate-intensity exercise	Peake, Jonathan M.; Tan, Sok Joo; Markworth, James F.; Broadbent, James A.; Skinner, Tina L. [illegible]
Metabolic effects of high-intensity interval training and essential amino acids	Hirsch, Katie R.; Greenwalt, Casey E.; Cabre, Hannah E.; Gould, Lacey M.; Brewer, Gabrielle J.; [illegible]
Metabolic effects of resistance or high-intensity interval training among glycemic control-nonresponsive children with insulin resistance	Alvarez, C.; Ramirez-Campillo, R.; Ramirez-Velez, R.; Martinez, C.; Castro-Sepulveda, M.; Alonso[illegible]
Metabolic rate in sedentary adults, following different exercise training interventions: The FIT-AGEING randomized controlled trial	Amaro-Gahete, Francisco J.; De-la-O, Alejandro; Jurado-Fasoli, Lucas; Sanchez-Delgado, Guille[illegible]
[Metabolic response to high intensity exercise training in sedentary hyperglycemic and hypercholesterolemic women]	Alvarez L, Cristian; Ramirez-Campillo, Rodrigo; Flores O, Marcelo; Henriquez-Olguin, Carlos; Car[illegible]
Methodological aspects in studies involving high-intensity interval training	Esteves, Joao Victor; Bertolini, Sonia Maria Marques Gomes; Andreato, Leonardo Vidal
Methods to match high-intensity interval exercise intensity in hypoxia and normoxia - A pilot study	Li, Yanchun; Li, Jia; Atakan, Muhammed M.; Wang, Zhenhuan; Hu, Yang; Nazif, Mostafa; Zarek[illegible]
Methods to reduce muscle damage and enhance short-term recovery after high intensity anaerobic exercise	Al-Nawaiseh, A. M.
MicroRNA Sequencing To Enhance Understanding Of Dose-Specific Resistance Training-Induced Muscle Mass Gains Among Older Adults	O'Bryan, Samia M.; Lavin, Kaleen M.; Bamman, Marcas M.
Microvascular adaptations in response to high intensity interval training in skeletal muscle	Cocks, M.; Shepherd, S.; Tipton, K. D.; Ranasinghe, A.; Barker, T.; Shaw, C. S.; Wagenmakers, [illegible]
MICT or HIIT +/- RT Programs for Altering Body Composition in Postmenopausal Women	Dupuit, M.; Rance, M.; Morel, C.; Bouillon, P.; Pereira, B.; Bonnet, A.; Maillard, F.; Duclos, M.; B[illegible]
Mid-life crisis or mid-life gains: 2 years of high-intensity exercise is highly beneficial for the middle-aged heart	Debi, Ryan; Lakin, Robert; Spector, Steven
Might High-Intensity Interval Exercise Be Remembered as More Pleasurable? An Attempt to Test the Peak-End Rule in the Exercise Context	Alves, Elaine Domingues; Julio, Ursula Ferreira; Panissa, Valeria Leme Goncalves; Franchini, Eme[illegible]
Moderate or vigorous intensity exercise: which is better for improving aerobic fitness?	Swain, David P.
Moderate versus high intensity interval exercise trainings on energy intake and appetite feelings in adolescents with obesity	Maud, M.; Julie, M.; Valerie, J.; Lore, M.; Elodie, C.; Charlotte, C.; Yves, B.; Martine, D.; David, T[illegible]
Moderate- and High-Intensity Exercise Improves Lipoprotein Profile and Cholesterol Efflux Capacity in Healthy Young Men	Stanton, Kelly M.; Kienzle, Vivian; Dinnes, Donna Lee M.; Kotchetkov, Irina; Jessup, Wendy; Kri[illegible]
Moderate- and high-intensity exercise lowers blood pressure in normotensive subjects 60 to 79 years of age	Braith, R. W.; Pollock, M. L.; Lowenthal, D. T.; Graves, J. E.; Limacher, M. C.
Moderate-intensity continuous exercise is superior to high-intensity interval training in the proportion of VO2peak responders after ACS	Trachsel, Lukas-Daniel; Nigam, Anil; Fortier, Annik; Lalonge, Julie; Juneau, Martin; Gayda, Mathi[illegible]
Moderate-intensity continuous training and high-intensity interval training improve cognition, and BDNF levels of middle-aged overweight men	de Lima, Neumir Sales; De Sousa, Ricardo Augusto Leoni; Amorim, Fabiano Trigueiro; Gripp, Fe[illegible]
Moderate-Intensity Continuous Training or High-Intensity Interval Training with or without Resistance Training for Altering Body Composition in Postmenopausal Women	Dupuit, Marine; Rance, Melanie; Morel, Claire; Bouillon, Patrice; Pereira, Bruno; Bonnet, Alban; M[illegible]
Moderate-Intensity Exercise and High-Intensity Interval Training Affect Insulin Sensitivity Similarly in Obese Adults	Ryan, Benjamin J.; Schleh, Michael W.; Ahn, Cheehoon; Ludzki, Alison C.; Gillen, Jenna B.; Var[illegible]
Modulation of Countermovement Jump-Derived Markers of Neuromuscular Function With Concurrent vs. Single-Mode Resistance Training	Pattison, Kristy J.; Drinkwater, Eric J.; Bishop, David J.; Stepto, Nigel K.; Fyfe, Jackson J.
Motives for Physical Activity and Physiological Variables as Predictors of Exercise Intentions Following a High Intensity Interval Training Protocol in College-Age Females	Langdon, Jody; Joseph, Sarah; Kendall, Kristina; Harris, Brandonn S.; McMillan, J. I. M.
Multi-Component Exercise with High-Intensity, Free-Weight, Functional Resistance Training in Pre-Frail Females: A Quasi-Experimental, Pilot Study	Bray, N. W.; Jones, G. J.; Rush, K. L.; Jones, C. A.; Jakobi, J. M.
Multimodal Therapy Involving High-Intensity Interval Training Improves the Physical Fitness, Motor Skills, Social Behavior, and Quality of Life of Boys With ADHD: A Randomized Controlled Study	Meßler, Carolin Friederike; Holmberg, Hans-Christer; Sperlich, Billy
Multiple bouts of high-intensity interval exercise reverse age-related functional connectivity disruptions without affecting motor learning in older adults	Greeley, Brian; Chau, Briana; Jones, Christina B.; Neva, Jason L.; Kraeutner, Sarah N.; Campbell[illegible]
Muscle irisin response to aerobic vs HIIT in overweight female adolescents	Archundia-Herrera, Carolina; Macias-Cervantes, Maciste; Ruiz-Munoz, Bernardo; Vargas-Ortiz, K[illegible]
Muscle metabolic responses during high-intensity intermittent exercise measured by (31)P-MRS: relationship to the critical power concept	Chidnok, Weerapong; DiMenna, Fred J.; Fulford, Jonathan; Bailey, Stephen J.; Skiba, Philip F.; V[illegible]
Muscle performance following an acute bout of plyometric training combined with low or high intensity weight exercise	Beneka, Anastasia G.; Malliou, Paraskevi K.; Missailidou, Victoria; Chatzinikolaou, Athanasios; Fa[illegible]
Muscular morphological adaptations of two whole-body high intensity interval training configurations	Moghaddam, Masoud; Estrada, Carlos A.; Baghurst, Timothy; Jacobson, Bert H.
The musculoskeletal effect of intense physical training of non-athletic youth corps conscripts	Egwu, M. O.
MUSIC LISTENING REDUCES BIOCHEMICAL AND PERCEPTUAL RESPONSES TO A SINGLE INTENSIFIED INTERVAL TRAINING. / SŁUCHANIE MUZYKI ZMNIEJSZA REAKCJE BIOCHEMICZNE I PERCEPCYJNE NA [illegible]	Obmiński, Zbigniew
Myokine Response to High-Intensity Interval vs. Resistance Exercise: An Individual Approach	He, Zihong; Tian, Ye; Valenzuela, Pedro L.; Huang, Chuanye; Zhao, Jiexiu; Hong, Ping; He, Zilin; [illegible]
Net Blood Pressure Reduction Following 9 Months of Lifestyle and High-Intensity Interval Training Intervention in Individuals With Abdominal Obesity	Sosner, Philippe; Bosquet, Laurent; Herpin, Daniel; Guilbeault, Valerie; Latour, Elise; Paquette-Ta[illegible]
The neurocognitive effects of a conducted electrical weapon compared to alcohol intoxication and a high intensity interval training regimen	Dawes, D. M.; Ho, J.; Vincent, A.; Nystrom, P.; Driver, B. E.
Neuromuscular adaptations to concurrent strength and endurance training. / Adaptations neuromusculaires a un entrainement simultane de force et d ' endurance	McCarthy, J. P.; Pozniak, M. A.; Agre, J. c
Neuromuscular Adaptations to Short-Term High-Intensity Interval Training in Female Ice-Hockey Players	Kinnunen, Juho-Ville; Piitulainen, Harri; Piirainen, Jarmo M.
Neutrophil and Monocyte Bactericidal Responses to 10 Weeks of Low-Volume High-Intensity Interval or Moderate-Intensity Continuous Training in Sedentary Adults	Bartlett, David B.; Shepherd, Sam O.; Wilson, Oliver J.; Adlan, Ahmed M.; Wagenmakers, Anton [illegible]
A new dose of maximal-intensity interval training in hypoxia to improve body composition and hemoglobin and hematocrit levels: a pilot study	Camacho-Cardenosa, Marta; Camacho-Cardenosa, Alba; Martinez Guardado, Ismael; Marcos-Se[illegible]
No Acute Effect of Reduced-exertion High-intensity Interval Training (REHIT) on Insulin Sensitivity	Metcalfe, R.; Fawkner, S.; Vollaard, N.
No Change in Inhibitory Control or P3 Following Different High-Intensity Interval Exercise Modalities	Drollette, Eric S.; Johnson, Megan N.; Meadows, Caroline C.
No effect of meal intake on physiological or perceptual responses to self-selected high intensity interval exercise (HIIE)	Astorino, Todd A.; Sherrick, Sarah; Mariscal, Monique; Jimenez, Vianney Camarillo; Stetson, Kelli[illegible]
No evidence of brown adipose tissue activation after 24 weeks of supervised exercise training in young sedentary adults in the ACTIBATE randomized controlled trial	Martinez-Tellez, Borja; Sanchez-Delgado, Guillermo; Acosta, Francisco M.; Alcantara, Juan M. A. [illegible]
Non-traditional training in youth soccer players: Effects on agility and on sprint performance	Carvutto, Roberto; Damasco, Claudio; De Candia, Michele
Nordic Walking Rather Than High Intensity Interval Training Reduced Myostatin Concentration More Effectively in Elderly Subjects and the Range of This Drop Was Modified by Metabolites of Vitamin D	Micielska, Katarzyna; Flis, Marta; Kortas, Jakub Antoni; Rodziewicz-Flis, Ewa; Antosiewicz, Jedrz[illegible]
A novel exercise regime to reduce cardiometabolic risk in overweight sedentary individuals	Keating, S.; MacHan, E.; Rooney, K.; O'Connor, H.; Caterson, I.; Johnson, N.
A novel form of high intensity interval training on CVD risk factors in sedentary and moderate risk individuals	Roxburgh, B.; Reading, S.; Dalleck, L.
Nutritional strategies to enhance performance of high-intensity exercise	Volek, J. S.; Houseknecht, K.; Kraemer, W. J.
Obesity does not modulate men's eating behavior after a high intensity interval exercise session: an exercise trial	Terra, Caio M.; Botero, Joao P.; Antunes, Jaddy; Haddock, Bryan; Malik, Neal; Thivel, David; Pra[illegible]
Obesity promotes oxidative stress and exacerbates blood-brain barrier disruption after high-intensity exercise	Roh, Hee-Tae; Cho, Su-Youn; So, Wi-Young
Obesity-prone children can benefit from high-intensity exercise. / L'enfant obese peut tirer profit de l'intensite de l'exercice	Hunter, G. R.; Bamman, M. M.; Hester, D.
Ocular Accommodative Response is Modulated as a Function of Physical Exercise Intensity	Vera, Jesus; Luque-Casado, Antonio; Redondo, Beatriz; Cardenas, David; Jimenez, Raimundo; [illegible]
Oligofructose-Enriched Inulin Intake, Gut Microbiome Characteristics, and the VO2 Peak Response to High-Intensity Interval Training in Healthy Inactive Adults	Williams, Camilla J.; Torquati, Luciana; Li, Zhixiu; Lea, Rodney A.; Croci, Ilaria; Keating, Eliza; Lit[illegible]
One session of high-intensity interval training (HIIT) every 5 days, improves muscle power but not static balance in lifelong sedentary ageing men: A randomized controlled trial	Sculthorpe, Nicholas F.; Herbert, Peter; Grace, Fergal
Opioid Release after High-Intensity Interval Training in Healthy Human Subjects	Saanijoki, Tiina; Tuominen, Lauri; Tuulari, Jetro J.; Nummenmaa, Lauri; Arponen, Eveliina; Kallio[illegible]
Optimal Values of Body Composition for the Lowest Risk of Failure in Tabata Training's Effects in Adolescents: A Pilot Study	Domaradzki, Jaroslaw; Rokita, Andrzej; Koziena, Dawid; Popowczak, Marek
Optimizing patient benefit from CRT response with the addition of high intensity interval training-a randomized controlled trial	Pinto, R.; Abreu, A.; Santos, V.; Melo, X.; Cunha, P.; Oliveira, M.; Soares, R.; Ferreira, R.; Fernha[illegible]
Optimizing Sleep in Older Adults: Where Does High-Intensity Interval Training Fit?	Bullock, Alexis; Kovacevic, Ana; Kuhn, Tara; Heisz, Jennifer J.
Oral contraceptive use and adaptation to high intensity interval training in recreationally active women	Schaumberg, M.
Order of same-day concurrent training influences some indices of power development, but not strength, lean mass, or aerobic fitness in healthy, moderately-active men after 9 weeks of training	Lee, Matthew J. C.; Ballantyne, James K.; Chagolla, Javier; Hopkins, William G.; Fyfe, Jackson J.[illegible]
Original Research: Effect of sprint and strength training on glucoregulatory hormones: Effect of advanced age	Sellami, Maha; Ben Abderrahman, Abderraouf; Kebsi, Wiem; De Sousa, Maysa Vieira; Zouhal, Ha[illegible]
Oscillation of tissue oxygen index in non-exercising muscle during exercise	Yano, T.; Afroundeh, R.; Shirakawa, K.; Lian, C. S.; Shibata, K.; Xiao, Z.; Yunoki, T.
Osteocalcin and its forms respond similarly to exercise in males and females	Hiam, D.; Landen, S.; Jacques, M.; Voisin, S.; Alvarez-Romero, J.; Byrnes, E.; Chubb, P.; Leving[illegible]
Outcomes of a 12-week functional exercise programme for institutionalised elderly people	Gillies, E.; Aitchison, T.; MacDonald, J.; Grant, S.
Oxidative Stress in Young Football (Soccer) Players in Intermittent High Intensity Exercise Protocol	Escobar, Mariana; Oliveira, Max William Soares; Behr, Guilherme AntÔNio; Zanotto-Filho, Alfeu; [illegible]
Oxygen uptake and the activation of glycolysis during exercise of high intensity	Hamar, D.; Komadel, L.; Kadlecik, J.
Oxygen uptake dynamics during high intensity exercise in children and adults (Dynamique de la consommation d'oxygene lors d'un exercice de haute intensite chez les enfants et les adultes)	Armon, Y.; Couper, D.; Flores, R.; Zanconato, S.
Paracentral acute middle maculopathy following high-intensity interval training	Alten, Florian; Schmitz, Boris
Paracentral acute middle maculopathy following high-intensity interval training	Arjmand, Pamian; Mandelcorn, Efrem D.
Parathyroid hormone concentrations during and after two periods of high intensity exercise with and without an intervening recovery period	Bouassida, A.; Zalleg, D.; Zaouali Ajina, M.; Gharbi, N.; Duclos, M.; Richalet, J. P.; Tabka, Z.
Participation in a 9-month selected physical exercise programme enhances psychological well-being in a prison population	Battaglia, Claudia; di Cagno, Alessandra; Fiorilli, Giovanni; Giombini, Arrigo; Borrione, Paolo; Bar[illegible]
Passive versus active recovery during high-intensity intermittent exercises	Dupont, Gregory; Moalla, Wassim; Guinhouya, Comlavi; Ahmaidi, Said; Berthoin, Serge
Patients with Adolescent Idiopathic Scoliosis Have Higher Metabolic Cost during High-Intensity Interval Training	Lau, Rufina Wing-Lum; Kwan, Rachel Lai-Chu; Cheng, Jack Chun-Yiu; Hui, Stanley Sai-Chuen; [illegible]
Peptide YY, acylated ghrelin and leptin concentrations following moderate and high intensity of exercise in males	Sitaletdetkul, S.; Thompson, D.; Stokes, K.
Perception of effort during high intensity exercise at low, moderate and high wet bulb globe temperature (Perception de l'effort lors d'efforts de haute intensite sour des temperatures faiblement, moyenne[illegible]	Glass, S.; Knowlton, R.; Becque, M.
Perceptions of participating in high-intensity functional exercise among older people dependent in activities of daily living (ADL)	Lindelof, N.; Rosendahl, E.; Gustafsson, S.; Nygaard, J.; Gustafson, Y.; Nyberg, L.
Perceptual and Cardiorespiratory Responses to High-Intensity Interval Exercise in Adolescents: Does Work Intensity Matter?	Malik, Adam A.; Williams, Craig A.; Weston, Kathryn L.; Barker, Alan R.
Perceptual Responses to High- and Moderate-Intensity Interval Exercise in Adolescents	Malik, Adam A.; Williams, Craig A.; Weston, Kathryn L.; Barker, Alan R.
Peripheral and central fatigue after high intensity resistance circuit training	Marquez, Gonzalo; Romero-Arenas, Salvador; Marin-Pagan, Cristian; Vera-Ibanez, Antonio; Fern[illegible]
Peripheral heart action (PHA) training as a valid substitute to high intensity interval training to improve resting cardiovascular changes and autonomic adaptation	Piras, Alessandro; Persiani, Michela; Damiani, Nicholas; Perazzolo, Monica; Raffi, Milena
Personalized metabolomics for predicting glucose tolerance changes in sedentary women after high-intensity interval training	Kuehnbaum, Naomi L.; Gillen, Jenna B.; Gibala, Martin J.; Britz-McKibbin, Philip
Personalized Moderate-Intensity Exercise Training Combined with High-Intensity Interval Training Enhances Training Responsiveness	Byrd, Bryant R.; Keith, Jamie; Keeling, Shawn M.; Weatherwax, Ryan M.; Nolan, Paul B.; Ramos [illegible]
PH10: effects of HIIT intervention on PGC-1α and AdipoR1 genes expressions and body composition in obese individuals	Za'don, N. H. A.; Mohd Fauzi, N. F.; Tuan Othman, S. I.; Muhammad Kamal, A. F.; Ismail, F.; A[illegible]
Physical activity interventions: effects of duration and intensity	Buchan, D. S.; Ollis, S.; Thomas, N. E.; Buchanan, N.; Cooper, S. M.; Malina, R. M.; Baker, J. S.
Physical Activity Participation After a 16-Week Supervised Workplace Exercise RCT With a 15-Month Follow-Up	Hunter, Jayden R.; Gordon, Brett A.; Huynh, Minh; Benson, Amanda C.
Physical Demands of Multimodal Training Competitions and Their Relationship to Measures of Performance	Jagim, Andrew R.; Rader, Owen; Jones, Margaret T.; Oliver, Jonathan M.
Physiological Adaptations to High-Intensity Interval and Continuous Training in Kayak Athletes	Papandreou, Apostolos; Philippou, Anastassios; Zacharogiannis, Elias; Maridaki, Maria
Physiological Adaptations to Sprint Interval Training with Matched Exercise Volume	Lee, Chia-Lun; Hsu, Wei-Chieh; Cheng, Ching-Feng

Title	Authors
Physiological adaptations to strength and circuit training in postmenopausal women with bone loss	Brentano, Michel A.; Cadore, Eduardo L.; Da Silva, Eduardo M.; Ambrosini, Anelise B.; Coertjens, M.; Petkowicz, Rosemary; Viero, Itamara; Kruel, Luiz F. M.
Physiological adjustments to higher- or lower-intensity exercise in elders. / Les adaptations physiologiques a l' exercice de haute ou basse intensite chez des veterans	Badenhop, D. T.; Cleary, P. A.; Schaal, S. F.; Fox, E. L.; Bartels, R. L.
PHYSIOLOGICAL AND BIOCHEMICAL CHANGES IN ADOLESCENTS AFTER HIGH-INTENSITY TRAINING	Feng, H.; Liu, W.
The physiological and metabolic responses of female hockey players toshortterm high intensity training (Les r/ponses physiologiques et metaboliques a un entrainement a court terme de haute intensite, c	Cheetham, M. E.; Williams, G.
Physiological and Psychological Responses during Low-Volume High-Intensity Interval Training Sessions with Different Work-Recovery Durations	Farias-Junior, Luiz Fernando; Macedo, Geovani Araujo Dantas; Browne, Rodrigo Alberto Vieira; Freire, Yuri Alberto; Oliveira-Dantas, Filipe Fernandes; Schwade, Daniel; Mortatti, Arnaldo Luis; Santos, Tony Meirel [illegible]
Physiological and Psychological Responses to Three Distinct Exercise Training Regimens Performed in an Outdoor Setting: Acute and Delayed Response	Benitez-Flores, Stefano; Magallanes, Carlos A.; Alberton, Cristine Lima; Astorino, Todd A.
Physiological Effects and Inter-Individual Variability to 12 Weeks of High Intensity-Interval Training and Dietary Energy Restriction in Overweight/Obese Adult Women	Andrade-Mayorga, Omar; Martinez-Maturana, Nicolas; Salazar, Luis A.; Diaz, Erik
Physiological Responses During Short And Long High-intensity Interval And Continuous Exercise	Tschakert, Gerhard; Groeschl, Werner; Hofmann, Peter
Physiological responses of fire fighting recruits to high intensity training	Pipes, T. V.
Physiology of Activins/Follistatins: Associations With Metabolic and Anthropometric Variables and Response to Exercise	Perakakis, Nikolaos; Mougios, Vassilis; Fatouros, Ioannis; Siopi, Aikaterina; Draganidis, Dimitrios; Peradze, Natia; Ghaly, Wael; Mantzoros, Christos S.
A Pilot Study on In-Task Affect Predicting Free-Living Adherence to HIIT and MICT	Ivanova, Elena; Sadikaj, Gentiana; Bourne, Jessica E.; Beauchamp, Mark; Little, Jonathan P.; Jung, Mary E.
A pilot study to assess the effect of acute exercise on brain glutathione	Rai, Sahara; Chowdhury, Almul; Reniers, Renate L. E. P.; Wood, Stephen J.; Lucas, Samuel J. E.; Aldred, Sarah
Plasma chemerin in young untrained men: association with cardio-metabolic traits and physical performance, and response to intensive interval training	Ouerghi, Nejmeddine; Fradj, Mohamed Kacem Ben; Khammassi, Marwa; Feki, Moncef; Kaabachi, Naziha; Bouassida, Anissa
Plasma Irisin Modestly Increases during Moderate and High-Intensity Afternoon Exercise in Obese Females	Winn, Nathan C.; Grunewald, Zachary I.; Liu, Ying; Heden, Timothy D.; Nyhoff, Lauren M.; Kanaley, Jill A.
Plasma pentraxin 3 and glucose kinetics following acute high-intensity interval exercise versus continuous moderate-intensity exercise in healthy men	Slusher, Aaron L.; Whitehurst, Michael; Maharaj, Arun; Dodge, Katelyn M.; Fico, Brandon G.; Mock, J. Thomas; Huang, Chun-Jung
Plasma potassium changes with high intensity dynamic exercise. / Modifications du potassium plasmatique lors d ' un exercice dynamique de forte intensite	Sejersted, O. M.; Medbo, J. I.
"Plyo Play": A Novel Program of Short Bouts of Moderate and High Intensity Exercise Improves Physical Fitness in Elementary School Children	Faigenbaum, Avery D.; Farrell, Anne C.; Radler, Tracy; Zbojovsky, Dan; Chu, Donald A.; Ratamess, Nicholas A.; Kang, Jie; Hoffman, Jay R.
Plyometric exercise combined with high-intensity interval training improves metabolic abnormalities in young obese females more so than interval training alone	Racil, Ghazi; Zouhal, Hassane; Elmontassar, Wassim; Ben Abderrahmane, Abdedaouf; De Sousa, Maysa Vieira; Chamari, Karim; Amri, Mohamed; Coquart, Jeremy B.
Polarized training has greater impact on key endurance variables than threshold, high intensity, or high volume training	Stoggl, Thomas; Sperlich, Billy
Polarized Versus High-Intensity Multimodal Training in Recreational Runners	Carnes, Andrew J.; Mahoney, Sara E.
Positive Affective and Enjoyment Responses to Four High-Intensity Interval Exercise Protocols	Marques, Marcelo; Alves, Elaine; Henrique, Nayana; Franchini, Emerson
A Post hoc analysis on rhythm and high intensity interval training in cardiac resynchronization therapy	Melo, Xavier; Abreu, Ana; Santos, Vanessa; Cunha, Pedro; Oliveira, Mario; Pinto, Rita; Carmo, Miguel; Fernhall, Bo; Santa-Clara, Helena
Post-exercise heart rate variability of endurance athletes after different high-intensity exercise interventions	Kaikkonen, P.; Rusko, H.; Martinmaki, K.
Post-exercise hypotension in response to high-intensity interval exercise: Potential mechanisms	Perrier-Melo, Raphael Jose; Germano-Soares, Antonio Henrique; Freitas Brito, Aline; Vilela Dantas, Iago; da Cunha Costa, Manoel
Post-exercise hypotension time-course is influenced by exercise intensity: a randomised trial comparing moderate-intensity, high-intensity, and sprint exercise	Jones, Matthew D.; Munir, Muhammad; Wilkonski, Anna; Ng, Kelly; Beynon, Guy; Keech, Andrew
Post-plyometric exercise hypotension and heart rate in normotensive individuals: influence of exercise intensity	Arazi, Hamid; Asadi, Abbas; Rahimzadeh, Mehdi; Moradkhani, Amir-Hossein
Postexercise Hypotension After Moderate-Intensity Continuous and High-Intensity Interval Exercise	Angadi, Siddhartha; Fumanek, Cynthia; Hahn, Dameon; Gaesser, Glenn A.
Postexercise hypotension and heart rate variability response after water- and land-based high-intensity interval exercise in prehypertensive obese men	Sriton, Bhuwanat; Ruangthai, Ratree; Phoemsapthawee, Jatuporn
Postprandial lipoprotein profile in two modes of high-intensity intermittent exercise	Panissa, Valeria Leme Goncalves; Julio, Ursula Ferreira; Diniz, Tiago Aparecido; de Moura Mello Antunes, Barbara; Lira, Fabio Santos; Takito, Monica Yuri; Franchini, Emerson
Potential Application of Low-Intensity Focused Ultrasound in Rapidly Relieving Delayed-Onset Muscle Soreness Induced by High-Intensity Exercise	Xia, Yi; Li, Jianhu; Wang, Disen; Chen, Jinyun; Shen, Mingxue; Li, Faqi; Wang, Yan; Jiang, Ping
Potential Causes of Elevated REE after High-Intensity Exercise	Hunter, Gary R.; Moellering, Douglas R.; Carter, Stephen J.; Gower, Barbara A.; Bamman, Marcas M.; Hornbuckle, Lyndsey M.; Plaisance, Eric P.; Fisher, Gordon [illegible]
Potential mechanisms uncovered for the cardioprotective effects of high-intensity interval training	Chen, Anzhong; Li, Man; Jia, Shuxian
The practice of active rest by workplace units improves personal relationships, mental health, and physical activity among workers	Michishita, Ryoma; Jiang, Ying; Ariyoshi, Daisuke; Yoshida, Marie; Moriyama, Hideko; Yamato, Hiroshi
Pragmatic community based exercise prescription of hiit-results of an RCT	Roy, M.; Osborne, H. R.; Meredith-Jones, K.; Brown, R. C.; Williams, S. M.; Jospe, M.; Taylor, R.
PRE-EXERCISE CARBOHYDRATE CONSUMPTION AND PERCEIVED EXERTION IN ADOLESCENT MALES PERFORMING HIGH-INTENSITY INTERMITTENT EXERCISE	Mahon, A. D.; Lee, J. D.; Hanna, L. E.; Guth, L. M.; Konopka, A. R.
Preconditioning Activities to Enhance Repeated High-Intensity Efforts in Elite Rugby Union Players	Vachon, Adrien; Berryman, Nicolas; Mujika, Inigo; Paquet, Jean-Baptiste; Bosquet, Laurent
Preliminary results of a randomized, controlled clinical trial examining the impact of a structured-exercise training program across various erectile domains, cardiorespiratory fitness and endothelial functi	Chung, E.; Vear, N.; Skinner, T.; Coombes, J.; Rhee, H.
Prescribing high-intensity interval exercise by rating of perceived exertion in young individuals	Marcal, Isabela R.; Falqueiro, Pedro G.; Fernandes, Bianca; Ngomane, Awassi Y.; Amaral, Vanessa T.; Guimaraes, Guilherme V.; Ciolac, Emmanuel G.
Preservation of skeletal muscle mitochondrial content in older adults: relationship between mitochondria, fibre type and high-intensity exercise training	Wyckelsma, Victoria L.; Levinger, Itamar; McKenna, Michael J.; Formosa, Luke E.; Ryan, Michael T.; Petersen, Aaron C.; Anderson, Mitchell J.; Murphy, Robyn M [illegible]
Prevalence of Non-responders for Blood Pressure and Cardiometabolic Risk Factors Among Prehypertensive Women After Long-Term High-Intensity Interval Training	Alvarez, Cristian; Ramirez-Campillo, Rodrigo; Cristi-Montero, Carlos; Ramirez-Velez, Robinson; Izquierdo, Mikel
Prevalence of Non-responders for Glucose Control Markers after 10 Weeks of High-Intensity Interval Training in Adult Women with Higher and Lower Insulin Resistance	Alvarez, Cristian; Ramirez-Campillo, Rodrigo; Ramirez-Velez, Robinson; Izquierdo, Mikel
Process Evaluation of Project FFAB (Fun Fast Activity Blasts): A Multi-Activity School-Based High-Intensity Interval Training Intervention	Weston, Kathryn L.; Innerd, Alison; Azevedo, Liane B.; Bock, Susan; Batterham, Alan M.
Program Evaluation Of Fitness- And Skill-based High-intensity Interval Training (hiit) In Elementary School Physical Education	McKee, Katherine L.; Ricci, Jeanette M.; Currie, Katharine D.; Astorino, Todd A.; Erickson, Karl; Pfeiffer, Karin A.
A program of circuit resistance training under hypobaric hypoxia conditions improves the anaerobic performance of athletes	Álvarez-Herms, J.; Julià-Sánchez, S.; Corbi, F.; Pagès, T.; Viscor, G.
Programming may matter the most. Response to "Metabolic effects of two high-intensity circuit training protocols: Does sequence matter?" by Nunez et al. (2020)	Vasconcelos, Breno Berny; Del Vecchio, Fabricio Boscolo
Protein Composition of Circulating Extracellular Vesicles Immediately Changed by Particular Short Time of High-Intensity Interval Training Exercise	Kobayashi, Yoshinao; Eguchi, Akiko; Tamai, Yasuyuki; Fukuda, Sanae; Tempaku, Mina; Izuoka, Kiyora; Iwasa, Motoh; Takei, Yoshiyuki; Togashi, Kenji
Psycho-Physiological Responses to a 4-Month High-Intensity Interval Training-Centered Multidisciplinary Weight-Loss Intervention in Adolescents with Obesity	Khammassi, Marwa; Miguet, Maud; Julian, Valerie; Cardenoux, Charlotte; Boirie, Yves; Duclos, Martine; Pereira, Bruno; Thivel, David
Psychological responses to HIIT and MICT over a 2-week progressive randomized trial among individuals at risk of type 2 diabetes	Santos, Alexandre; Stork, Matthew J.; Locke, Sean R.; Jung, Mary E.
Psychological Responses To HIIT Exercise Of Different Intensity Slopes	Korte, Sandra; Mastrofini, Gianna F.; Patel, Sarina J.; Phillips, Keegan G.; Sheriff, Paul P.; Hudson, Lauren A.; Richter, Haylee E.; Kilpatrick, Marcus W.
Psychological responses, muscle damage, inflammation, and delayed onset muscle soreness to high-intensity interval and moderate-intensity continuous exercise in overweight men	Farias-Junior, Luiz Fernando; Browne, Rodrigo Alberto Vieira; Freire, Yuri Alberto; Oliveira-Dantas, Filipe Fernandes; Lemos, Telma Maria Araujo Moura; Galvao-C [illegible]
Psychophysiological Intervention to Improve Preparedness in Military Special Operations Forces	Tornero-Aguilera, Jose Francisco; Pelarigo, Jailton Gregorio; Clemente-Suarez, Vicente Javier
Quantification of motor ability components in response to high intensity interval training and moderate intensity continuous training among college women athletes	Lakshmi Prabha, V.; John Parthiban, I.
A randomized 9-month study of blood pressure and body fat responses to aerobic training versus combined aerobic and resistance training in older men	Sousa, Nelson; Mendes, Romeu; Abrantes, Catarina; Sampaio, Jaime; Oliveira, Jose
A randomized control trial investigating high-intensity interval training and mental health: a novel non-responder phenotype related to anxiety in young adults	Lucibello, K. M.; Paolucci, E. M.; Graham, J. D.; Heisz, J. J.
A randomized controlled trial of fall prevention by a high-intensity functional exercise program for older people living in residential care facilities	Rosendahl, Erik; Gustafson, Yngve; Nordin, Ellinor; Lundin-Olsson, Lillemor; Nyberg, Lars
Randomized controlled trial of the effects of high intensity and low-to-moderate intensity exercise on physical fitness and fatigue in cancer survivors: results of the Resistance and Endurance exercise After	Kampshoff, Caroline S.; Chinapaw, Mai J. M.; Brug, Johannes; Twisk, Jos W. R.; Schep, Goof; Nijziel, Marten R.; van Mechelen, Willem; Buffart, Laurien M.
Randomized controlled trial of training intensity in adiposity	Cho, J. K.; Lee, S. H.; Lee, J. Y.; Kang, H. S.
Rating of perceived exertion after 12 weeks of high-intensity, intermittent sprinting	Heydari, Mehrdad; Boutcher, Stephen H.
Rating of Perceived Exertion During Circuit Weight Training: a Concurrent Validation Study	Aniceto, R. R.; Ritti-Dias, R. M.; Dos Prazeres, T. M.; Farah, B. Q.; de Lima, F. F.; do Prado, W. L.
Ratings of Perceived Exertion and Self-reported Mood State in Response to High Intensity Interval Training. A Crossover Study on the Effect of Chronotype	Vitale, Jacopo A.; La Torre, Antonio; Baldassarre, Roberto; Piacentini, Maria F.; Bonato, Matteo
Ready for Combat, Psychophysiological Modifications in a Close-Quarter Combat Intervention After an Experimental Operative High-Intensity Interval Training	Tornero-Aguilera, Jose F.; Fernandez-Elias, Valentin E.; Clemente-Suarez, Vicente J.
Recovery of muscle power after high intensity short term exercise, comparing boys and men (Recuperation de la puissance musculaire apres un effort a court terme et de haute intensite. Comparaison ent	Hebestreit, H.; Mimura, K.; Bar Or, O.
Redox regulation of post-prandial vascular endothelial dysfunction prophylactic benefits of high-intensity exercise	Bailey, Damian M.
Reduced Exertion High-Intensity Interval Training is More Effective at Improving Cardiorespiratory Fitness and Cardiometabolic Health than Traditional Moderate-Intensity Continuous Training	Cuddy, Tom F.; Ramos, Joyce S.; Dalleck, Lance C.
Reduced-Exertion High-Intensity Interval Training as a Novel Therapeutic Approach for Insomnia	Atef, Hady; Muka, Taulant
Reducing training frequency from 3 or 4 sessions/week to 2 sessions/week does not attenuate improvements in maximal aerobic capacity with reduced-exertion high-intensity interval training (REHIT)	Thomas, Gavin; Songsorn, Preeyaphorn; Gorman, Aimee; Brackenridge, Ben; Cullen, Tom; Fitzpatrick, Ben; Metcalfe, Richard S.; Vollaard, Niels B. J.
Reduction in Butyrylcholinesterase Activity and Cardiovascular Risk Factors in Obese Adolescents after 12-Weeks of High-Intensity Interval Training	Pizzi, Juliana; Furtado-Alle, Lupe; Schiavoni, Durcelina; Lopes, Wendell Arthur; da Rosa Silva, Larissa; Bono, Gleyse Freire; Rodrigues Souza, Ricardo Lehtonen; [illegible]
Regarding High-Intensity Interval Training and Left Ventricular Mechanics	Stohr, Eric J.; Balmain, Bryce N.; Sabapathy, Surendran
Regional changes in indices of bone strength of upper and lower limbs in response to high-intensity impact loading or high-intensity resistance training	Lambert, Conor; Beck, Belinda R.; Harding, Amy T.; Watson, Steven L.; Weeks, Benjamin K.
Regulation of plasma fatty acid oxidation during low- and high-intensity exercise	Sidossis, L. S.; Gastaldelli, A.; Klein, S.; Wolfe, R. R.
Rehabilitation using high-intensity physical training and long-term return-to-work in cancer survivors	Thijs, Karin M.; de Boer, Angela G. E. M.; Vreugdenhil, Gerard; van de Wouw, Agnes J.; Houterman, Saskia; Schep, Goof
The relationship between heart period variability and VO2 peak response to high intensity intermittent exercise training	Boutcher, S.; Park, Y.; Dunn, S.; Boutcher, Y.
Relationship between selected physiological responses, perceived exertion, and saliva cortisol level following maximal progressive and high intensity exercise in healthy female subjects	Ordor, O.; Grissom, F.; Randall, O.; Berger, A.; Leppo, M.
[The relationships between the basic checklist score, physical function, and improvement in physical function]	Arai, Takeshi; Obuchi, Shuichi; Sato, Mutsumi; Noro, Mitumi
The Relative Importance of Age at Peak Height Velocity and Fat Mass Index in High-Intensity Interval Training Effect on Cardiorespiratory Fitness in Adolescents: A Randomized Controlled Trial	Domaradzki, Jaroslaw; Koziena, Dawid; Popowczak, Marek
Repeated Plyometric Exercise Attenuates Blood Glucose in Healthy Adults	Barillas, Saldiam R.; Watkins, Casey M.; Wong, Megan A.; Dobbs, Ian J.; Archer, David C.; Munger, Cameron N.; Galpin, Andrew J.; Coburn, Jared W.; Brown, L [illegible]
Repeated transient mRNA bursts precede increases in transcriptional and mitochondrial proteins during training in human skeletal muscle	Perry, Christopher G. R.; Lally, James; Holloway, Graham P.; Heigenhauser, George J. F.; Bonen, Arend; Spriet, Lawrence L.
Reply to "Programming may matter most." Response to "Metabolic effects of two high-intensity circuit training protocols: Does sequence matter?"	Nunez, Tony P.; Amorim, Fabiano T.; Beltz, Nicholas M.; Mermier, Christine M.; Moriarty, Terence A.; Nava, Roberto C.; VanDusseldorp, Trisha A.; Kravitz, Len
Reproducibility of ambulatory blood pressure after high-intensity interval training sessions in healthy individuals	Costa, Eduardo C.; Dantas, Teresa C. B.; Farias-Junior, Luiz F.; Browne, Rodrigo A. V.; Correia, Marilia A.; Forjaz, Claudia L. M.; Ritti-Dias, Raphael M.
Residual Impact of Concurrent, Resistance, and High-Intensity Interval Training on Fasting Measures of Glucose Metabolism in Women With Insulin Resistance	Alvarez, Cristian; Ciolac, Emmanuel Gomes; Guimaraes, Guilherme Veiga; Andrade, David C.; Vasquez-Munoz, Manuel; Monsalves-Alvarez, Matias; Delgado-Floo [illegible]
Resistance training and detraining effects on flexibility performance in the elderly are intensity-dependent	Fatouros, Ioannis G.; Kambas, Antonios; Katrabasas, Ioannis; Leontsini, Diamanda; Chatzinikolaou, Athanasios; Jamurtas, Athanasios Z.; Douroudos, Ioannis; [illegible]
Resistance Training Contribute to the Aerobic Components of an Exercise Session in Adults but not as Much in Older Adults	Bharti, Neha; Hrubeniuk, Travis; Mayo, Andrea; Senechal, Martin; Bouchard, Danielle R.
Resistance training preserves high-intensity interval training induced improvements in skeletal muscle capillarization of healthy old men: a randomized controlled trial	Leuchtmann, Aurel B.; Mueller, Sandro Manuel; Aguayo, David; Petersen, Jens A.; Ligon-Auer, Maria; Fluck, Martin; Jung, Hans H.; Toigo, Marco
Resisted and Endurance High Intensity Interval Training for Combat Preparedness	Tornero-Aguilera, Jose Grancisco; Clemente-Suarez, Vicente Javier
Resolving the determinants of high-intensity exercise performance	Poole, David C.
Respiratory and Perceptual Responses to High-Intensity Interval Exercise in Obese Adults	Wong, Michael W. H.; Ross, Nicholas A.; Chien, Lung-Chang; Bhammar, Dharini M.
Respiratory indexes and inflammatory marker change by different exercise training	Tartibian, B.; Khayat, S. M. A.
Respiratory muscle function in trained and untrained adolescents during short term high intensity exercise (fonction du muscle respiratoire chez des adolescents entraines ou non lors d'un exercice de bre	Choukroun, M.; Kays, C.; Gioux, M.; Techoueyres, P.; Guenard, H.
Response of coagulation and anti-coagulant factors of elite athletes following acute resistance and high-intensity interval training	Karampour, Sedigheh; Gaeini, Abbas A.
Response of obese schoolchildren to high-intensity interval training applied in the school context	Espinoza-Silva, Miguel; Latorre-Roman, Pedro Angel; Parraga-Montilla, Juan; Caamano-Navarrete, Felipe; Jerez-Mayorga, Daniel; Delgado-Floody, Pedro
THE RESPONSE OF PLASMA ADROPIN LEVELS AND INSULIN RESISTANCE INDEX TO HIGH INTENSITY INTERVAL TRAINING IN SEDENTARY MEN	Rezaeimanesh, D.
Response of selected physiological variables and perceived exertion to high intensity weight training in highly trained and beginning weight trainers	Noble, B. J.; Kraemer, W. J.; Clark, M.
Response of serum inflammatory protein to exercise as a physiological stress: two different training protocol	Tartibian, B.; Khayat, S. M. A.
The response of some of hematological indicators to 6-week high-intensity interval training along with omega-3 supplement intake in sedentary boys	Pour, S. K.; Nayebifar, S.
Response of testosterone and cortisol concentrations to high-intensity resistance exercise following creatine supplementation	Volek, J.; Boetes, M.; Bush, J. A.; Putukian, M.; Sebastianelli, W. J.; Kraemer, W. J.
Response: Is High-Intensity Functional Training (HIFT)/CrossFit Safe for Military Fitness Training?	Poston, Walker S. C.; Haddock, Christopher K.; Heinrich, Katie M.; Jahnke, Sara A.; Jitnarin, Nattinee; Batchelor, David B.; Feito, Yuri; Suminski, Richard R.
Responses and adaptations to high intensity exercise in humans. (Abstract)	Greenhaff, P.
Resting cardiac autonomic activity and body composition following a 16-week high-intensity functional training intervention in women: A pilot study	Bechke, Emily; Kliszczewicz, Brian; Feito, Yuri; Kelemen, Hannah; Nickerson, Brett
Resveratrol supplementation does not augment performance adaptations or fibre-type-specific responses to high-intensity interval training in humans	Scribbans, Trisha D.; Ma, Jasmin K.; Edgett, Brittany A.; Vorobej, Kira A.; Mitchell, Andrew S.; Zelt, Jason G. E.; Simpson, Craig A.; Quadrilatero, Joe; Gurd, Bren [illegible]
Risk of Falls in Healthy Older Adults: Benefits of High-Intensity Interval Training Using Lower Body Suspension Exercises	Jimenez-Garcia, Jose D.; Hita-Contreras, Fidel; de la Torre-Cruz, Manuel; Fabrega-Cuadros, Raquel; Aibar-Almazan, Agustin; Cruz-Diaz, David; Martinez-Amat, A [illegible]
The role of IL-6 in exercise-induced anorexia in normal-weight boys	Hunschede, Sascha; Schwartz, Alexander; Kubant, Ruslan; Thomas, Scott G.; Anderson, G. Harvey
Saliva cortisol in school children after acute physical exercise	Budde, Henning; Windisch, Claudia; Kudielka, Brigitte M.; Voelcker-Rehage, Claudia
Salivary and Plasmatic Antioxidant Profile following Continuous, Resistance, and High-Intensity Interval Exercise: Preliminary Study	Souza, Adriele V.; Giolo, Jessica S.; Teixeira, Renata R.; Vilela, Danielle D.; Peixoto, Leonardo G.; Justino, Allisson B.; Caixeta, Douglas C.; Puga, Guilherme M.; [illegible]
Salivary BDNF and Cortisol Responses During High-Intensity Exercise and Official Basketball Matches in Sedentary Individuals and Elite Players	Moreira, Alexandre; Aoki, Marcelo Saldanha; de Arruda, Ademir Felipe Schultz; Machado, Daniel Gomes da Silva; Elsangedy, Hassan Mohamed; Okano, Alexandre [illegible]
Salivary concentrations of IL-6 and IL-1ra after HIIT and MICT in young, healthy adults: A randomized exercise study	O'Neill, C. D.; O'Rourke, N.; Jeffrey, M.; Green-Johnson, J. M.; Dogra, S.
Salivary cortisol concentration after high-intensity interval exercise: Time of day and chronotype effect	Bonato, Matteo; La Torre, Antonio; Saresella, Marina; Marventano, Ivana; Merati, Giampiero; Vitale, Jacopo Antonino
Salivary α-amylase and IgA responses to repeated bouts of short high-intensity exercise tests in recreationally active men	Tateishi, K.; Kishimoto, A.; Higa, M.; Yasuda, N.
Salutary effects of moderate but not high intensity aerobic exercise training on the frequency of peripheral T-cells associated with immunosenescence in older women at high risk of breast cancer: a rando	Niemiro, Grace M.; Coletta, Adriana M.; Agha, Nadia H.; Mylabathula, Preteesh Leo; Baker, Forrest L.; Brewster, Abenaa M.; Bevers, Therese B.; Fuentes-Mattei, [illegible]
Satellite cell response to concurrent resistance exercise and high-intensity interval training in sedentary, overweight/obese, middle-aged individuals	Pugh, Jamie K.; Faulkner, Steve H.; Turner, Mark C.; Nimmo, Myra A.
Satiety responsiveness but not food reward is modified in response to an acute bout of low versus high intensity exercise in healthy adults	Thivel, D.; Fillon, A.; Genin, P. M.; Miguet, M.; Khammassi, M.; Pereira, B.; Boirie, Y.; Duclos, M.; Drapeau, V.; Blundell, J. E.; Tremblay, A.; Finlayson, G.; Mathi [illegible]
Selective effects of 8 weeks of high-intensity circuit training on inhibitory control in adult women	Yin, L. U. O.; Yumei, L. O. U.; Ya, D. O. U.; Yuntao, L. I.
Selenium supplementation and modified high-intensity interval training (m-HIIT) to modulate systemic oxidative stress in sedentary overweight/obese adults: in vitro and in vivo studies	Leighton, D.; Goua, M.; Burgess, K.; Bermano, G.
Self-measure of heart rate variability (HRV) and arrhythmia to monitor and to manage atrial arrhythmias: personal experience with high intensity interval exercise (HIIE) for the conversion to sinus rhythm	Young, David W.
Self-Myofascial Release Effect With Foam Rolling on Recovery After High-Intensity Interval Training	Laffaye, Guillaume; Da Silva, Debora Torrinha; Delafontaine, Arnaud
Self-regulatory biofeedback training: an intervention to reduce school burnout and improve cardiac functioning in college students	May, Ross W.; Seibert, Gregory S.; Sanchez-Gonzalez, Marcos A.; Fincham, Frank D.
Self-regulatory efficacy and long-term physical activity engagement: Examining mediators from a randomized trial	Dineen, Tineke E.; Locke, Sean R.; Cranston, Kaela D.; Beauchamp, Mark R.; Jung, Mary E.
Separate Effects of Exercise Amount and Intensity on Adipose Tissue and Skeletal Muscle Mass in Adults with Abdominal Obesity	Cowan, Theresa E.; Brennan, Andrea M.; Stotz, Paula J.; Clarke, John; Lamarche, Benoit; Ross, Robert
Separate Effects of Intensity and Amount of Exercise on Interindividual Cardiorespiratory Fitness Response	Ross, Robert; de Lannoy, Louise; Stotz, Paula J.
Serum testosterone response to high-intensity resistance training in male veteran sprint runners	Reaburn, P.; Logan, P.; Mackinnon, L.
Sex differences in adaptations to high intensity interval training	Hoffmann, S.; Skinner, T.; van Rosendal, S.; Osborne, M.; Emmerton, L.; Jenkins, D.
Short and long-term effects of high-intensity interval training applied alone or with whole-body cryostimulation on glucose homeostasis and myokine levels in overweight to obese subjects	Kozlowska-Flis, Marta; Rodziewicz-Flis, Ewa; Micielska, Katarzyna; Kortas, Jakub; Jaworska, Joanna; Borkowska, Andzelika; Sansoni, Veronica; Perego, Silvia; L [illegible]
Short and Sweet: HIIT Training in PE	Blackshear, Tara B.
Short bouts of high-intensity resistance-style training produce similar reductions in fasting blood glucose of diabetic offspring and controls	Russell, Ryan D.; Nelson, Arnold G.; Kraemer, Robert R.
A short period of high-intensity interval training improves skeletal muscle mitochondrial function and pulmonary oxygen uptake kinetics	Christensen, Peter M.; Jacobs, Robert A.; Bonne, Thomas; Fluck, Daniela; Bangsbo, Jens; Lundby, Carsten
Short-Term and Lifelong Exercise Training Lowers Inflammatory Mediators in Older Men	Hayes, Lawrence D.; Herbert, Peter; Sculthorpe, Nicholas F.; Grace, Fergal M.
Short-term community-based exercise programs in low-income older women: Does exercise intensity and modality matters?	Teixeira do Amaral, Vanessa; Fernandes, Bianca; Ngomane, Awassi Yuphiwa; Marcal, Isabela Roque; Zanini, Gabriel de Souza; Ciolac, Emmanuel Gomes
A Short-Term Eccentric HIIT Induced Greater Reduction in Cardio-Metabolic Risk Markers in Comparison With Concentric HIIT in Sedentary Overweight Men	Penailillo, L.; Diaz-Reiher, M.; Gurovich, A.; Flores-Opazo, M.
Short-term effects of two integrated, non-pharmacological body weight reduction programs on coronary heart disease risk factors in young obese patients	Sartorio, A.; Lafortuna, C. L.; Marinone, P. G.; Tavani, A.; La Vecchia, C.; Bosetti, C.
Short-Term Exercise Training Alters Leukocyte Chemokine Receptors in Obese Adults	Barry, Julianne C.; Simtchouk, Svetlana; Durrer, Cody; Jung, Mary E.; Little, Jonathan P.
Short-term exercise training reduces anti-inflammatory action of interleukin-10 in adults with obesity	Barry, Julianne C.; Simtchouk, Svetlana; Durrer, Cody; Jung, Mary E.; Mui, Alice L.; Little, Jonathan P.
Short-term exercise training reduces glycaemic variability and lowers circulating endothelial microparticles in overweight and obese women at elevated risk of type 2 diabetes	Rafiei, Hossein; Robinson, Emily; Barry, Julianne; Jung, Mary Elizabeth; Little, Jonathan Peter
Short-Term High-Intensity Circuit Training Does Not Modify Resting Heart Rate Variability in Adults during the COVID-19 Confinement	Garcia-Suarez, Patricia C.; Aburto-Corona, Jorge A.; Renteria, Ivan; Gomez-Miranda, Luis M.; Moncada-Jimenez, Jose; Lira, Fabio Santos; Antunes, Barbara Mou [illegible]
Short-term high-intensity interval and moderate-intensity continuous training reduce leukocyte TLR4 in inactive adults at elevated risk of type 2 diabetes	Robinson, Emily; Durrer, Cody; Simtchouk, Svetlana; Jung, Mary E.; Bourne, Jessica E.; Voth, Elizabeth; Little, Jonathan P.
Short-Term High-Intensity Interval Exercise Promotes Motor Cortex Plasticity and Executive Function in Sedentary Females	Hu, Min; Zeng, Ningning; Gu, Zhongke; Zheng, Yuqing; Xu, Kai; Xue, Lian; Leng, Lu; Lu, Xi; Shen, Ying; Huang, Junhao
Short-term High-intensity Interval Training and The Physiological Stress Response	Ormsbee, Michael J.; Kinsey, Amber W.; Chong, Minwook; Friedman, Heather S.; Dodge, Tonya; Fehling, Patricia C.
Short-Term High-Intensity Interval Training Is Superior to Moderate-Intensity Continuous Training in Improving Cardiac Autonomic Function in Children	van Biljon, Anneke; McKune, Andrew J.; DuBose, Katrina D.; Kolanisi, Unathi; Semple, Stuart J.
Short-term interval and continuous training improves pancreatic β-cell function adjusted for skeletal muscle insulin resistance in adults with prediabetes	Francois, M. E.; Eichner, N.; Gilbertson, N. M.; Heiston, E. M.; Barrett, E.; Malin, S. K.
A Short-Term Intervention of High-Intensity Exercise and Anodal-tDCS on Motor Learning in Middle-Aged Adults: An RCT	Quinlan, Clare; Rattray, Ben; Pryor, Disa; Northey, Joseph M.; Coxon, James; Cherbuin, Nicolas; Andrews, Sophie C.
Short-time high-intensity exercise increases peripheral BDNF in a physical fitness-dependent way in healthy men	Antunes, Barbara Moura; Rossi, Fabricio Eduardo; Teixeira, Ana Maria; Lira, Fabio Santos
Shorter recovery time following high-intensity interval training induced higher body fat loss among overweight women	Mirghani, Seyed Javad; Seydyousefi, Mehdi; Pekkala, Satu; Sharifian, Shohreh; Beyshami, Gheysar
A Shorter-Bout of HIIT Is More Effective to Promote Serum BDNF and VEGF-A Levels and Improve Cognitive Function in Healthy Young Men	Li, Qing; Zhang, Li; Zhang, Zhengguo; Wang, Yuhan; Zuo, Chongwen; Bo, Shumin
Similar Adaptations to 10 Weeks Concurrent Training on Metabolic Markers and Physical Performance in Young, Adult, and Older Adult Women	Andrade, David C.; Flores-Opazo, Marcelo; Penailillo, Luis; Delgado-Floody, Pedro; Cano-Montoya, Johnattan; Vasquez-Gomez, Jaime A.; Alvarez, Cristian
Similar cardiometabolic effects of high- and moderate-intensity training among apparently healthy inactive adults: a randomized clinical trial	Ramirez-Velez, Robinson; Tordecilla-Sanders, Alejandra; Tellez-T, Luis Andres; Camelo-Prieto, Diana; Hernandez-Quinonez, Paula Andrea; Correa-Bautista, Jorge [illegible]
Similar health benefits of endurance and high-intensity interval training in obese children	Corte de Araujo, Ana Carolina; Roschel, Hamilton; Picanco, Andreia Rossi; do Prado, Danilo Marcelo Leite; Villares, Sandra Mara Ferreira; de Sa Pinto, Ana Lucia; [illegible]
Similar perceptual responses to reduced exertion high intensity interval training (REHIT) in adults differing in cardiorespiratory fitness	Astorino, Todd A.; Clausen, Rasmus; Marroquin, Joel; Arthur, Balegh; Stiles, Kevin
Similar Postexercise Hypotension After MICT, HIIT, and SIT Exercises in Middle-Age Adults	McCarthy, Seth F.; Ferguson, Emily J.; Jarosz, Claudia; Kenno, Kenji A.; Hazell, Tom J.
Similar Responses of Circulating MicroRNAs to Acute High-Intensity Interval Exercise and Vigorous-Intensity Continuous Exercise	Cui, Shu F.; Wang, Cheng; Yin, Xin; Tian, Dong; Lu, Qiu J.; Zhang, Chen Y.; Chen, Xi; Ma, Ji Z.
A single bout of high-intensity interval exercise and work-matched moderate-intensity exercise has minimal effect on glucose tolerance and insulin sensitivity in 7- to 10-year-old boys	Cockcroft, Emma J.; Williams, Craig A.; Jackman, Sarah R.; Bassi, Shikhar; Armstrong, Neil; Barker, Alan R.
A single session of low-volume high-intensity interval and moderate-intensity continuous exercise elicits a transient reduction in ghrelin levels, but not in post-exercise energy intake in obese men	Matos, Victor Araujo Ferreira; de Souza, Daniel Costa; Browne, Rodrigo Alberto Vieira; Dos Santos, Victor Oliveira Albuquerque; Medeiros, Italo Freire; do Nascime [illegible]
Six HIT Sessions Improve Cardiorespiratory Fitness and Metabolic Flexibility in Insulin Resistant and Insulin Sensitive Adolescents with Obesity	Aparecido, Juliana Monique Lino; Marquezi, Marcelo Luis; Couto, Hellyelson Lopes de Omena; Santos, Thais Mitta da Silva; Cruz, Alison Fabiano Cunha; Lopes, [illegible]
Six weeks of high intensity interval training (HIIT) facilitates a four year preservation of aerobic capacity in sedentary older males: A reunion study	Herbert, Peter; Hayes, Lawrence D.; Beaumont, Alexander J.; Grace, Fergal M.; Sculthorpe, Nicholas F.
Six weeks of high-intensity interval training enhances contractile activity induced vascular reactivity and skeletal muscle perfusion in older adults	Hearon, Philip J. J.; Atherton, Philip J.; Smith, Kenneth; Williams, John P.; Lund, Jonathan N.; Phillips, Bethan E.
Six weeks of high-intensity interval training to exhaustion attenuates dynamic cerebral autoregulation without influencing resting cerebral blood velocity in young fit men	Drapeau, Audrey; Labrecque, Lawrence; Imhoff, Sarah; Paquette, Myriam; Le Blanc, Olivier; Malenfant, Simon; Brassard, Patrice
SIX WEEKS OF HIGH-INTENSITY INTERVAL TRAINING WITH AND WITHOUT β-ALANINE SUPPLEMENTATION FOR IMPROVING CARDIOVASCULAR FITNESS IN WOMEN	Walter, Ashley A.; Smith, Abbie E.; Kendall, Kristina L.; Stout, Jeffrey R.; Cramer, Joel T.
Skeletal Muscle Adaptive Responses to Different Types of Short-Term Exercise Training and Detraining in Middle-Age Men	Callahan, Marcus J.; Parr, Evelyn B.; Snijders, Tim; Conceicao, Miguel S.; Radford, Bridget E.; Timmins, Ryan G.; Devlin, Brooke L.; Hawley, John A.; Camera, D [illegible]
Skeletal muscle fat metabolism after exercise in humans: influence of fat availability	Kimber, Nicholas E.; Cameron-Smith, David; McGee, Sean L.; Hargreaves, Mark
Skeletal muscle metabolism during short duration high-intensity exercise influence of creatine supplementation (Metabolisme du muscle squelettique lors d'un exercice bref et de forte intensite : influence	Balsom, P. D.; Soderlund, K.; Sjodin, B.; Ekblom, B.
Skeletal Muscle Mitochondrial and Perilipin Content in a Cohort of Obese Subjects Undergoing Moderate and High Intensity Training	Sirago, Giuseppe; Vaccari, Filippo; Lazzer, Stefano; D'Amuri, Andrea; Sanz, Juana M.; Narici, Marco V.; Reggiani, Carlo; Passaro, Angelina; Toniolo, Luana
Skeletal muscle mitochondrial DNA content in exercising humans	Marcuello, A.; Gonzalez-Alonso, J.; Calbet, J. A. L.; Damsgaard, R.; Lopez-Perez, M. J.; Diez-Sanchez, C.
SMALL-SIDED GAMES VERSUS INTERVAL TRAINING IN ADOLESCENT SOCCER PLAYERS: EFFECTS ON BODY COMPOSITION. / IGRE NA MALOM PROSTORU (SSG) NASUPROT INTERVALNOM TRENINGU KOD	Gušić, Marko; Stanković, Mima; Radanović, Danilo; Andrašić, Slobodan; Molnar, Slavko
Soldiers' Heart: A Prospective Study of Cardiac Remodeling in Soldiers Undergoing Progressive Intensity Exercise Training	Stanton, Kelly M.; Wylie, Laura; Kotchetkova, Irina; Coy, Amy; Carroll, Gerard; La Gerche, Andre; Celermajer, David S.
Specific wushu sanda high-intensity interval training protocol improved physical fitness of amateur athletes': A pilot study. / Un protocolo específico de entrenamiento interválico de alta intensidad de wus	Farias Monteiro, Jorge Rafael; Boscolo del Vecchio, Fabricio; Vasconcelos, Breno Berny; Silveira Coswig, Victor
SPRINT INTERVAL TRAINING VS. HIGH INTENSITY INTERVAL TRAINING IN UNTRAINED UNIVERSITY STUDENTS	Boer, Pieter-Henk
Sprint running & burpees: comparison of acute physiological and neuromuscular effects of two high intensity interval training protocols	Bingley, S.; Witchalls, J.; McKune, A.; Humberstone, C.
Strength training combined with high-intensity interval aerobic training in young adults' body composition	Alves, Ana Ruivo; Marinho, Daniel Almeida; PecÉGo, Marco; Ferraz, Ricardo; Marques, Mario Cardoso; Neiva, Henrique Pereira
A study of intensity, fatigue and precision in two specific interval trainings in young tennis players: high-intensity interval training versus intermittent interval training	Suarez Rodriguez, David; Del Valle Soto, Miguel
The sub-acute effects of high-intensity interval training in healthy young adults: respiratory parameters, aerobic capacity and perceived stress	Yalman, Ali; Telli Atalay, Orcin; Unver, Fatma; Senol, Hande; Taskin, Harun
Subjective exercise experiences during and after high and low intensity exercise in active and inactive adult females: some preliminary findings. / Experiences subjectives de l ' activite physique, pendant e	Daley, A. J.; Welch, A.
Substrate utilization during and after high intensity exercise in healthy lean and obese men	Santiworakul, Anoma; Chuaychoo, Benjamas; Kriengsinyos, Wantanee; Saengsirisuwan, Vitoon; Jalayondeja, Wattana
Superior Effects of High-Intensity Interval Training vs. Moderate Continuous Training on Arterial Stiffness in Episodic Migraine: A Randomized Controlled Trial	Hanssen, Henner; Minghetti, Alice; Magon, Stefano; Rossmeissl, Anja; Papadopoulou, Athina; Klenk, Christopher; Schmidt-Trucksass, Arno; Faude, Oliver; Zahno [illegible]
A SUPLEMENTAÇÃO DE CREATINA AUMENTA A EXPRESSÃO DO RECEPTOR DO IGF-1 EM TECIDO MUSCULAR DE RATOS WISTAR TREINADOS COM EXERCÍCIO INTERVALADO DE ALTA INTENSIDADE. / Creat	Haar Flores, Carlos Eduardo; Czarnabay, Débora; Nedersauer Leote, Jéssica; Simoni Brum, Ilma; von Eye Corletta, Helena; Capp, Edison; Dias Ferreira, Gustavo
Suspension Training HIIT Improves Gait Speed, Strength and Quality of Life in Older Adults	Jimenez-Garcia, Jose Daniel; Martinez-Amat, Antonio; De la Torre-Cruz, M. J.; Fabrega-Cuadros, Raquel; Cruz-Diaz, David; Aibar-Almazan, Agustin; Achalandaba [illegible]
Sustained improvements in cardiac function and fitness one year after an exercise intervention in obese children	Charlotte Bjork Ingul, C.; Dias, K.; Hosseini, M.; Hollekim-Strand, S. M.; Follestad, T.; Davies, P. S.; Tjonna, A. E.; Coombes, J.
Swing and a miss or inside-the-park home run: which fate awaits high-intensity exercise training?	Keteyian, Steven J.
Targeted High-Intensity Exercise With And Without Osteoporosis Medications To Reduce Fracture Risk In Postmenopausal Women	Kistler-Fischbacher, Melanie; Yong, Jedidah S.; Weeks, Benjamin K.; Beck, Belinda R.
Telesna vadba za ektomorfni somatotip človeka - študija primera. / Physical exercise for the human ectomorph somatotype - case study	Okrožnik, Katarina; Šuštaršič, Ana; Dolenc, Maja
Tensiomyographic Markers Are Not Sensitive for Monitoring Muscle Fatigue in Elite Youth Athletes: A Pilot Study	Wiewelhove, Thimo; Raeder, Christian; de Paula Simola, Rauno Alvaro; Schneider, Christoph; Doweling, Alexander; Ferrauti, Alexander
Testosterone and Cortisol Responses to Five High-Intensity Functional Training Competition Workouts in Recreationally Active Adults	Mangine, Gerald T.; Van Dusseldorp, Trisha A.; Feito, Yuri; Holmes, Alyssa J.; Serafini, Paul R.; Box, Allyson G.; Gonzalez, Adam M.
Thirst responses following high intensity intermittent exercise when access to ad libitum water intake was permitted, not permitted or delayed	Mears, Stephen A.; Watson, Phillip; Shirreffs, Susan M.
Time course for strength and muscle thickness changes following upper and lower body resistance training in men and women	Abe, T.; DeHoyos, D. V.; Pollock, M. L.; Garzarella, L.
Time Course of Recovery Following CrossFit R Karen Benchmark Workout in Trained Men	de Sousa Neto, Ivo Vieira; de Sousa, Nuno Manuel Frade; Neto, Frederico Ribeiro; Falk Neto, Joao Henrique; Tibana, Ramires Alsamir
Time-course changes of oxidative stress response to high-intensity discontinuous training versus moderate-intensity continuous training in masters runners	Vezzoli, Alessandra; Pugliese, Lorenzo; Marzorati, Mauro; Serpiello, Fabio Rubens; La Torre, Antonio; Porcelli, Simone
Time-efficient high-intensity exercise improves glycaemic control similarly to moderate-intensity exercise in overweight humans	Parker, L.; Shaw, C.; Banting, L.; Hill, K.; McAinch, A.; Levinger, I.; Stepto, N.
Time-efficient physical activity intervention for older adolescents: the Burn 2 Learn cluster randomised controlled trial	Costigan, S.; Eather, N.; Gyawali, P.; Hillman, C.; Holliday, E.; Kennedy, S.; Leahy, A.; Lonsdale, C.; Lubans, D.; Mavilidi, M.; et al.,
Timing of high-intensity intermittent exercise affects ad libitum energy intake in overweight inactive men	Panissa, Valeria Leme Goncalves; Julio, Ursula Ferreira; St-Pierre, David H.; Tavares da Silva Gomes, Alicia; Caldeira, Renan Santos; Lira, Fabio Santos; Takito, Mo [illegible]
Toward a neuroprotective shift: Eight weeks of high intensity interval training reduces the neurotoxic kynurenine activity concurrently to impulsivity in emotionally impulsive humans - A randomized contro	Javelle, Florian; Bloch, Wilhelm; Knoop, Andre; Guillemin, Gilles J.; Zimmer, Philipp
Training session intensity affects plasma redox status in amateur rhythmic gymnasts	Bellafiore, Marianna; Bianco, Antonino; Battaglia, Giuseppe; Naccari, Maria Silvia; Caramazza, Giovanni; Padulo, Johnny; Chamari, Karim; Paoli, Antonio; Palma, A [illegible]
Tribulus terrestris extracts alleviate muscle damage and promote anaerobic performance of trained male boxers and its mechanisms: Roles of androgen, IGF-1, and IGF binding protein-3	Ma, Yiming; Guo, Zhicheng; Wang, Xiaohui
Twelve minutes/week of high-intensity exercise significantly decreases aortic reservoir pressure in individuals with metabolic syndrome	Ramos, J.; Ramos, M.; Dalleck, L.; Borrani, F.; Walker, K.; Fassett, R.; Sharman, J.; Coombes, J.
Twelve Weeks Of Resistance Training And High Intensity Interval Training Improves Physical Employment Standards Performance: 539	Sterczala, Adam J.; Krajewski, Kellen T.; Peterson, Patrick A.; Sekel, Nicole M.; Lovalekar, Mita; Wardle, Sophie L.; O'Leary, Thomas J.; Greeves, Julie P.; Flanaga [illegible]
Twenty-four-hour cortisol response to multiple daily exercise sessions of moderate and high intensity	Hackney, A. C.; Viru, A.
Two weeks of exercise training intensity on appetite regulation in obese adults with prediabetes	Heiston, Emily M.; Eichner, Natalie Z. M.; Gilbertson, Nicole M.; Gaitan, Julian M.; Kranz, Sibylle; Weltman, Arthur; Malin, Steven K.
Two Weeks of High-Intensity Interval Training in Combination With a Non-thermal Diffuse Ultrasound Device Improves Lipid Profile and Reduces Body Fat Percentage in Overweight Women	Hausswirth, Christophe; Marquet, Laurie-Anne; Nesi, Xavier; Slattery, Katie
Two weeks of high-intensity interval training increases skeletal muscle mitochondrial respiration via complex-specific remodeling in sedentary humans	Batterson, Philip M.; McGowan, Erin M.; Stierwalt, Harrison D.; Ehrlicher, Sarah E.; Newsom, Sean A.; Robinson, Matthew M.
Two Weeks of Interval Training Enhances Fat Oxidation during Exercise in Obese Adults with Prediabetes	Gaitan, Julian M.; Eichner, Natalie Z. M.; Gilbertson, Nicole M.; Heiston, Emily M.; Weltman, Arthur; Malin, Steven K.
Two weeks of moderate-intensity continuous training, but not high-intensity interval training, increases insulin-stimulated intestinal glucose uptake	Motiani, Kumail K.; Savolainen, Anna M.; Eskelinen, Jari-Joonas; Toivanen, Jussi; Ishizu, Tamiko; Yli-Karjanmaa, Minna; Virtanen, Kirsi A.; Parkkola, Riitta; Kapan [illegible]
Type of supervision does not affect attendance or physical fitness in an 8-week workplace exercise intervention	Hunter, J.; Lythgo, N.; Gordon, B.; Benson, A.
Tyre-pulling as concurrent training	Mamen, Asgeir; Fredriksen, Per Morten
Upper Body High Intensity Short Term Interval Training: Effects on Aerobic and Anaerobic Fitness	Bernardi, Marco; Carucci, Silvia; Fattorini, Luigi; Squeo, Maria Rosaria; Lanzano, Riccardo; Adami, Paolo Emilio; Bhambhani, Yagesh
The use of a smart-textile garment during high-intensity functional training: a pilot study	Feito, Yuri; Moriarty, Terence A.; Mangine, Gerald; Monahan, Jessica
The Use of Autonomic Modulation Device to Control Training Performance after High-Intensity Interval Training Program	Clemente-Suarez, Vicente Javier; Arroyo-Toledo, Juan Jaime
Using Inertial and Physiological Sensors to Investigate the Effects of a High-Intensity Interval Training and Plyometric Program on the Performance of Young Judokas	Manas-Paris, Adrian; Muyor, Jose M.; Oliva-Lozano, Jose M.
Using Peripheral Blood Mononuclear Cells To Assess Acute Effects Of High-intensity Interval Exercise On Mitochondrial Function: 1120	Wilburn, Dylan; Ismaeel, Ahmed; Fletcher, Emma; Torres, Ricardo; Adair, Kathleen; Lowry, Catherine; Beeson, Cassidy; Forsse, Jeffrey S.; Koutakis, Panagiotis
Utility and feasibility of a smartphone app to support school-based high-intensity interval training (HIIT): Findings from the Burn 2 Learn cluster RCT	Leahy, A.; Smith, J.; Eather, N.; Morgan, P.; Lonsdale, C.; Noetel, M.; Plotnikoff, R.; Duncan, M.; Blackburn, S.; Lubans, D.
Validation of the Subjective Perception of Exertion Scales with an Interval Training Protocol	Teixeira, J. A. A.; Ferraz, E. J.; Teixeira, K. K. L.; Assi, R. S. C.; Lino, A. D. S.; Figueira, T. G.
Vascular function is unaltered after aerobic acute exercise in physically active young and older male adults	Marocco, J. L.; Pinto, M.; Santa-Clara, H.; Fernhall, B.; Melo, X.
Verbal Encouragement and Between-Day Reliability During High-Intensity Functional Strength and Endurance Performance Testing	Engel, Florian A.; Faude, Oliver; Kolling, Sarah; Kellmann, Michael; Donath, Lars
Very high-intensity exercise decreases MCT1 and MCT4 content in human skeletal muscle. (Abstract)	Bishop, D.; Thomas, C.; Edge, J.; Mercier, J.
Vigorous-intensity acute exercise during encoding can reduce levels of episodic and false memory	Loprinzi, Paul; Javadi, Amir-Homayoun; Jung, Myungjin; Watson, Hanna; Sanderson, Claire; Kang, Minsoo; Kelemen, William L.
Visceral Fat Mass Of Overweight Males Following 12 Weeks Of High Intensity Intermittent Exercise	Heydari, Mehrdad; Freund, Judith; Boutcher, Stephen H.
VISOKO INTENZIVNI INTERVALNI TRENING U UPOREDBI S TRENINGOM KONSTANTNOG INTENZITETA U PRETILE DJECE. / HIGH INTENSITY INTERVAL TRAINING IN COMPARISON TO CONSTANT LOAD TRAINI	Valjan, Boris; Vidović, Mandica; La Grasta, Lavinija; Ružić, Lana
Vo2max Improvement After High-intensity Interval Training Is Not High-intensity Dependent In Metabolic Syndrome Patients	Ortega, Juan F.; Moreno-Cabañas, Alfonso; Morales-Palomo, Félix; Ramírez-Jiménez, Miguel; Alvarez-Jiménez, Laura; Mora-Rodríguez, Ricardo
Was it a HIIT? A process evaluation of a school-based high-intensity interval training intervention	Duncombe, S.; Barker, A. R.; Price, L.; Walker, J. L.; Stylianou, M.
Water-based Training Programs Improve Functional Capacity, Cognitive and Hemodynamic Outcomes? The ACTIVE Randomized Clinical Trial	Hafele, M. S.; Alberton, C. L.; Hafele, V.; Schaun, G. Z.; Nunes, G. N.; Calonego, C.; Castro, T. F.; Andrade, L. S.; Pinto, S. S.
Weight and plyometric training: effects on eccentric and concentric force production	Wilson, G. J.; Murphy, A. J.; Giorgi, A.
When It HIITs, You Feel No Pain: Psychological and Psychophysiological Effects of Respite-Active Music in High-Intensity Interval Training	Karageorghis, Costas I.; Jones, Leighton; Howard, Luke W.; Thomas, Rhys M.; Moulashis, Panayiotis; Santich, Sam J.
Where does HIT fit? An examination of the affective response to high-intensity intervals in comparison to continuous moderate- and continuous vigorous-intensity exercise in the exercise intensity-affect co	Jung, Mary E.; Bourne, Jessica E.; Little, Jonathan P.
Whole body water after 16 weeks of high intensity interval training in Metabolic Syndrome patients	Ortega Fonseca, JF; Morales Palomo, F.; Ramirez Jimenez, M.; Fernandez Elias, V.; Guio de Prada, V.; Mora Rodriguez, R.
Women undergoing assisted fertilisation and high-intensity interval training: a pilot randomised controlled trial	Kiel, Ida Almenning; Lundgren, Kari Magrethe; Morkved, Siv; Kjotrod, Sigrun Beate; Salvesen, Oyvind; Romundstad, Liv Bente; Moholdt, Trine
Women with metabolic syndrome show similar health benefits from high-intensity interval training than men	Guio de Prada, Valle; Ortega, Juan Fernando; Morales-Palomo, Felix; Ramirez-Jimenez, Miguel; Moreno-Cabanas, Alfonso; Mora-Rodriguez, Ricardo
Work Rate Adjustments, Cardiovascular Strain, And Thermal Strain During High-intensity Interval Exercise In The Heat: 991	Yoder, Hillary A.; Mulholland, Anne M.; Barefoot, Nick T.; Sullivan, Katie; MacDonald, Hayley V.; Wingo, Jonathan E.
YÜKSEK ŞİDDETLİ İNTERVAL ANTRENMAN PROGRAMININ FİZYOLOJİK PARAMETRELER VE SOLUNUM KAS KUVVETİ ÜZERİNE ETKİSİ. / THE EFFECT OF HIGH INTENSITY INTERVAL TRAINING PROGRAM ON P	Bostanci, Özgür; Mayda, Muhammet Hakan; Tosun, Mehmet İsmail; Kabadayı, Menderes
[illegible] g on gremlin-1, Macrophage migration inhibitory factor and some cardiovascular risk factors in obese men / [illegible]	
[illegible] ity continuous training on inflammatory biomarkers of cardiovascular disease predictor in obese menopause women / [illegible]	
[illegible] / Effects of Different Intensive Intermittent Training on Maximum Oxygen Uptake and Output Power of Elite Road Cyclists	[illegible]
