# Supplementary material for: Solving the High-Intensity Multimodal Training Prescription Puzzle: A Systematic Mapping Review
Source: Sports Med Open. 2024 Jul 23;10:82. doi: 10.1186/s40798-024-00747-z (PMC11263329; doi:10.1186/s40798-024-00747-z)
Supplement: Supplementary file 5 — Supplementary Material 5 [file 40798_2024_747_MOESM5_ESM.pdf]

Sports Medicine Open

Title: Solving the High-Intensity Multimodal Training Prescription Puzzle: A Systematic Mapping Review.

Tijana Sharp[1](0000-0001-6878-6343); Katie Slattery[1], Aaron J. Coutts[1]; Mikah v Gogh[2], Lara Ralph[1], Lee Wallace[1]

[1]School of Sport, Exercise and Rehabilitation, University of Technology, Sydney, Human Performance Research Centre Moore Park, Sydney, Australia, [2]Australian College of Physical Education, 10 Parkview Dr, Sydney Olympic Park, Sydney, Australia

All supplementary materials including data extracted from included studies are available online (osf.io/yknq4).

Supplementary Table S5 Country of Origin of Included Publications

Country of Origin	Author Affiliation (n)	Participants (n)
Australia	10	10
Austria	1	1
Brazil	33	32
Canada	8	8
Chile	1	1
China	4	5
Colombia	1	2
Czech Republic	1	1
Denmark	1	1
Germany	12	12
Greece	8	8
Indonesia	2	2
Iran	8	9
Ireland	1	1
Israel	1	1
Italy	5	5
Japan	2	2
Korea	1	1
Malaysia	2	2
Mexico	1	1
Netherlands	1	1
New Zealand	1	2
Pakistan	1	1
Poland	10	10
Portugal	1	1
Serbia	0	1
Slovenia	1	0
Spain	24	23
Switzerland	1	1
Taiwan	1	1
Thailand	3	3
Turkey	1	1
UK	10	9
Ukraine	1	1
USA	61	60
