# Supplementary material for: Solving the High-Intensity Multimodal Training Prescription Puzzle: A Systematic Mapping Review
Source: Sports Med Open. 2024 Jul 23;10:82. doi: 10.1186/s40798-024-00747-z (PMC11263329; doi:10.1186/s40798-024-00747-z)
Supplement: Supplementary file 6 — Supplementary Material 6 [file 40798_2024_747_MOESM6_ESM.pdf]

Sports Medicine Open

Title: Solving the High-Intensity Multimodal Training Prescription Puzzle: A Systematic Mapping Review.

Tijana Sharp[1](0000-0001-6878-6343); Katie Slattery[1], Aaron J. Coutts[1]; Mikah v Gogh[2], Lara Ralph[1], Lee Wallace[1]

[1]School of Sport, Exercise and Rehabilitation, University of Technology, Sydney, Human Performance Research Centre Moore Park, Sydney, Australia, [2]Australian College of Physical Education, 10 Parkview Dr, Sydney Olympic Park, Sydney, Australia

All supplementary materials including data extracted from included studies are available online (osf.io/yknq4).

Supplementary Table S6 Exercise Reporting Recommendations for High-Intensity Multimodal Training

	Prescriptive consideration	Reporting items*	Reporting example
**Training variables**	Exercise Selection	Description of:<br>• all exercises to enable replication<br>• rest period modality/ exercise	Photographs, video footage of each exercise prescribed. May be supplied as supplementary material.
	Exercise Order	Description of the structure of the exercise session/s and order exercises are performed	"Exercises were repeated in a circuit format", "3 sets of each exercise were performed before moving on", "AMRAP"
	Intervention duration	Description of the duration of a training program/ intervention (including familiarisation weeks where appropriate)	"2 weeks of familiarisation, followed by 8 weeks of training"
	Frequency	Description of training frequency across a week or intervention	"3 days/ week"
	Volume	Description of the:<br>• Total session duration<br>• Net work duration<br>• Single work period duration<br>• Single rest period duration<br>• Work: rest ratio<br>• Sets/ rounds<br>• Repetitions<br>• External load (e.g., kg, lbs)<br>• Distance/ height/ work (e.g., m, watts)<br>Reporting may focus on time based or sets/ reps based prescription relative to the session and/ or program.	• "45 minutes"<br>• "30 minutes work"<br>• "60 seconds"<br>• "60 seconds"<br>• "1:1"<br>• "5 rounds of a circuit"<br>• "15 reps of each exercise"<br>• "12kg KB swing"<br>• "200m run", "40cm box jump", "10 calories"
	Intensity	Description of the prescribed:<br>• Average session objective† intensity<br>• Average session subjective# intensity<br>• Work period objective intensity<br>• Work period subjective intensity<br>• Rest period objective intensity<br>• Rest period subjective intensity	• "80% HR_{max}"<br>• "8/10 RPE", "all out", "AMRAP"<br>• "80% HR_{max}", "75% 1RM"<br>• "8/10 RPE", "all out"<br>• "50% HR_{max}"<br>• "5/10 RPE", "transition phase"
	Monitoring	Description of the methods used to monitor exercise volume and/or intensity:<br>• Objective intensity measures<br>• Subjective intensity measures<br>• Record of volume achieved<br>• Record of modifications or adjustments to prescription made	• "% HR_{max}"<br>• "RPE"<br>• "Sets, reps, time recorded"<br>• "Exercise/ volume/ intensity modifications, adverse events"
	Progression/ regression	Description of:<br>• Rationale for starting level of exercise<br>• Progression method<br>• Progression rationale<br>• Regression method<br>• Regression rationale	• "50% HR_{max} achieved in pre-testing"<br>• "Increased external load"<br>• "RPE <8/10 following the final set"<br>• "Reduced exercise complexity"<br>• "Inappropriate exercise technique"
	Other	Description of the:<br>• Exercise tempo<br>• Warm up<br>• Cool down<br>• Nutritional intake control	• "As fast as possible"<br>• "3 minutes of brisk walking (6km/h)"<br>• "3 minutes of slow walking (4km/h)"<br>• "Nutritional intake was not controlled for"

		• Home exercise program	• "Refrain from additional physical activity"
**Non-training variables**	Personnel involved in prescription, supervision and monitoring of exercise	Description of the qualifications and experience of the person/s:<br>• Prescribing (i.e., pre-planning of the intervention session/s)<br>• Supervising (i.e., supervising the room for safety purposes, may not be involved in leading and/ or monitoring)<br>• Leading (i.e., delivering the exercise protocol)<br>• Monitoring exercise (i.e., measures and/ or collects specified metrics e.g., heart rate)	Accredited Exercise Scientist (3 years),<br>Level 2 CrossFit ® certification (10 years),<br>Certificate III & IV in fitness (5 years)
	Group environment	Description of:<br>• The group environment exercise is performed in (i.e., other people in the room)<br>• How many individuals/ group	• "Individually", "partners", "groups", "choice of partner or group"<br>• "5-6 participants/ group"
	Psycho-social factors	Description of the:<br>• Strategies for motivation/ encouragement<br>• Music selection<br>• Technical cueing related to exercise form<br>• Strategies to increase adherence/ compliance/ retention in intervention	• "Verbal encouragement", "HR displayed on television", "team based workouts"<br>• "Pop music", "130bpm"<br>• "Staff corrected improper technique"<br>• "Self-selection of exercise order", "purposeful variety in sessions"
	Physical environment	Description of the:<br>• Physical location of training<br>• Equipment used to enable replication	• "Indoor gym facility", "university laboratory"<br>• Detailed list and/ or photographs of equipment

*Authors should seek to report as much detail as possibly to enable protocol replication. It is acknowledged that each reporting item may not be relevant to every HIMT protocol and authors should use their best judgement to determine which items are to be reported.

AMRAP, *as many reps as possible,* kg, *kilograms,* lb, *pounds,* m, *metres,* KB, *kettlebell,* cm, *centimetres,* HR_{max}, *heart rate maximum,* RPE, *rating of perceived exertion,* 1RM, *one repetition maximum,* km/h, *kilometres per hour,* †, *may include measures such as relative heart rate, percentage repetition* maximum, #, *may include measures such as rating of perceived exertion*
